# Benzo[4,5]cyclohepta[1,2-*b*]fluorene: an isomeric motif for pentacene containing linearly fused five-, six- and seven-membered rings†
†Electronic supplementary information (ESI) available: Synthetic procedures and characterization data for all new compounds; general experimental method; additional spectroscopic data; DFT calculation details; crystallographic data; and OTFT characterizations. CCDC 1468926 and 1468927. For ESI and crystallographic data in CIF or other electronic format see DOI: 10.1039/c6sc01795a


**DOI:** 10.1039/c6sc01795a

**Published:** 2016-06-07

**Authors:** Xuejin Yang, Xueliang Shi, Naoki Aratani, Théo P. Gonçalves, Kuo-Wei Huang, Hiroko Yamada, Chunyan Chi, Qian Miao

**Affiliations:** a Department of Chemistry , The Chinese University of Hong Kong , Shatin, New Territories , Hong Kong , China . Email: miaoqian@cuhk.edu.hk; b Department of Chemistry , National University of Singapore , 3 Science Drive 3 , 117543 , Singapore . Email: chmcc@nus.edu.sg; c Graduate School of Materials Science , Nara Institute of Science and Technology (NAIST) , 8916-5 Takayama-cho , Ikoma 630-0192 , Japan; d Division of Physical Science and Engineering and KAUST Catalysis Center , King Abdullah University of Science and Technology (KAUST) , Thuwal 23955-6900 , Kingdom of Saudi Arabia

## Abstract

Benzo[4,5]cyclohepta[1,2-*b*]fluorene (**5a**), a new π-conjugated polycyclic hydrocarbon containing linearly fused six-, five-, six-, seven- and six-membered rings (C_6_–C_5_–C_6_–C_7_–C_6_), was designed and its stable derivatives **5b** and **5c** were synthesized. With 22 π electrons, **5a** is an isomer of pentacene with quinoidal, dipolar ionic and diradical resonance forms. Molecules **5b** and **5c** were experimentally investigated with cyclic voltammetry, electronic absorption spectroscopy and X-ray crystallographic analysis, and theoretically studied by calculating the NICS value, diradical character and dipole moment. A comparison of **5a–c** with pentacene and other pentacene analogues containing linearly fused five- or seven-membered rings was also conducted and discussed. It was found that **5b** behaved as a p-type organic semiconductor in solution-processed thin film transistors with a field effect mobility of up to 0.025 cm^2^ V^–1^ s^–1^.

## Abstract 2

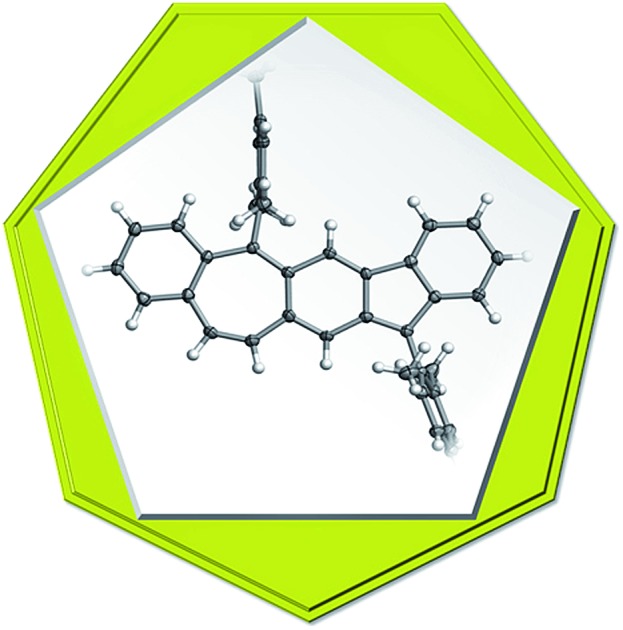
A new class of conjugated polycyclic molecules containing a C_6_–C_5_–C_6_–C_7_–C_6_ polycylic framework was synthesized. Both experiments and calculations show different electron structures in comparison to their pentacene isomers.

## Introduction

**Fig. 1:**
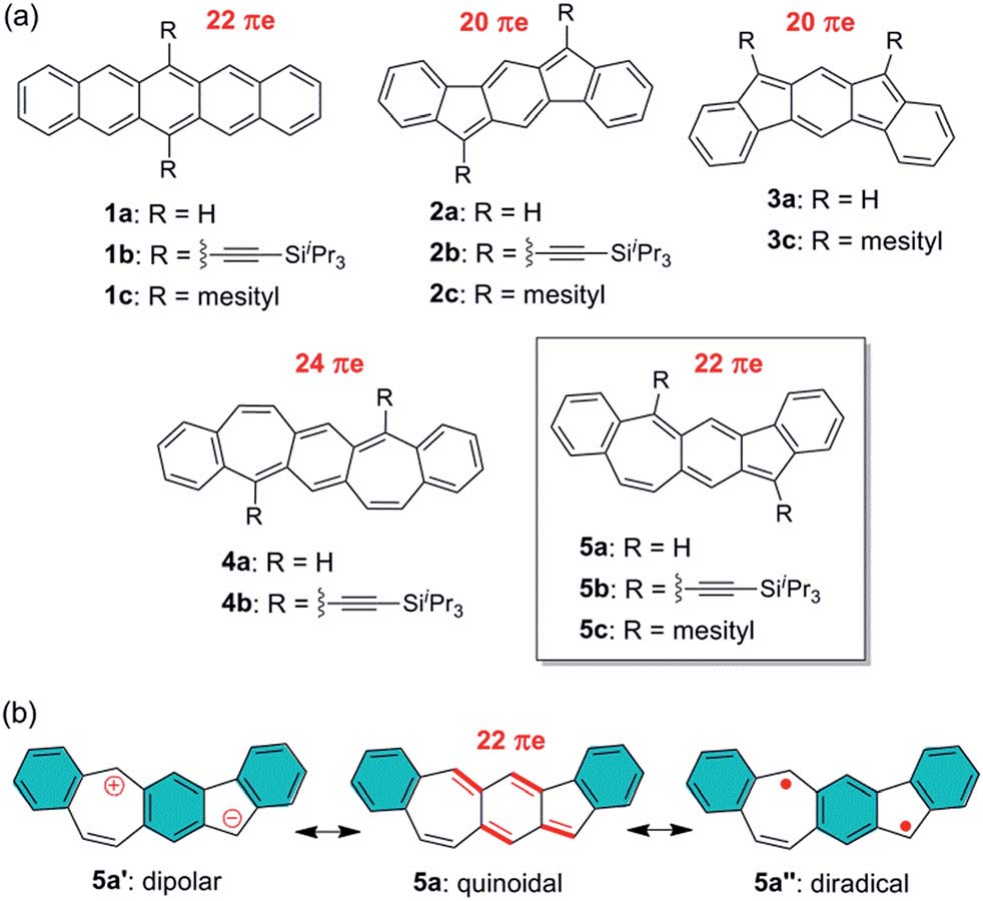
(a) Chemical structures of pentacene and its analogues; (b) three typical resonance forms of **5a**.

Pentacene (**1a** in Fig. 1a) is a leading p-type organic semiconductor for applications in light-weight, flexible and low-cost organic electronic devices,^1^ and has been used as a benchmark in comparison with new materials for applications in organic thin film transistors (OTFTs).^2^ Pentacene has been molecularly engineered with three strategies in order to modify electronic structure, tune molecular packing in the solid state, improve solubility and stability, and better understand its structure–property relationship. As extensively studied, the first strategy is to substitute H atoms in pentacene with a variety of functional groups.^3^ The most successful example of this strategy is 6,13-bis((triisopropylsilyl)ethynyl)-pentacene (**1b** in Fig. 1a),^4^ which is a solution-processed high-mobility p-type semiconductor^5^,^6^ with brickwork arrangement of π-planes. The second strategy is to replace C atoms in pentacene with hetero atoms, such as B,^7^ N,^8^ and S.^9^,^10^ Among the resultant heteropentacenes, *N*-heteropentacenes were most extensively studied, and have recently arisen as a class of organic semiconductors with high performance in OTFTs.^11^ The third strategy is to replace six-membered rings in pentacene with five- or seven-membered rings, leading to recently reported pentacene analogues containing C_6_–C_5_–C_6_–C_5_–C_6_^12^–^14^ and C_6_–C_7_–C_6_–C_7_–C_6_^15^ polycyclic frameworks, such as **2–4** in Fig. 1a. With 20 π electrons, **2a** and **3a** both have two π electrons less than pentacene, while **4a** has two more π electrons. Therefore, their electronic structure and physical properties are distinctively different from those of pentacene. In this study, we explore a novel linearly fused pentacene analogue, benzo[4,5]cyclohepta[1,2-*b*]fluorene (**5a** in Fig. 1a), which contains an unprecedented C_6_–C_5_–C_6_–C_7_–C_6_ polycyclic framework. Unlike other pentacene analogues, **5a** is a constitutional isomer of pentacene having both five- and seven-membered rings in the linear π-backbone with 22 π electrons. Besides the quinoidal resonance structure, one dipolar ionic resonance form (**5a′**) and one open-shell diradical form (**5a′′**) can be also drawn for **5a** (Fig. 1b). The existence of one more aromatic sextet ring (shaded in blue) in **5a′** and **5a′′** suggests that these two resonance forms might make a significant contribution to the ground state structure. Like all other pentacene analogues, bulky triisopropylsilylethynyl (in **5b**) or mesityl (in **5c**) groups are introduced to the reactive sites so that soluble and stable materials can be obtained. Detailed below are their synthesis, ground-state structures, physical properties and their applications for OTFTs. A comparison with pentacene and other pentacene analogues is also made to better understand the structure–property relationship.

## Results and discussion

### Synthesis and characterization

**Scheme 1:**
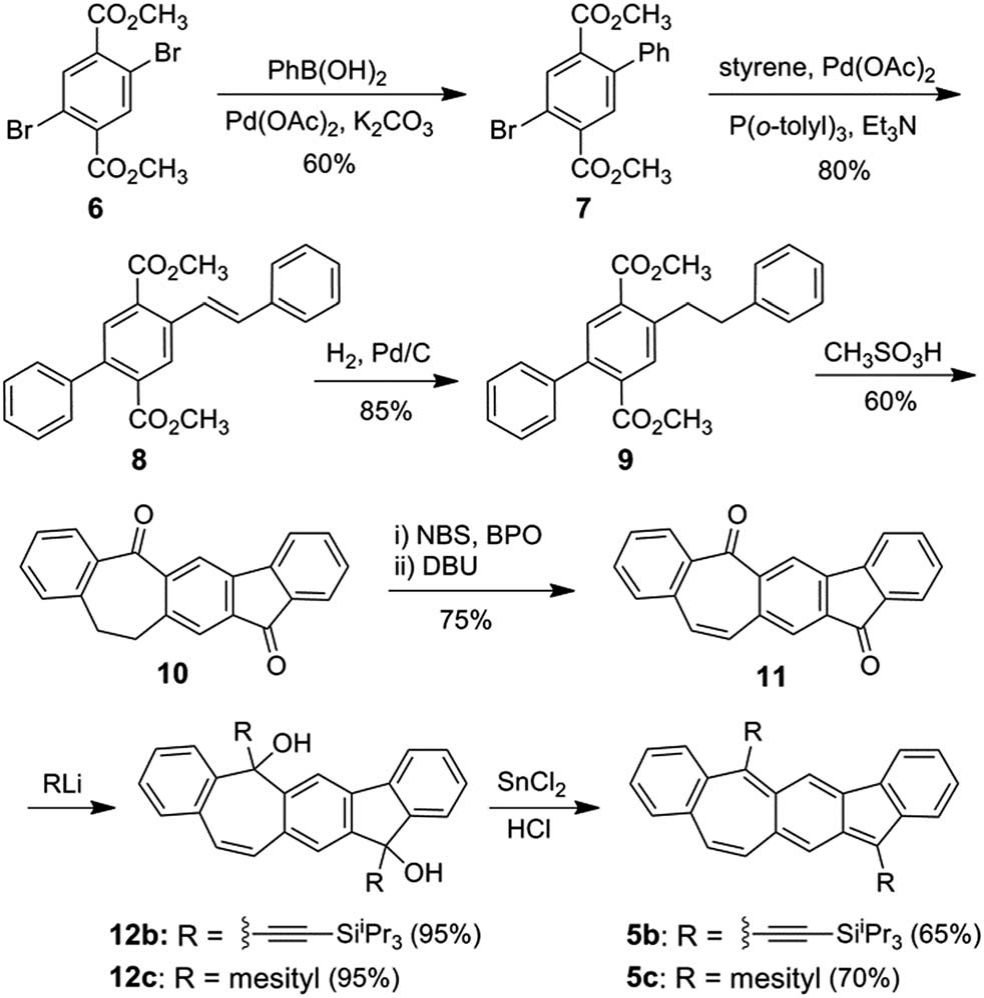
Synthesis of **5b**/**5c**.

Scheme 1 shows the synthesis of **5b** and **5c** starting from commercially available dimethyl 2,5-dibromoterephthalate **6**, which was coupled with phenyl boronic acid and styrene subsequently in the Suzuki reaction and Heck reaction, respectively, resulting in the diester **8**. Pd/C-catalyzed hydrogenation of **8** followed by treatment with methanesulfonic acid at 100 °C led to cyclized product **10**. Bromination of **10** and subsequent elimination of HBr yielded the dehydrogenated dione **11**. X-Ray crystallographic analysis of the single crystals of **11** revealed a non-planar geometry (Fig. S6 in ESI†), which can explain its moderate solubility in common organic solvents. Nucleophilic addition of (triisopropylsilyl)ethynyl and mesityl lithium to **11** resulted in the diols **12b** and **12c**, respectively, which both were obtained as a mixture of *cis* and *trans*-isomers. Reduction of intermediate diols **12b** and **12c** in THF with a solution of concentrated HCl that was saturated with SnCl_2_ led to **5b** and **5c**, respectively, both as deep green solids in moderate yield. Dione **10** was also synthesized from 2,5-dibromo-*p*-xylene in a similar approach in higher overall yield but more steps (Scheme S1 in ESI†). The ^1^H NMR spectra of **5b** and **5c** (ESI†) both show sharp splitting and narrow line widths indicating that they behave more like closed-shell compounds in the ground state.^16^

**Fig. 2:**
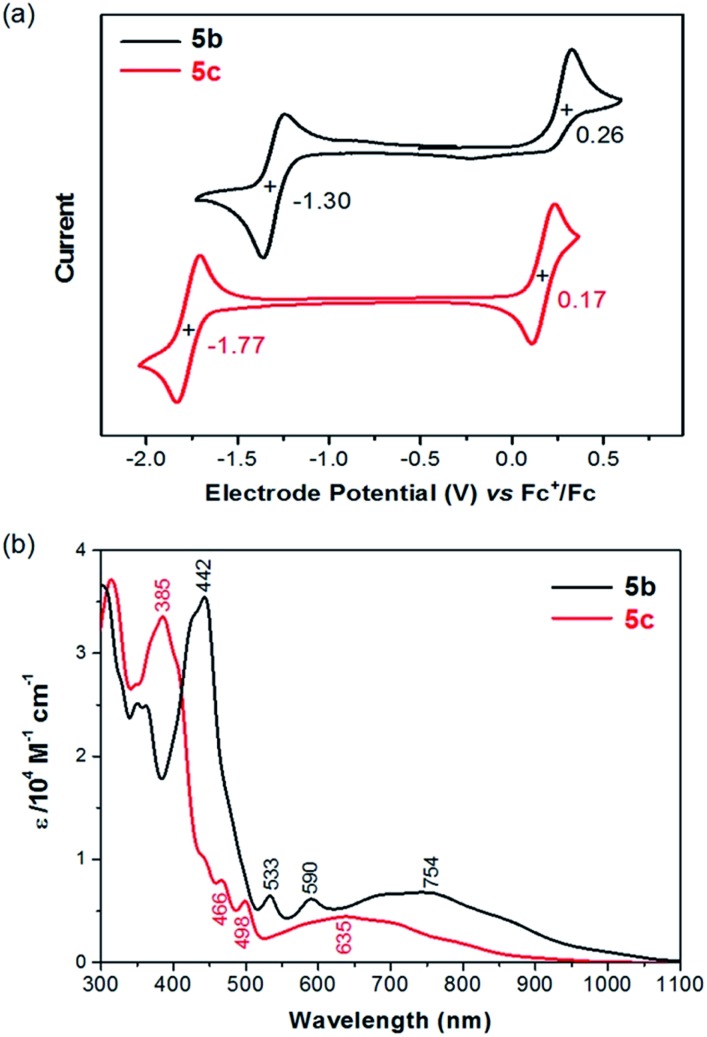
(a) Cyclic voltammograms of **5b** and **5c** recorded in CH_2_Cl_2_ with Fc^+^/Fc as the external standard at a scan rate of 50 mV s^–1^; (b) UV-vis-NIR absorption of **5b** and **5c** in CH_2_Cl_2_.

The redox behaviors of **5b**/**5c** in solution were investigated with cyclic voltammetry. In the test window of cyclic voltammetry, **5b** exhibits a reversible reduction (**5b**/**5b^–^**) wave and an irreversible oxidation (**5b**/**5b^+^**) wave, while **5c** exhibits a reversible reduction (**5c**/**5c^–^**) wave and a reversible oxidation (**5c**/**5c^+^**) wave as shown in Fig. 2a. The half-wave reduction potentials (*E*red1/2) of **5b** and **5c** are –1.30 V and –1.77 V *versus* the ferrocenium/ferrocene (Fc^+^/Fc) redox couple, respectively, from which the lowest unoccupied molecular orbital (LUMO) energy levels of **5b** and **5c** are estimated as –3.80 eV and –3.33 eV, respectively.^17^ Similarly, the highest occupied molecular orbital (HOMO) energy levels of **5b** and **5c** are estimated as –5.36 eV and –5.27 eV from the half-wave oxidation potential (*E*ox1/2 = 0.26 V and 0.17 V *vs.* Fc^+^/Fc, respectively).^17^ The lower LUMO and HOMO energy levels of **5b** in comparison with **5c** can be attributed to the facts that the ethynyl substituents with sp hybridized carbons in **5b** are electron withdrawing and the substituting phenyl groups in **5c** are almost orthogonal to the polycyclic backbone with poor conjugation. Table 1 compares **5b**/**5c** with those of the related molecules **1–4** in terms of electrochemical potentials and frontier molecular orbital energy levels. It is found that **5b** and **5c** have a higher HOMO energy level and a lower LUMO energy level than the corresponding pentacene derivatives **1b** and **1c**, respectively. Furthermore, the oxidation potential of **5b** is almost the same as that of **4b**, and the reduction potential of **5b** is close to that of **2b**. Molecule **5c** has a reduction potential close to that of **3c**, which has the same mesityl substituents. These findings are in agreement with the assumption that the first reduction of **5b**/**5c** occurs on the five-membered ring leading to an aromatic cyclopentadienide anion and the first oxidation of **5b**/**5c** occurs on the seven-membered ring leading to an aromatic cycloheptatrienium cation.

**Table 1:** Electrochemical potentials and frontier molecular orbital energy levels of **1–5**

	*E* red 1/2 ^*a*^/V	*E* ox 1/2 ^*a*^/V		LUMO^*b*^/eV	HOMO^*c*^/eV	*E* EC g ^*d*^/eV
**1b** 18	–1.50		0.37	–3.60	–5.47	1.87
**1c** 10	–1.92		0.22	–3.18	–5.32	2.14
**2b** 12	–1.15		0.74	–3.95	–5.84	1.89
**2c** 13	–1.58		0.64	–3.52	–5.74	2.22
**3c** 14	–1.13		0.13	–3.97	–5.23	1.26
**4b** 15	–1.66		0.12	–3.44	–5.32	1.78
**5b**	–1.30		0.26	–3.80	–5.36	1.56
**5c**	–1.77		0.17	–3.33	–5.27	1.94

^*a*^ *E* red 1/2 and *E*ox1/2 are the half-wave potential (*vs.* Fc^+^/Fc) of the first oxidation and reduction wave, respectively.

^*b*^Estimated from LUMO = –5.10 – *E*_red_ (eV).

^*c*^Estimated from HOMO = –5.10 – *E*_ox_ (eV).

^*d*^ *E* EC g = LUMO – HOMO.

As shown in Fig. 2b, **5b** and **5c** in CH_2_Cl_2_ exhibit electronic absorption spectra very different from those of pentacene and other analogues. The broad absorption band in the visible-near infrared (vis-NIR) region could be attributed to the HOMO → LUMO transition based on time-dependent density functional theory (TDDFT) calculations (ESI†). The intense absorption band at the UV-vis region can be mainly attributed to the HOMO–1 → LUMO and HOMO → LUMO+1 transitions. The optical energy gaps (*E*Optg) of **5b** and **5c** were estimated to be 1.13 eV and 1.25 eV, respectively, from the lowest energy absorption onset. The optical energy gap of **5b**/**5c** is significantly smaller than the HOMO–LUMO gap (*E*ECg) as estimated from electrochemical potentials. A similar phenomenon was also observed from azulene, which has an optical energy gap of 1.75 eV (about 710 nm)^19^ and an electrochemical energy gap of 2.35 eV.^20^ Azulene has a lower transition energy than anticipated from the HOMO–LUMO gap because the excited state of azulene has a smaller repulsive energy between the two electrons occupying HOMO and LUMO due to the nonalternant nature of azulene.^21^–^23^ This explanation may also account for the smaller optical energy gap of **5b**/**5c**, whose pentacyclic backbone is also nonalternant.

**Fig. 3:**
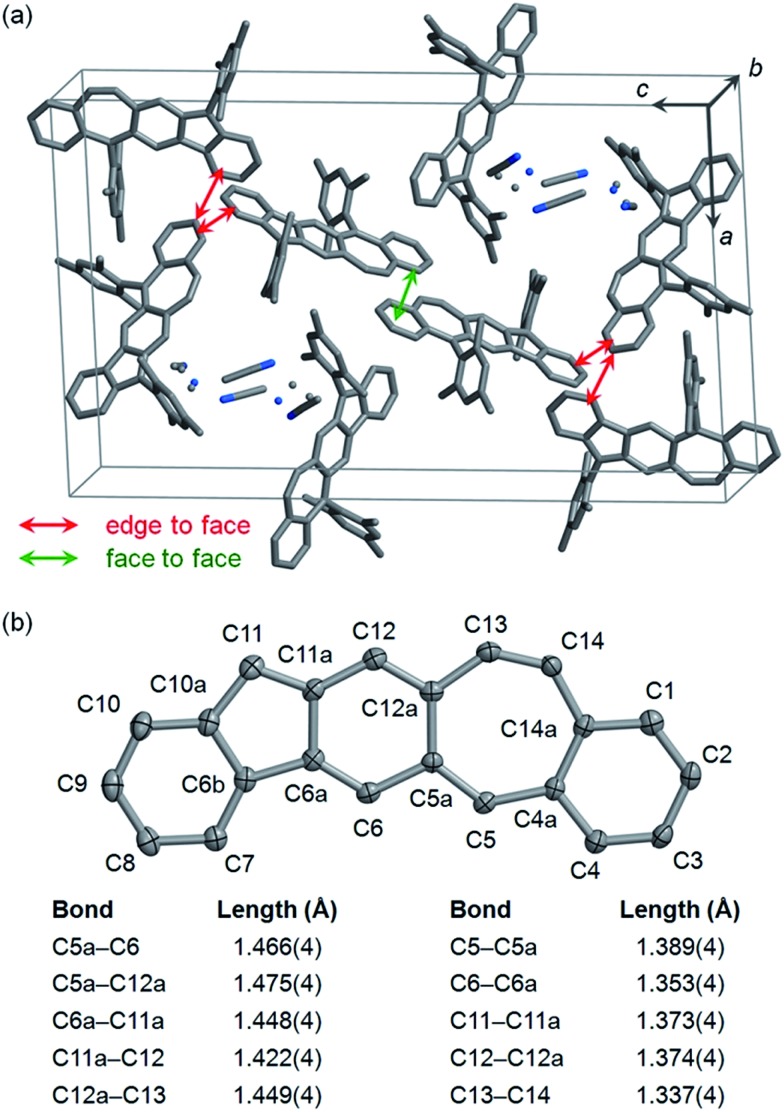
Crystallographic structure of **5c**·CH_3_CN with hydrogen atoms removed for clarification: (a) a unit cell with disordered atoms of CH_3_CN shown as dots; (b) the pentacyclic backbone of **5c** with carbon atoms labeled and some bond lengths highlighted (carbon atoms are shown as ellipsoids at the 50% probability level).

Single crystals of **5c** selected for X-ray crystallographic analysis were grown by slow diffusion of acetonitrile into a solution in CH_2_Cl_2_.^24^ It is found that the unit cell of this crystal contains crystallized solvent (CH_3_CN) molecules with disorder as shown in Fig. 3a. In the crystal structure of **5c**·CH_3_CN, the pentacyclic backbone of **5c** (Fig. 3b) is essentially flat and is almost perpendicular to the substituting mesityl groups with dihedral angles of 80.2° and 87.9°. Examination of the bond lengths in the central six-membered ring reveals four C–C single bonds (C5a–C12a, C5a–C6, C6a–C11a, C11a–C12) with bond lengths of 1.42–1.48 Å and two C–C double bonds (C6–C6a, C12–C12a) with bond lengths of 1.35–1.37 Å.^25^ Moreover, the central six-membered ring is bonded to C5 and C11 with relatively short bond lengths (C5–C5a: 1.39 Å; C11–C11a: 1.37 Å). The above bond lengths are similar to the corresponding bond lengths in the crystallographic structures of **2b**,^12^**2c**,^13^ and **4b**,^15^ indicating a *p*-quinodimethane structure with large bond length alternation. In addition to the C5–C5a bond, the seven-membered ring contains another C–C double bond (C13–C14) with a bond length (1.34 Å) typical for alkenes. Neighboring molecules of **5c** exhibit poor π–π interactions between the pentacyclic backbones presumably because the bulky mesityl substituting groups block π–π interactions. Only a small face-to-face overlap with a π-to-π distance of 3.40 Å and a small number of edge-to-face contacts are observed as shown in Fig. 3a.

### Computational studies

**Fig. 4:**
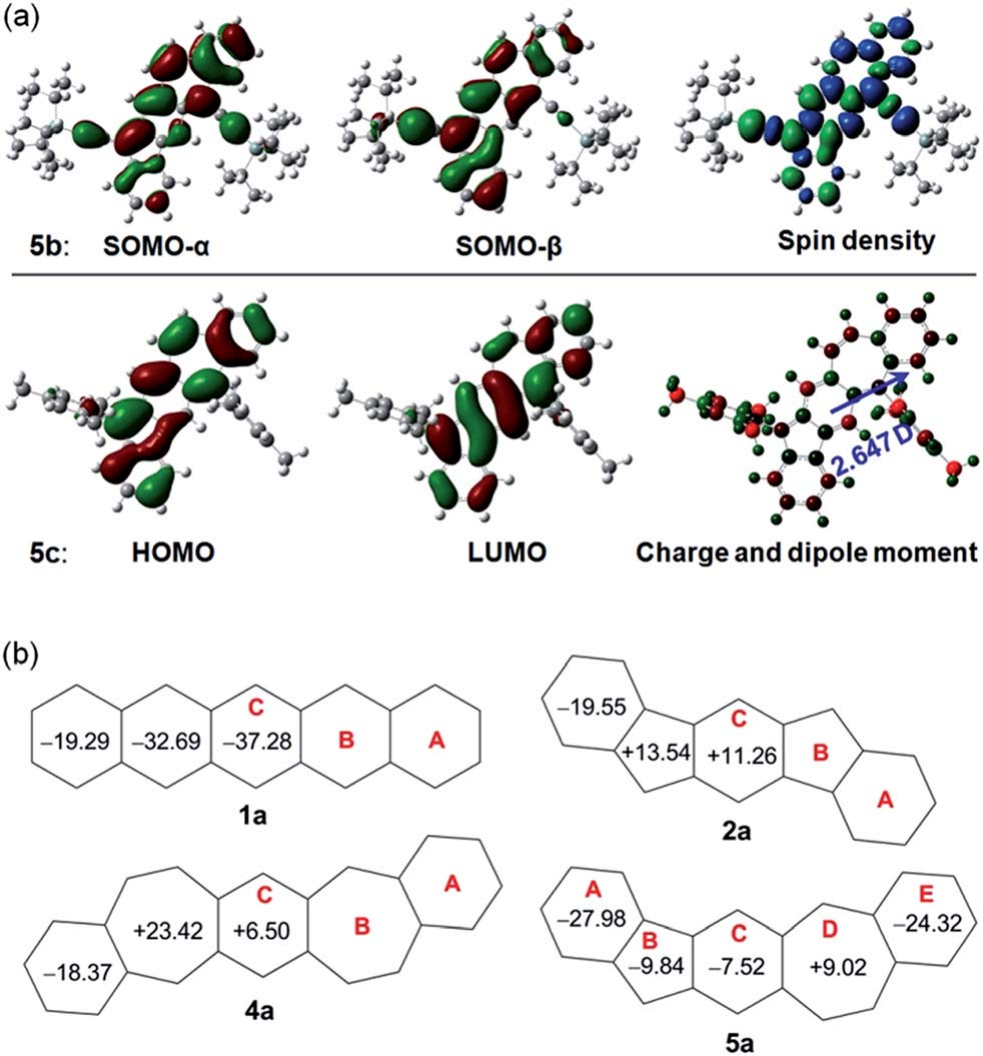
(a) Calculated frontier MO profiles of **5b** and **5c**, spin density map of singlet diradical of **5b**, and Mulliken charge distribution (–0.528 (red) to 0.528 (green)) and dipole moment of **5c**; (b) calculated NICS(1)zz values for pentacene and its analogues.

Density functional theory (DFT) calculations at the (U)CAM-B3LYP/6-31G* level of theory were conducted to better understand the ground state structures of **5a–c**. It is found that the solution of the open-shell singlet (OS) state has a lower energy than the closed-shell (CS) state for **5b**, thus defining an open-shell singlet ground state. The singly occupied molecular orbitals (SOMO) of the α and β spins are partially disjointed (Fig. 4a), in accordance with a calculated small diradical character (*y*_0_ = 4.7%). The spins are delocalized throughout the whole π-conjugated framework, including the C–C triple bonds (Fig. 4a). This result indicates that the diradical resonance form **5a′′** indeed contributes to the ground state of **5b** to a certain extent. On the other hand, **5a** and **5c** are calculated to have a closed-shell ground state with zero diradical character. The HOMO and LUMO of **5a** and **5c** are delocalized through the whole backbone with slight segregation as shown in Fig. S1 (ESI)† and 4a, respectively. The above results suggest that the ethynyl substituents can help to stabilize the diradical resonance form. The dipole moments of **5b** and **5c** were calculated to be 3.179 and 2.647 debye, respectively, at the CAM-B3LYP/6-31G* level of DFT, which are larger than that of azulene (1.268 debye) as calculated with the same method. This reflects the contribution of the dipolar ionic form **5a′** to the ground state of both **5b** and **5c**.

To provide further insight into the aromaticity of each individual ring of these π-conjugated polycyclic hydrocarbons, nucleus independent chemical shift (NICS) of **1a**, **2a**, **4a** and **5a** were also calculated. Fig. 4b compares the calculated NICS(1)zz values of these molecules. Large negative values are found for all rings in **1a**, in agreement with its known aromatic character. In **2a**, a large negative value is calculated for ring A while both ring B and ring C show positive values, indicating that it can be regarded as a dibenzo-fused anti-aromatic *s*-indacene structure. In **4a**, the central ring C is less positive compared with that in **2a**, indicating its less anti-aromatic character. The seven-membered ring B however has a large positive value. In **5a**, the central ring C and the five-membered ring B both become negative, and the seven-membered ring D is much less positive than that in **4a**, indicating that a balance of three resonance forms leads to a weak aromatic character of the central C_5_–C_6_–C_7_ framework. The outmost benzenoid rings (A and E) are aromatic with large negative values. In agreement with the negative NICS value for the central ring C in **5a**, the protons on the same ring in **5b** exhibit a downfield singlet peak at 8.95 ppm as well as a singlet peak 7.10 ppm in the ^1^H NMR spectrum. In comparison to this, the corresponding protons on the central ring C in **2b**^12^ and **4b**^15^ exhibit singlet peaks at 7.26 and 7.16 ppm, respectively, in the ^1^H NMR spectra taken from the same solution (CDCl_3_).

### Semiconductor properties

**Fig. 5:**
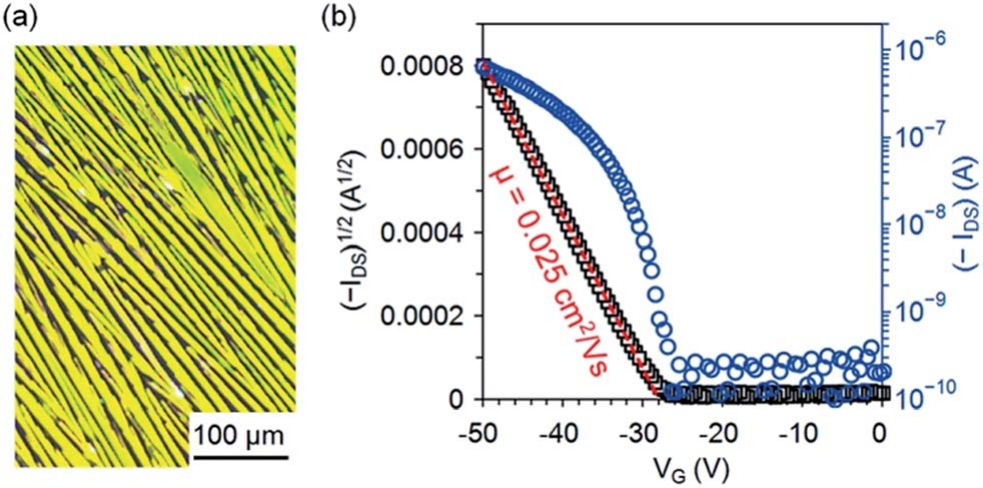
(a) Reflection polarized-light micrograph for a dip-coated film of **5b** on SiO_2_; (b) drain current (*I*_DS_) *versus* gate voltage (*V*_G_) with drain voltage (*V*_DS_) at –50 V for an OTFT of **5b** with an active channel of *W* = 1 mm and *L* = 100 μm as measured in air.

One interesting aspect of **5b** is its semiconducting properties since it is a constitutional isomer of pentacene **1b**, a well-known solution-processed p-type organic semiconductor. To test the semiconducting properties of **5b**, top-contact transistors were fabricated on dip-coated films of **5b**, which were formed by immersing a SiO_2_/Si substrate in a solution of **5b** (2.5 mg mL^–1^) in *n*-hexane and then pulling it up with a constant speed of 5.3 μm s^–1^. As shown in the polarized-light micrograph in Fig. 5a, the dip-coated films of **5b** on SiO_2_ are composed of crystalline fibers roughly aligned in the pulling direction; X-ray diffraction patterns from the films of **5b** (Fig. S4 in ESI†) exhibit an intense peak at *d*-spacing of 18.88 Å (2*θ* = 4.68°) accompanied with three higher-order peaks at 9.44 Å (2*θ* = 9.37°), 6.29 Å (2*θ* = 14.07°), and 4.72 Å (2*θ* = 18.80°), indicating a crystalline film with a layered structure. As measured in air from these devices, **5b** functions as a p-type semiconductor with a field-effect mobility of up to 0.025 cm^2^ V^–1^ s^–1^ (average 0.018 ± 0.003 cm^2^ V^–1^ s^–1^). Fig. 5b shows the transfer *I*–*V* curve in the saturation region for one of the best-performing OTFTs of **5b** measured in air. From this transfer *I*–*V* curve, the field mobility is extracted using the equation: *I*_DS_ = (*μWC*_i_/2*L*)(*V*_G_ – *V*_T_)^2^, where *I*_DS_ is the drain current, *μ* is field-effect mobility, *C*_i_ is the capacitance per unit area (11 nF cm^–2^) for the 300 nm-thick dielectric layer of SiO_2_, *W* is the channel width, *L* is the channel length, and *V*_G_ and *V*_T_ are the gate and threshold voltage, respectively. The mobility of **5b** is lower than those of **1b**^26^ and **4b**^15^ in solution-processed OTFTs on bare SiO_2_ by one order of magnitude likely because of the unsymmetrical arrangement of silylethynyl substituting groups, which presumably leads to unfavorable molecular packing with poor π–π interactions.

## Conclusions

In summary, the above study puts forth a new class of conjugated polycyclic molecules that contain a C_6_–C_5_–C_6_–C_7_–C_6_ framework isomeric to pentacene. The benzo[4,5]cyclohepta[1,2-*b*]fluorene derivatives **5b**/**5c** display different optical and electrochemical properties in comparison with pentacene and its analogues **2–4**. As found from the crystal structure, **5b** has a nearly flat pentacyclic π-backbone with a quinoidal core. The computational studies indicate that the dipolar ionic resonance form contributes to the ground states of both **5b** and **5c**, while the diradical characters in the ground state depends on the substituting groups. **5b** has a calculated diradical character (*y*_0_) in the ground state as small as 4.7%, which is not spectroscopically detectable, while **5c** has a closed-shell ground state with zero diradical character. As a constitutional isomer of pentacene **1b**, **5b** functions as a p-type organic semiconductor in solution-processed OTFTs with field effect mobility of up to 0.025 cm^2^ V^–1^ s^–1^. As an extension from this study, synthesis of novel polycyclic arenes containing both five- and seven membered rings is in progress in our laboratories. These molecules may exhibit interesting physical properties that are not available for their benzenoid analogues as suggested by a recent theoretical study.^27^

## Supplementary Material

Supplementary information

Crystal structure data
