# Supplementary material for: Benzo[4,5]cyclohepta[1,2-b]fluorene: an isomeric motif for pentacene containing linearly fused five-, six- and seven-membered rings
Source: Chem Sci. 2016 Jun 7;7(9):6176–81. doi: 10.1039/c6sc01795a (PMC6024176; doi:10.1039/c6sc01795a)
Supplement: Supplementary file 1 [file SC-007-C6SC01795A-s001.pdf]

Electronic Supplementary Material (ESI) for Chemical Science.
This journal is © The Royal Society of Chemistry 2016

Electronic Supplementary Information (ESI) for:

Benzo[4,5]cyclohepta[1,2-*b*]fluorene: An Isomeric Motif for Pentacene Containing Linearly Fused Five-, Six- and Seven-membered Rings

Table of Contents

1. Synthetic procedures and characterization data……………………………..…………..S2
2. DFT calculation details……………………………………………………………...S11
3. Fabrication and characterization of organic thin film transistors…………………….S15
4. Crystallographic data……………………………………………………..……S17
5. Appendix: NMR spectra of new compounds………………………………….......S19

1. Synthetic procedures and characterization data

General: The reagents and starting materials employed were commercially available and used without any further purification or prepared following reported methods as indicated. Anhydrous THF was purified by an Advanced Technology Pure-Solv PS-MD-4 system. ^{1}H and ^{13}C NMR spectra were recorded using Brucker ADVANCE III 400MHz and Advance 500 MHz spectrometer with tetramethylsilane (TMS) as the internal standard. The chemical shift was recorded in ppm and the following abbreviations were used to explain the multiplicities: s = singlet, d = doublet, t = triplet, m = multiplet, br = broad. Chemical shift values (δ) are expressed in parts per million using residual solvent protons (^{1}H NMR, δ_H = 7.26 for $CDCl_3$, δ_H = 2.50 for DMSO-d_6, δ_H = 7.16 for C_6D_6 and δ_H = 5.32 for CD_2Cl_2; ^{13}C NMR, δ_C = 77.16 for $CDCl_3$, δ_C = 39.52 for DMSO-d_6, δ_C = 53.84 for CD_2Cl_2 and δ_C = 128.06 for C_6D_6) as internal standard. Mass spectra were recorded on a Therno Finnigan MAT 95 XL spectrometer. X-ray crystallography data for **5c** were collected at 90 K with a Bruker APEX II X-Ray diffractometer equipped with a large area CCD detector by using graphite monochromated Mo-Kalpha radiation (lambda = 0.71073 Å). Melting points were measured using a Nikon Polarized Light Microscope ECLIPSE 50i POL equipped with an INTEC HCS302 heating stage. UV-vis-NIR absorption spectra were recorded on a Shimadzu UV-3600 spectrophotometer. Cyclic voltammetry were performed in dry dichloromethane on a CHI 620C electrochemical analyzer with a three-electrode cell, using 0.1 M Bu_4NPF_6 as supporting electrolyte, AgCl/Ag as reference electrode, gold disk as working electrode, Pt wire as counter electrode, and scan rate at 50 mV s^{-1}. The potential was externally calibrated against the ferrocene/ferrocenium couple.

Dimethyl 4-bromo-[1,1'-biphenyl]-2,5-dicarboxylate (7)

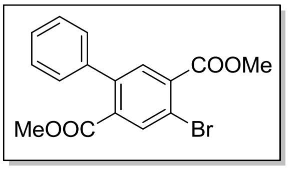

To a sealed tube containing phenylboronic acid (1.22 g, 10.0 mmol), dimethyl 2,5-dibromoterephthalate (**6**, 3.52 g, 10.0 mmol), palladium(II) acetate (112 mg, 0.499 mmol) and triphenylphosphine (289 mg, 1.10 mmol) were added degassed toluene (100 mL), ethanol (20 mL) and a 4 M K_2CO_3 aqueous solution (7.5 mL) successively under nitrogen atmosphere. The resulting mixture was stirred at room temperature for 20 minutes, and then stirred at 40 ℃ for 5 hours. After cooled to room temperature, the reaction mixture was quenched with 1 M HCl (30 mL) and extracted with ethyl acetate (3×). The combined organic layer was washed with saturated brine, dried over anhydrous Na_2SO_4 and concentrated under a reduced pressure. The crude product was purified by silica gel column chromatography using hexane/ ethyl acetate (v/v changed from 20/1 to 10/1) as eluent for gradient elution, yielding 2.09 g (60%) of dimethyl 4-bromo-[1,1'-biphenyl]-2,5-dicarboxylate (**7**) as colorless oil. ^{1}H NMR (400MHz, $CDCl_3$) δ (ppm): 8.09 (s, 1H), 7.79 (s, 1H), 7.38–7.43 (m, 3H), 7.28–7.30 (m, 2H), 3.94 (s, 3H), 3.66 (s, 3H). ^{13}C NMR (100MHz, $CDCl_3$) δ (ppm): 167.0, 165.9, 141.5, 139.2, 135.4, 134.4, 134.3, 133.4, 128.4, 128.2, 128.1, 120.2, 52.9, 52.5. HRMS (EI^+): calcd. for $C_{16}H_{13}BrO_4$ ($[M]^+$): 347.9992, found: 347.9992.

(*E*)-Dimethyl 4-styryl-[1,1'-biphenyl]-2,5-dicarboxylate (**8**)

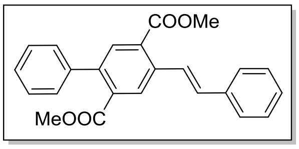

A mixture of dimethyl 4-bromo-[1,1'-biphenyl]-2,5-dicarboxylate (**7**, 4.20 g, 12.0 mmol), palladium(II) acetate (135 mg, 0.601 mmol) and tri-*o*-tolylphosphine (183 mg, 0.601 mmol) were dissolved in 35 mL of DMF in a sealed tube under a nitrogen atmosphere. Styrene (4.1 mL, 36.0 mmol) and 15 mL of triethylamine were added to the solution sequentially. The resulting mixture was stirred at room temperature for 20 minutes before put into an oil bath, which was preheated to 80 ℃. The reaction mixture was then gradually heated to 110 ℃ and stirred at this temperature for 4 hours. After cooled to room temperature, the mixture was treated with 40 mL of water, and extracted with ethyl acetate for five times. The organic layers were combined and washed with 6M HCl (three times), water (three times) and saturated brine successively. The solution was dried over anhydrous Na_2SO_4 and concentrated under a reduced pressure. The crude product was purified by silica gel column chromatography using hexane/ dichloromethane (v/v changed from 2/1 to 1/1) as eluent for gradient elution yielding 3.58 g (80%) (*E*)-dimethyl 4-styryl-[1,1'-biphenyl]-2,5 -dicarboxylate (**8**) as a white solid. mp: 146–148 ℃. ^1H NMR (400MHz, $CDCl_3$) δ (ppm): 8.15 (s, 1H), 8.01 (d, J = 16.4, 1H), 7.95 (s, 1H), 7.59 (d, J = 7.6, 2H), 7.28–7.44 (m, 8H), 7.17 (d, J = 16.4, 1H), 3.94 (s, 3H), 3.69 (s, 3H). ^{13}C NMR (100MHz, $CDCl_3$) δ (ppm): 168.7, 167.2, 140.7, 140.1, 138.1, 137.2, 134.2, 133.1, 132.8, 130.6, 128.9, 128.4, 128.3, 127.8, 127.1, 125.9, 52.6, 52.4. HRMS (EI^+): calcd. for $C_{24}H_{20}O_4$ ($[M]^+$): 372.1356, found: 372.1359 (error: +0.81 ppm).

Dimethyl 4-phenethyl-[1,1'-biphenyl]-2,5-dicarboxylate (**9**)

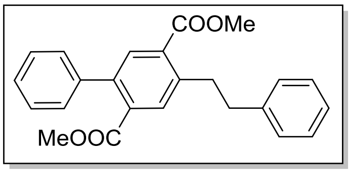

2.98 g (8.00 mmol) of (*E*)-dimethyl 4-styryl-[1,1'-biphenyl]-2,5- dicarboxylate (**8**) was dissolved in 80 mL of CH_2Cl_2. To the resulting solution were added 300 mg of 10% Pd/C and 60 mL of methanol. After H_2 gas was bubbled through the solution for 30 minutes, the reaction mixture was kept under an atmosphere of H_2 maintained by an inflated balloon with vigorous stirring at room temperature overnight. The mixture was filtered through celite, and the filtrate was concentrated under a reduced pressure. The crude product was purified by column chromatography on silica gel using hexane/CH_2Cl_2/EtOAc (20/5/1, v/v) as eluent for gradient elution yielding 2.55 g (85%) of dimethyl 4-phenethyl-[1,1'-biphenyl]-2,5 -dicarboxylate (**9**) as a white solid. mp: 126–127 ℃. ^1H NMR (400MHz, CD_2Cl_2) δ (ppm): 7.89 (s, 1H), 7.68 (s, 1H), 7.35–7.43 (m,3H), 7.25–7.32 (m, 6H), 7.19–7.22 (t, J = 7.0, 1H), 3.89 (s, 3H), 3.65 (s, 3H), 3.26–3.30 (m, 2H), 2.91–2.95 (m, 2H). ^{13}C NMR (100MHz, CD_2Cl_2) δ (ppm): 168.7, 167.5, 142.8, 142.2, 140.6, 140.2, 134.2, 133.2, 132.6, 132.4, 128.9, 128.8, 128.7, 128.5, 127.8, 126.4, 52.6, 52.4, 38.3, 36.6. HRMS (EI^+): calcd. for $C_{24}H_{22}O_4$ ($[M]^+$): 374.1513, found: 374.1511 (error: −0.53 ppm).

13,14-Dihydrobenzo[4,5]cyclohepta[1,2-*b*]fluorene-5,11-dione (10)

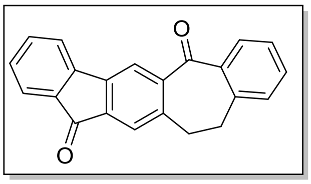

To a round-bottom flask containing 1.50 g (4.01 mmol) of dimethyl 4-phenethyl -[1,1'-biphenyl]-2,5-dicarboxylate (**9**) was added 50 mL of methanesulfonic acid. The resulting mixture was heated to 100 ℃ and then kept at this temperature for 24 hours with vigorous stirring. After cooled to room temperature, the mixture was poured into 100 mL of ice water with vigorous stirring, and the resulting suspension was extracted with ethyl acetate. The organic layers were combined and washed with water, $NaHCO_3$ aq. solution and saturated brine successively, dried with anhydrous Na_2SO_4, and concentrated under a reduced pressure. The crude product was purified by column chromatography on silica gel using hexane/CH_2Cl_2 (v/v changed from 1/1 to 1/3) as eluent for gradient elution, yielding 0.74 g (60%) of 13,14-dihydrobenzo[4,5]cyclohepta [1,2-*b*]fluorene-5,11-dione (**10**) as an orange solid. mp: 175–176 ℃. ^{1}H NMR (500MHz, $CDCl_3$, ppm): δ = 8.05-8.02 (m, 2H), 7.66 (d, *J* = 7.5 Hz, 1H), 7.57 (d, *J* = 7.0 Hz, 1H), 7.53-7.45 (m, 3H), 7.36 (t, *J* = 8.0 Hz, 1H), 7.30 (t, *J* = 7.80 Hz, 1H), 7.25 (d, *J* = 7.1 Hz, 1H), 3.25-3.20 (m, 4H); ^{13}C NMR (100MHz, $CDCl_3$) *δ* (ppm): 195.5, 193.4, 144.2, 144.0, 143.1, 142.21, 142.15, 137.7, 136.8, 135.3, 134.2, 133.0, 130.9, 129.8, 129.2, 126.9, 125.1, 124.5,121.9, 120.8, 34.8, 34.6. HRMS (EI$^+$): calcd. for $C_{22}H_{14}O_2$ ([M]$^+$): 310.0988, found: 310.0989 (error: +0.32 ppm).

Benzo[4,5]cyclohepta[1,2-*b*]fluorene-5,11-dione (11)

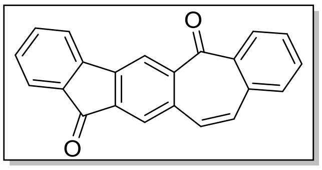

13,14-Dihydrobenzo[4,5]cyclohepta [1,2-*b*]fluorene-5,11-dione (**10**) (620 mg, 2.00 mmol), NBS (390 mg, 2.19 mmol) and dibenzoyl peroxide (20 mg) were dissolved in 50 mL of CCl_4, and the resulting solution was refluxed for 12 hours. After cooled to room temperature, the resulting mixture was filtered. The filtrate was concentrated under a reduced pressure. The residue was re-dissolved in 30 mL dry DMF. To the resulting solution was added 0.31 mL of 1,8-diazabicyclo[5.4.0]undec-7-ene (DBU). The mixture was heated to 80 ℃ and kept at this temperature with stirring for 6 hours. After cooled to room temperature, the mixture was poured into a 10% HCl aq. solution and extracted with a large amount of CH_2Cl_2. The organic layers were combined and washed with 10% HCl, dried over anhydrous Na_2SO_4, and concentrated under a reduced pressure. The residue was purified by silica gel column chromatography using hexane/ dichloromethane (v/v changed from 1/1 to 1/2) as eluent for gradient elution, yielding 463 mg (75%) of benzo[4,5]cyclohepta[1,2-*b*]fluorene-5,11-dione (**11**) as an orange solid. Recrystallization from toluene resulted in orange needles. mp: 295–297 ℃. ^{1}H NMR (400MHz, $CDCl_3$) *δ* (ppm): 8.33 (s, 1H), 8.22 (d, *J* = 7.6, 1H), 7.84 (s, 1H), 7.66–7.74 (m, 3H), 7.56–7.61 (m, 3H), 7.39 (t, *J* = 7.6, 1H), 7.15 (d, *J* = 12.4, 1H), 7.11 (d, *J* = 12.0, 1H). ^{13}C NMR (100MHz, $CDCl_3$) *δ* (ppm): 193.0, 192.9, 144.1, 143.24, 143.21, 138.4, 136.8, 136.3, 135.7, 135.1, 134.9, 132.62, 132.57, 131.2, 131.1, 130.4, 129.9, 129.5, 126.9, 124.9, 122.0, 121.4. HRMS (EI$^+$): calcd. for $C_{22}H_{12}O_2$ ([M]$^+$): 308.0832, found: 308.0831 (error: −0.32 ppm).

5,11-Bis((triethylsilyl)ethynyl)-5,11-dihydrobenzo[4,5]cyclohepta[1,2-*b*]fluorene -5,11-diol (12b)

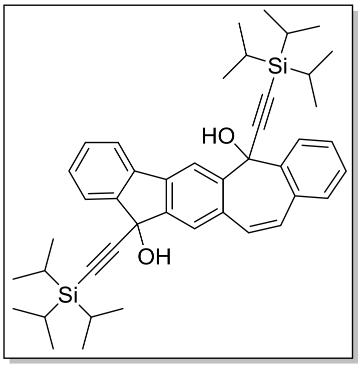

To a solution of triisopropylsilylacetylene (221 mg, 1.21 mmol) in 10 ml of anhydrous THF at 0 °C was added 0.78 mL of 1.6 M *n*-butyllithium in hexane (1.24 mmol) dropwise under a nitrogen atmosphere. The resulting mixture was stirred for 0.5 hour at room temperature. To the mixture was added 61.6 mg (0.200 mmol) of benzo[4,5]cyclohepta[1,2-*b*]fluorene -5,11-dione (**11**) under a nitrogen atmosphere. After being stirred for 5 hours, the reaction mixture was quenched with water and then extracted with diethyl ether for three times. The organic layers were combined, washed with saturated brine, dried with anhydrous Na_2SO_4, and concentrated under a reduced pressure. The resulting gel-like solid was purified by column chromatography on silica gel using hexane/diethyl ether (volume ratio changed from 10/1 to 3/1) as eluent for gradient elution yielding diol **12b** as two separated diastereomers.

Diastereomer I of **12b**: 44.2 mg, 33%. mp: 84–86 °C. ^{1}H NMR (400MHz, CD_2Cl_2) δ (ppm): 8.40 (s, 1H), 8.13 (d, J = 7.2, 1H), 7.65–7.69 (m, 3H), 7.41–7.46 (m, 3H), 7.32–7.37 (m, 2H), 7.16–7.23 (m, 2H), 3.19 (s, 1H), 2.74 (s, 1H), 1.10 (s, 21H), 1.03 (s, 21H). ^{13}C NMR (100MHz, CD_2Cl_2) δ (ppm): 148.2, 146.5, 142.2, 140.2, 139.8, 139.3, 133.8, 133.3, 132.1, 131.8, 130.1, 129.5, 129.1, 128.8, 127.7, 125.2, 124.6, 124.3, 120.8, 116.5, 108.4, 107.4, 85.5, 75.1, 18.9, 18.8, 11.7, 11.5. HRMS (EI$^+$): calcd. for $C_{44}H_{56}O_2Si_2$ ([M]$^+$): 672.3813, found: 672.3819 (error: +0.89 ppm).

Diastereomer II of **12b**: 84.1 mg, 62%. mp: 98–100 °C. ^{1}H NMR (400MHz, CD_2Cl_2) δ (ppm): 8.37 (s, 1H), 8.11 (d, J = 6.92, 1H), 7.66–7.67 (m, 3H), 7.40–7.47 (m, 3H), 7.32–7.37 (m, 2H), 7.16–7.21 (m, 2H), 3.22 (s, 1H), 2.70 (s, 1H), 1.07 (s, 21H), 1.04 (s, 21H). ^{13}C NMR (100MHz, CD_2Cl_2) δ (ppm): 148.1, 146.6, 142.4, 140.3, 139.7, 139.3, 133.8, 133.3, 132.1, 131.9, 130.1, 129.5, 129.1, 128.8, 127.6, 125.1, 124.6, 123.9, 120.8, 116.0, 108.4, 107.5, 85.3, 75.1, 18.83, 18.75, 11.68, 11.57. HRMS (EI$^+$): calcd. for $C_{44}H_{56}O_2Si_2$ ([M]$^+$): 672.3813, found: 672.3812 (error: −0.15 ppm).

5,11-Dimesityl-5,11-dihydrobenzo[4,5]cyclohepta[1,2-*b*]fluorene-5,11-diol (**12c**)

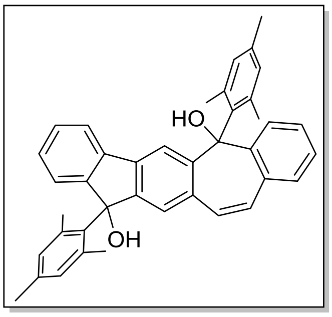

To a solution of 2-Bromomesitylene (241 mg, 1.21 mmol) in 10 ml of anhydrous THF at −78 ℃ was added 0.78 mL of 1.6 M *n*-butyllithium in hexane (1.24 mmol) dropwise under a nitrogen atmosphere, and the resulting mixture was stirred for 0.5 hour at −78 ℃. To the mixture was added 61.6 mg (0.200 mmol) of benzo[4,5]cyclohepta[1,2-*b*]fluorene-5,11-dione (**11**) under a nitrogen atmosphere, and the resulting mixture was warmed to room temperature. After being stirred for 5 hours at room temperature, the reaction mixture was quenched with water and then extracted with diethyl ether for three times. The organic layers were combined, washed with saturated brine, dried with anhydrous Na_2SO_4, and concentrated under a reduced pressure. The resulting gel-like solid was purified by column chromatography on silica gel using hexane/diethyl ether (v/v changed from 10/1 to 3/1) as eluent for gradient elution yielding the diol **12c** as two separated diastereomers.

Diastereomer I of **12c**: 73.5 mg, 67%. mp: 192–194 ℃. ^{1}H NMR (400MHz, CD_2Cl_2) δ (ppm): 8.56 (s, 1H), 8.24 (d, J = 8.0, 1H), 7.82 (d, J = 7.6, 1H), 7.44–7.48 (m, 1H), 7.41 (t, J = 7.2, 1H), 7.22–7.26 (m, 3H), 7.20 (d, J = 7.2, 1H), 7.14 (s, 1H), 6.98 (s, 1H), 6.74 (d, J = 11.2, 1H), 6.63–6.67 (m, 2H), 6.52 (d, J = 10.8, 2H), 2.88 (s, 3H), 2.84 (s, 1H), 2.26 (s, 3H), 2.10 (s, 3H), 2.08 (s, 1H), 1.51 (s, 3H), 1.39 (s, 3H), 1.36 (s, 3H). ^{13}C NMR (100MHz, CD_2Cl_2) δ (ppm): 148.5, 146.5, 145.0, 140.4, 140.0, 139.4, 138.0, 137.9, 137.6, 136.8, 136.5, 136.4, 135.5, 133.1, 132.7, 132.6, 131.73, 131.65, 131.1, 130.7, 130.5, 129.2, 129.0, 128.7, 128.0, 126.1, 124.2, 123.5, 121.1, 115.8, 86.4, 79.6, 25.9, 23.3, 22.5, 21.5, 20.6, 20.5. HRMS (EI$^+$): calcd. for $C_{40}H_{36}O_2$ ([M]$^+$): 548.2710, found: 548.2714 (error: +0.73 ppm).

Diastereomer II of **12c**: 30.7 mg, 28%. mp: 172–173 ℃. ^{1}H NMR (400MHz, CD_2Cl_2) δ (ppm): 8.55 (s, 1H), 8.25 (d, J = 8.4, 1H), 7.83 (d, J = 7.6, 1H), 7.45–7.49 (m, 1H), 7.38–7.42 (m, 1H), 7.23–7.25 (m, 4H), 7.14 (s 1H), 6.96 (s, 1H), 6.71 (d, J = 11.6, 1H), 6.65 (d, J = 11.2, 1H), 6.54 (d, J = 6.0, 2H), 6.49 (s, 1H), 2.94 (s, 3H), 2.83 (s, 1H), 2.34 (s, 1H), 2.18 (s, 3H), 2.12 (s, 3H), 1.52 (s, 3H), 1.34 (s, 3H), 1.00 (s, 3H). ^{13}C NMR (100MHz, CD_2Cl_2) δ (ppm): 148.8, 146.6, 145.0, 140.1, 140.0, 139.0, 138.12, 138.05, 137.8, 136.8, 136.5, 136.3, 135.5, 133.0, 132.7, 132.6, 131.7, 131.0, 130.6, 130.5, 129.3, 128.9, 128.6, 128.1, 126.1, 124.3, 123.7, 123.5, 121.1, 116.0, 86.4, 79.5, 25.9, 23.0, 22.4, 21.8, 20.53, 20.49. HRMS (EI$^+$): calcd. for $C_{40}H_{36}O_2$ ([M]$^+$): 548.2710, found: 548.2716 (error: 1.10 ppm).

5,11-Bis((triethylsilyl)ethynylbenzo[4,5]cyclohepta[1,2-*b*]fluorine (**5b**)

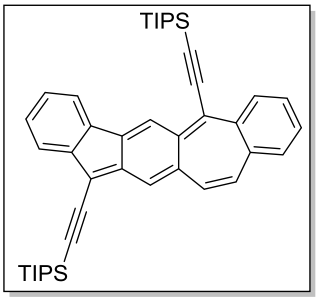

0.12 mL of acidic $SnCl_2$ saturated solution ($SnCl_2$ saturated in 37% HCl) was added to a solution of 67.3 mg (0.100 mmol) of diol **12b** (a mixture of two diastereomers) in 50 mL of THF at 0 ^{o}C. The immediately formed dark green solution was stirred at room temperature in dark for 30 minutes, and then diluted with 50 mL of water and extracted with diethyl ether. The organic layer was separated, washed with water, $NaHCO_3$ aq. solution and saturated brine successively, dried with anhydrous Na_2SO_4, and then concentrated under a reduced pressure. The crude product was purified by column chromatography on ***deactivated silica gel*** containing 5% water with CH_2Cl_2/hexane (1/100, v/v) as eluent in dark yielding 41.5 mg (65%) of 5,11-bis((triethylsilyl)ethynylbenzo[4,5]cyclohepta[1,2-*b*]fluorine (**5b**) as a dark green solid. mp: 141–143 ℃. ^{1}H NMR (400MHz, $CDCl_3$) δ (ppm): 8.95 (s, 1H), 8.64 (m, 1H), 7.82 (d, J = 7.4, 1H), 7.62 (d, J = 7.5, 1H), 7.47 (t, J = 7.5, 1H), 7.30–7.33 (m, 3H), 7.20–7.22 (m, 1H), 7.10 (s, 1H), 6.71 (d, J = 12.3, 1H), 6.48 (d, J = 2.3, 1H), 1.26–1.27 (m, 21H), 1.20 (s, 21H). ^{13}C NMR (100MHz, $CDCl_3$) δ (ppm): 143.5, 142.1, 141.0, 138.3, 137.3, 136.1, 135.9, 133.7, 133.5, 133.2, 131.9, 130.8, 130.3 130.1, 128.8, 128.1, 126.9, 125.3, 124.4, 120.8, 120.6, 114.4, 108.8, 108.5, 105.4, 102.8, 19.1, 19.0, 11.8, 11.6. HRMS (EI^+): calcd. for $C_{44}H_{54}Si_2$ ($[M]^+$): 638.3759, found: 638.3755 (error: −0.63 ppm)..

Preparation of ***deactivated silica gel***: 1.0 g of water was added to 20 g of silica gel suspended in hexane. The suspension was stirred until block solids disappeared. Then the suspension was used to fill a column for chromatography.

5,11-Dimesitylbenzo[4,5]cyclohepta[1,2-*b*]fluorene (**5c**)

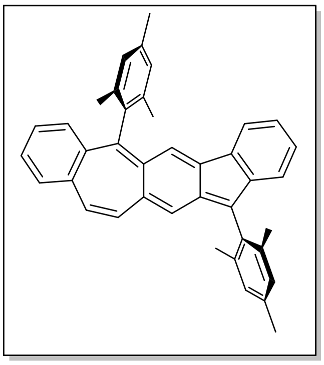

0.30 mL of acidic $SnCl_2$ saturated solution ($SnCl_2$ saturated in 37% HCl) was added to a solution of 54.9 mg (0.100 mmol) of diol **12c** (a mixture of two diastereomers) in 50 mL of THF at 0 ℃. The mixture was stirred at room temperature in dark for 10 hours. Then the resulting dark-green mixture was diluted with 50 mL of water and extracted with diethyl ether. The organic layer was separated, washed with water, $NaHCO_3$ aqueous solution and saturated brine successively, dried with anhydrous Na_2SO_4, and then concentrated under a reduced pressure. The crude product was purified by column chromatography on ***deactivated silica***

gel containing 5% water with CH_2Cl_2/hexane (1/40, v/v) as eluent in dark yielding 36.0 mg (70%) of 5,11-dimesitylbenzo[4,5]cyclohepta[1,2-*b*]fluorene (**5c**) as a dark green solid. mp: 260–261 ℃. ^{1}H NMR (400MHz, C_6D_6) δ (ppm): 7.74 (s, 1H), 7.57 (d, J = 7.4, 1H), 7.14 (s, 1H), 7.07 (d, J = 8.2, 2H), 6.83–6.96 (m, 8H), 6.66 (t, J = 8.0, 1H), 6.38 (d, J = 12.3, 1H), 6.20 (d, J = 12.2, 1H), 2.27 (s, 3H), 2.26 (s, 3H), 2.14 (s, 6H), 2.02 (s, 6H). ^{13}C NMR (100MHz, C_6D_6) δ (ppm): 147.7, 144.7, 140.9, 140.4, 139.1, 137.9, 137.8, 137.31, 137.29, 137.2, 136.8, 136.0, 134.1, 133.9, 133.2, 132.5, 132.2, 131.5, 130.0, 129.8, 129.1, 128.8, 128.6, 128.3, 128.1, 127.8, 124.6, 124.5, 124.4, 121.0, 120.4, 21.27, 21.25, 20.5, 19.9. HRMS (EI^+): calcd. for $C_{40}H_{34}$ ($[M]^+$): 514.2655, found: 514.2656 (error: +0.19 ppm).

Scheme S1 Alternative synthesis of diketone **10**.

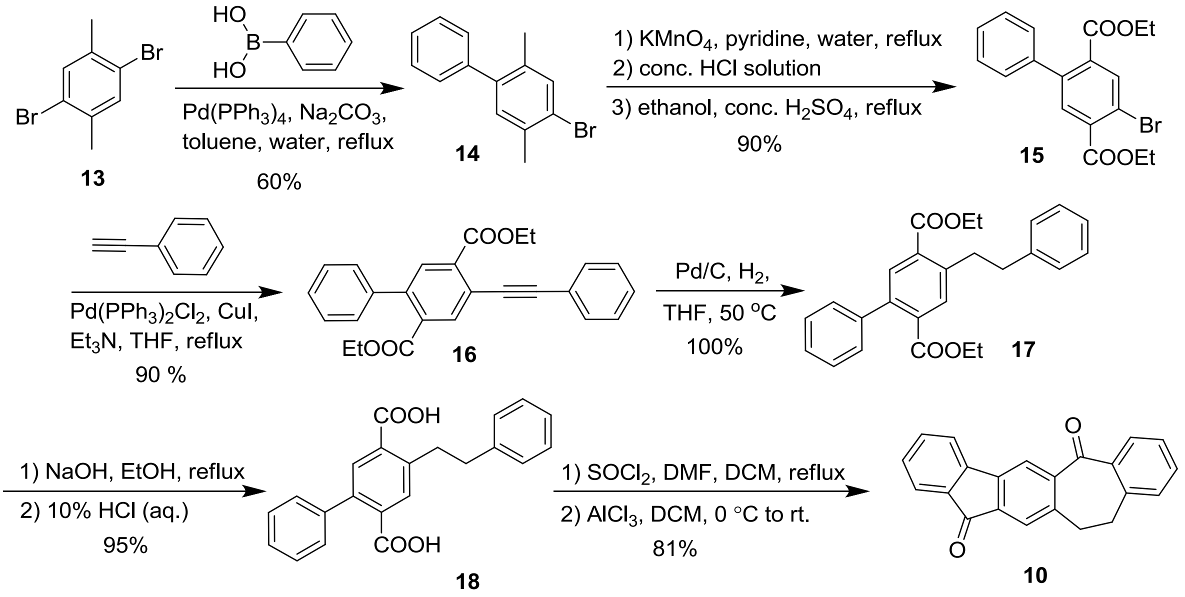

4-Bromo-2,5-dimethyl-1,1'-biphenyl (14)

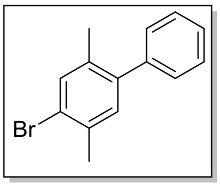

Phenyl boronic acid (2.43 g, 19.9 mmol), 2,5-dibromo-*p*-xylene (**13**, 16.0 g, 60.6 mmol), Na_2CO_3 (4.30 g, 40.6 mmol) and $Pd(PPh_3)_4$ (400 mg, 1.54 mmol) were dissolved in 100 mL of toluene and 50 mL of water (50 mL). After refluxing for 12 hours, the reaction mixture was cooled to room temperature and then poured into water and extracted with ethyl acetate. The organic layer was washed with water, dried over anhydrous Na_2SO_4, and concentrated under a reduced pressure. The residue was purified by column chromatography on silica gel using hexane as eluent, yielding 3.12 g (60%) of 4-bromo-2,5-dimethyl-1,1'-biphenyl (**14**) as colorless oil. ^{1}H NMR (500 MHz, $CDCl_3$, ppm): δ = 7.44 (s, 1H), 7.41 (t, J = 7.5 Hz, 2H), 7.32–7.37 (m, 1H), 7.28 (d, J = 7.9 Hz, 2H), 7.09 (s, 1H), 2.39 (s, 3H), 2.21 (s, 3H); ^{13}C NMR (125 MHz, $CDCl_3$, ppm): δ =141.08, 140.89, 134.93, 134.63, 133.68, 132.00, 128.97, 128.13, 126.98, 123.44, 22.24, 19.64. HR MS (EI): calcd for $C_{14}H_{13}Br$ (M^+), 260.0201; found, 260.0196 (error: −1.92 ppm).

Diethyl 4-bromo-[1,1'-biphenyl]-2,5-dicarboxylate (**15**)

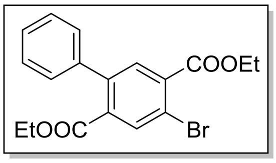

To a refluxing solution of 4-bromo-2,5-dimethyl-1,1'-biphenyl (**14**) (2.61 g, 10.0 mmol) in 20 mL of pyridine and 80 mL of water was slowly added 15.0 g of $KMnO_4$ in small portions over 3 hours. The reaction mixture was then refluxed overnight. After cooled to room temperature, the resulting mixture was filtered. The filtrate was acidified with concentrated HCl yielding 4-bromo-[1,1'-biphenyl]-2,5-dicarboxylic acid as a white solid, which was dried under vacuum and used without purification in next step.

The diacid as obtained above was dissolved in 30 mL of anhydrous ethanol. To the solution 5 mL of concentrated H_2SO_4 (5 mL) was slowly added, and the reaction mixture was refluxed overnight. After cooled to room temperature, the mixture was poured into water and extracted with ethyl acetate. The organic layer was washed by water, dried over anhydrous Na_2SO_4, and concentrated under a reduced pressure. The residue was purified by column chromatography on silica using hexane/ethyl acetate (15/1, v/v) as eluent yielding 3.40 g (90%) of diethyl 4-bromo-[1,1'-biphenyl]-2,5-dicarboxylate (**15**) as a waxy solid. ^{1}H NMR (500 MHz, $CDCl_3$, ppm): δ = 8.08 (s, 1H), 7.75 (s, 1H), 7.37–7.42 (m, 3H), 7.28–7.36 (m, 2H), 4.42 (q, *J* = 7.0 Hz, 2H), 4.11 (q, *J* = 7.3 Hz, 2H), 1.40 (t, *J* = 7.0 Hz, 3H), 1.01 (t, *J* = 7.1 Hz, 3H); ^{13}C NMR (125 MHz, $CDCl_3$, ppm): δ =166.55, 165.49, 141.31, 139.39, 135.13, 134.64, 134.59, 133.01, 128.19, 127.81, 119.95, 62.01, 61.55, 14.14, 13.57. HR MS (EI): calcd for $C_{18}H_{17}BrO_4$ (M^+), 376.0310; found, 376.0311 (error: +0.27 ppm).

Diethyl 4-(phenylethynyl)-[1,1'-biphenyl]-2,5-dicarboxylate (**16**)

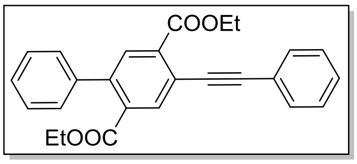

Diethyl 4-Bromo-[1,1'-biphenyl]-2,5-dicarboxylate (**15**) (1.13 g, 3.00 mmol), phenylacetylene (460 mg, 4.50 mmol), CuI (18.0 mg, 0.0945 mmol) and $Pd(PPh_3)_2Cl_2$ (30.2mg, 0.0430mmol) were dissolved in 30 mL of THF and 10 mL of triethylamine. The resulting solution was deoxygenated by freeze-thow-pump for three times and then slowly warmed to room temperature. After refluxing for 12 hours, the reaction mixture was cooled to room temperature, poured into an aqueous solution of 10% HCl, and extracted with ethyl acetate. The organic layer was washed with a 10% HCl aq. solution, dried over anhydrous Na_2SO_4, and concentrated under a reduced pressure. The residue was purified by column chromatography on silica gel using hexane/ethyl acetate (25/1, v/v) as eluent, yielding 1.08 g (90%) of diethyl 4-(phenylethynyl)-[1,1'-biphenyl]-2,5-dicarboxylate (**16**) as a white solid. ^{1}H NMR (500 MHz, $CDCl_3$, ppm): δ = 8.07 (s, 1H), 7.98 (s, 1H), 7.58–7.62 (m, 2H), 7.32–7.45 (m, 8H), 4.44 (q, *J* = 7.0 Hz, 2H), 4.13 (q, *J* = 7.2 Hz, 2H), 1.41 (t, *J* = 7.0 Hz, 3H), 1.04 (t, *J* = 7.0 Hz, 3H); ^{13}C NMR (125 MHz, $CDCl_3$, ppm): δ =167.44, 165.57, 141.45, 139.88, 135.20, 134.12, 133.92, 132.52, 131.74, 128.71, 128.39, 128.29, 128.19, 127.78, 123.04, 122.54, 95.40, 87.33, 61.58, 61.45, 14.31, 13.63. HR MS (EI): calcd for $C_{26}H_{22}O_4$ (M^+), 398.1518; found, 398.1501 (error: −4.27 ppm).

Diethyl 4-phenethyl-[1,1'-biphenyl]-2,5-dicarboxylate (17)

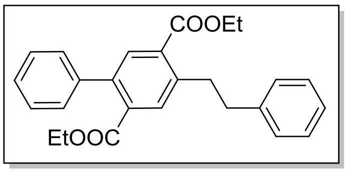

1.59 g (4.00 mmol) of 4-(phenylethynyl)-[1,1'-biphenyl]-2,5-dicarboxylate (**16**) was dissolved in 50 mL of dry THF. To the resulting solution was added 0.600 g of 10% Pd/C. The reaction mixture was kept at 50 ℃ under an atmosphere of H_2 maintained by an inflated balloon with stirring. Upon completion of the reaction as monitored by TLC (usually after 1 to 3 days), the reaction mixture was filtrated to remove the Pd/C. The filtrate was concentrated and dried under a reduced pressure, yielding 1.61 g (100%) of diethyl 4-phenethyl-[1,1'-biphenyl] -2,5-dicarboxylate (**17**) as a white solid. ^{1}H NMR (500 MHz, $CDCl_3$, ppm): δ = 7.89 (s, 1H), 7.68 (s, 1H), 7.36–7.43 (m, 3H), 7.27–7.35 (m, 6H), 7.20–7.24 (m, 1H), 4.39 (q, *J* = 7.0 Hz, 2H), 4.10 (q, *J* = 7.2 Hz, 2H), 3.33-3.31 (m, 2H), 2.98-2.95 (m, 2H), 1.39 (t, *J* = 7.2 Hz, 3H), 1.00 (t, *J* = 7.2 Hz, 3H); ^{13}C NMR (125 MHz, $CDCl_3$, ppm): δ =168.18, 166.82, 142.23, 141.61, 140.44, 139.88, 134.02, 132.61, 132.21, 132.18, 128.48, 128.38, 128.07, 127.36, 125.99, 61.26, 61.18, 37.96, 36.16, 14.28, 13.59. HR MS (EI): calcd for $C_{26}H_{26}O_4$ (M^+), 402.1831; found, 402.1836 (error: +1.24 ppm).

4-Phenethyl-[1,1'-biphenyl]-2,5-dicarboxylic acid (18)

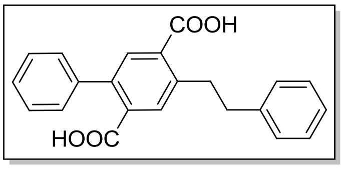

To a solution of diethyl 4-phenethyl-[1,1'-biphenyl] -2,5-dicarboxylate (**17**) (1.61 g, 4.00 mmol) in 60 mL of ethanol was added 2.0 g of NaOH. The resulting mixture was heated to reflux overnight. After cooled to room temperature, the reaction mixture was concentrated under a reduced pressure. Acidification of the residue with a 10% HCl aq. solution resulted in white precipitate, which was collected by filtration, washed with water and hexane, and dried under vacuum yielding 1.32 g (95%) of 4-phenethyl-[1,1'-biphenyl]-2,5 -dicarboxylic acid (**18**). ^{1}H NMR (500 MHz, DMSO-d_6, ppm): δ = 13.17 (br, 2H), 7.78 (s, 1H), 7.69 (s, 1H), 7.41–7.45 (m, 2H), 7.35–7.39 (m, 3H), 7.28–7.33 (m, 4H), 7.18–7.22 (m, 1H), 3.26-3.22 (m, 2H), 2.90-2.85 (m, 2H); ^{13}C NMR (125 MHz, DMSO-d_6, ppm): δ =169.12, 168.04, 141.65, 141.61, 139.80, 138.33, 134.99, 132.38, 132.08, 131.39, 128.31, 128.26, 128.22, 127.42, 125.93, 37.33, 35.53. HR MS (EI): calcd for $C_{22}H_{18}O_4$ (M^+), 346.1205; found, 346.1199 (error: −1.73 ppm).

13,14-Dihydrobenzo[4,5]cyclohepta[1,2-*b*]fluorene-5,11-dione (10)

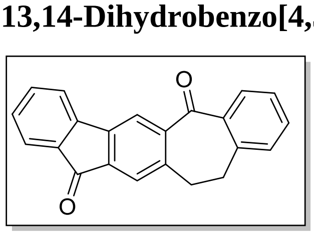

To 1.39 g (4.01 mmol) of 4-phenethyl-[1,1'-biphenyl]-2,5-dicarboxylic acid (**18**) was added 30 mL of anhydrous CH_2Cl_2, 3 mL of thionyl chloride, and 1 to 2 drops of anhydrous DMF successively. The resulting mixture was heated to reflux overnight. During this period the

insoluble diacid **18** was gradually dissolved in CH_2Cl_2. After cooled to room temperature, the resulting solution was concentrated under a reduced pressure to afford crude diacyl chloride, which was re-dissolved in 30 mL of anhydrous CH_2Cl_2. To the solution as cooled at 0 ℃ was carefully added 2.14 g (16.0 mmol) of anhydrous $AlCl_3$. The resulting mixture was allowed to warm up to room temperature and stirred overnight, then slowly quenched with a 10% HCl aqueous solution and extracted with CH_2Cl_2. The organic layer was washed with water, dried over anhydrous Na_2SO_4, and concentrated under a reduced pressure. The residue was purified by column chromatography on silica using hexane/ CH_2Cl_2 (1/1, v/v) as eluent, yielding 1.01 g (81%) of 13,14-dihydrobenzo[4,5]cyclohepta[1,2-*b*]fluorene-5,11-dione (**10**) as pale yellow solid. The ^{1}H and ^{13}C NMR as well HRMS of compound **10** prepared from **18** are essentially the same as those of compound **10** prepared from **9**.

2. DFT calculation details

Density functional theory calculations were employed with Gaussian 09 package,[1] utilizing the (U)CAM-B3LYP[2-4] level of theory with Pople basis set 6-31G*[5-7] for all molecules in the gas phase. NICS values at the (U)CAM-B3LYP/6-31G* level using the standard GIAO procedure (NMR pop=NCSall).[8] UV-vis-NIR absorption spectra of **5b** and **5c** in the closed-shell form were generated assuming an average UV-vis width of 4000 cm^{-1} at half-height using the SWizard program.[9]

References for DFT calculation:

1. *Gaussian 09; Revision A.2*; Frisch, M. J.; Trucks, G. W.; Schlegel, H. B.; Scuseria, G. E.; Robb, M. A.; Cheeseman, J. R.; Scalmani, G.; Barone, V.; Mennucci, B.; Petersson, G. A.; Nakatsuji, H.; Caricato, M.; Li, X.; Hratchian, H. P.; Izmaylov, A. F.; Bloino, J.; Zheng, G.; Sonnenberg, J. L.; Hada, M.; Ehara, M.; Toyota, K.; Fukuda, R.; Hasegawa, J.; Ishida, M.; Nakajima, T.; Honda, Y.; Kitao, O.; Nakai, H.; Vreven, T.; Montgomery, J., J. A.; Peralta, J. E.; Ogliaro, F.; Bearpark, M.; Heyd, J. J.; Brothers, E.; Kudin, K. N.; Staroverov, V. N.; Kobayashi, R.; Normand, J.; Raghavachari, K.; Rendell, A.; Burant, J. C.; Iyengar, S. S.; Tomasi, J.; Cossi, M.; Rega, N.; Millam, N. J.; Klene, M.; Knox, J. E.; Cross, J. B.; Bakken, V.; Adamo, C.; Jaramillo, J.; Gomperts, R.; Stratmann, R. E.; Yazyev, O.; Austin, A. J.; Cammi, R.; Pomelli, C.; Ochterski, J. W.; Martin, R. L.; Morokuma, K.; Zakrzewski, V. G.; Voth, G. A.; Salvador, P.; Dannenberg, J. J.; Dapprich, S.; Daniels, A. D.; Farkas, Ö.; Foresman, J. B.; Ortiz, J. V.; Cioslowski, J.; Fox, D. J.; Gaussian, Inc., Wallingford CT, **2009**.
2. Becke, A. D. *J. Chem. Phys.* **1993**, *98*, 5648.
3. Lee, C.; Yang, W.; Parr, R. G. *Phys. Rev. B: Condens. Matter* **1988**, *37*, 785.
4. Yanai, T.; Tew, D.; and Handy, N. *Chem. Phys. Lett.* **2004**, *393*, 51.
5. Ditchfie, R. W.; Hehre, J.; Pople, J. A. *J. Chem. Phys.* **1971**, *54*, 724.
6. Hehre, W. J.; Ditchfie R.; Pople, J. A. *J. Chem. Phys.* **1972**, *56*, 2257.
7. Harihara, P. C.; Pople, J. A. *Theor. Chim. Acta* **1973**, *28*, 213.
8. Fallah-Bagher-Shaidaei, H.; Wannere, S. S.; Corminboeuf, C.; Puchta, R.; Schleyer, P. V. R. *Org. Lett.* **2006**, *8*, 863.
9. Gorelsky, S. I., *SWizard program*, http://www.sg-chem.net/, University of Ottawa, Ottawa,Canada, **2010**.

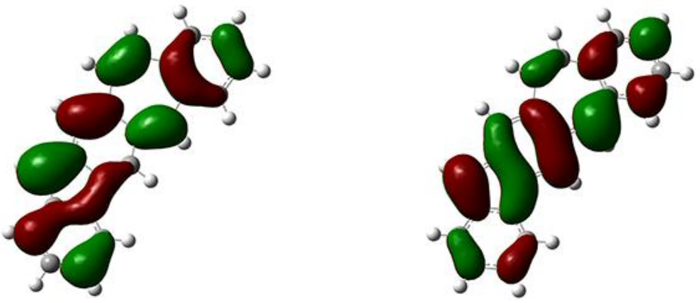

HOMO **LUMO**

Fig. S1. Calculated (CAM-B3LYP/6-31G*) frontier molecular orbital profiles of **5a**

Table S1. Calculated (CAM-B3LYP/6-31G*) energy levels of compound **5b** (closed-shell singlet).

	Hartree	eV
LUMO+5	0.07904	2.15
LUMO+4	0.0573	1.56
LUMO+3	0.04206	1.14
LUMO+2	0.03165	0.86
LUMO+1	-0.01519	-0.41
LUMO	-0.06672	-1.82
HOMO	-0.20938	-5.70
HOMO-1	-0.25847	-7.03
HOMO-2	-0.27342	-7.44
HOMO-3	-0.27801	-7.57
HOMO-4	-0.28238	-7.68
HOMO-5	-0.2831	-7.70
HOMO-6	-0.29046	-7.90
HOMO-7	-0.30052	-8.18
HOMO-8	-0.32665	-8.89
HOMO-9	-0.33673	-9.16

Table S2. Selected TD-DFT (CAM-B3LYP/6-31G*) calculated energies, oscillator strength and compositions of major electronic transitions of **5b**.

Wavelength (nm)	Osc. Strength (*f*)	Major contributions
617.4	0.3984	HOMO->LUMO (90%), H-1->LUMO (4%), HOMO->L+1 (4%)
372.6	1.1468	H-1->LUMO (42%), HOMO->L+1 (45%) HOMO->LUMO (8%)
276.37	0.3084	H-8->LUMO (11%), H-5->LUMO (24%), H-3->LUMO (22%), H-1->L+1 (25%)H-7->LUMO (4%), H-2->LUMO (3%)

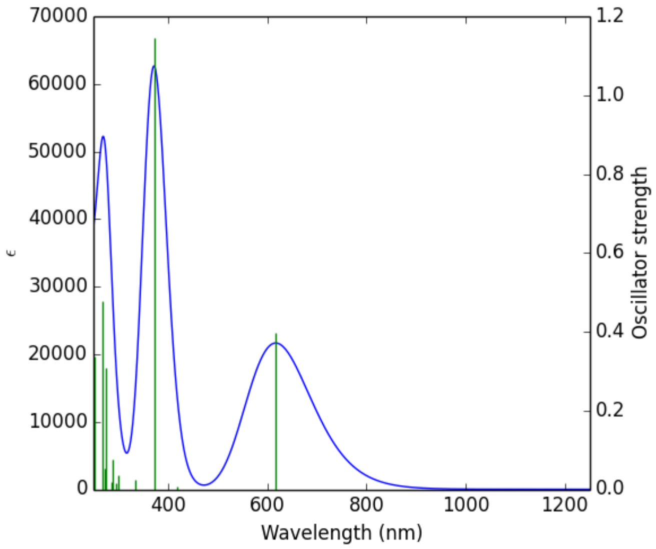

Fig. S2. Calculated (CAM-B3LYP/6-31G*) absorption spectrum for **5b**.

Table S3. Calculated (CAM-B3LYP/6-31G*) energy levels of compound **5c**.

	Hartree	eV
LUMO+5	0.05686	1.55
LUMO+4	0.05566	1.51
LUMO+3	0.04553	1.24
LUMO+2	0.03733	1.02
LUMO+1	-0.00933	-0.25
LUMO	-0.04667	-1.27
HOMO	-0.20938	-5.70
HOMO-1	-0.25847	-7.03
HOMO-2	-0.27342	-7.44
HOMO-3	-0.27801	-7.57
HOMO-4	-0.28238	-7.68
HOMO-5	-0.2831	-7.70
HOMO-6	-0.29046	-7.90
HOMO-7	-0.30052	-8.18
HOMO-8	-0.32665	-8.89
HOMO-9	-0.33673	-9.16

Table S4. Selected TD-DFT (B3LYP/6-31G*) calculated energies, oscillator strength and compositions of major electronic transitions of **5c**.

Wavelength (nm)	Osc. Strength (*f*)	Major contributions
548.9	0.2505	H-0->L+0(+88%) H-0->L+1(+6%)
336.5	1.1470	H-1->L+0(+64%) H-0->L+1(+23%) H-0->L+0(7%)

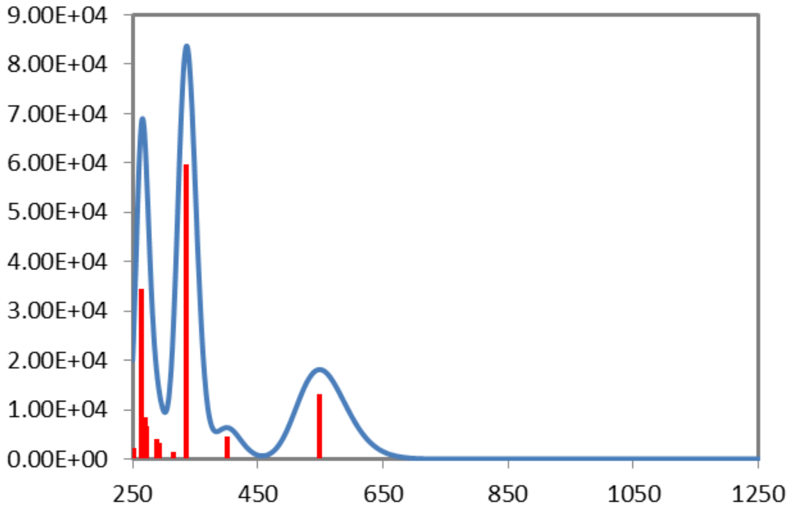

Fig. S3. Calculated (B3LYP/6-31G*) absorption spectrum for **5c**.

3. Fabrication and characterization of organic thin film transistors

(1) Dip coating thin films of **5b** and fabrication of OTFTs

The substrate used in the fabrication is a highly n-doped silicon wafer with a 300 nm-thick SiO_2 layer on the top. To form dip-coated films, a 1 cm × 0.5 cm silicon wafer was immersed vertically in a 2.5 mg/ml solution of **5b** in *n*-hexane and then was pulled up with a constant speed of 5.3 μm/s as controlled with a Longer Pump TJ-3A syringe pump controller.

To fabricate OTFTs, the solution-processed films were placed in a vacuum oven overnight to remove solvent residues. Then top contact drain and source gold electrodes were vacuum-deposited afterwards through a shadow mask onto the films by an Edward Auto 306 vacuum coating system with a Turbomolecular pump at a pressure of 4.0×10^{-6} torr or lower, with a deposition rate of ca. 2 nm/minute to a thickness about 30 nm as measured by a quartz crystal sensor, and the resulting semiconducting channels were 50 μm (L) × 1 mm (W), 100μm (L) × 1mm (W), 150 μm (L) × 1mm (W), 50 μm (L) × 2mm (W) and 100 μm (L) × 2mm (W).

(2) Characterization of the films and OTFTs.

Polarized optical images of the dip coated films were obtained using Nikon 50IPOL microscope. XRD measurement with θ-2θ scan mode was carried out on a SmartLab X-Ray Refractometer. The current-voltage measurement of thin-film transistors was conducted using a 4PROBES TECH ST-102C probe station and a Keithley 4200 Semiconductor Characterization System in air under ambient conditions.

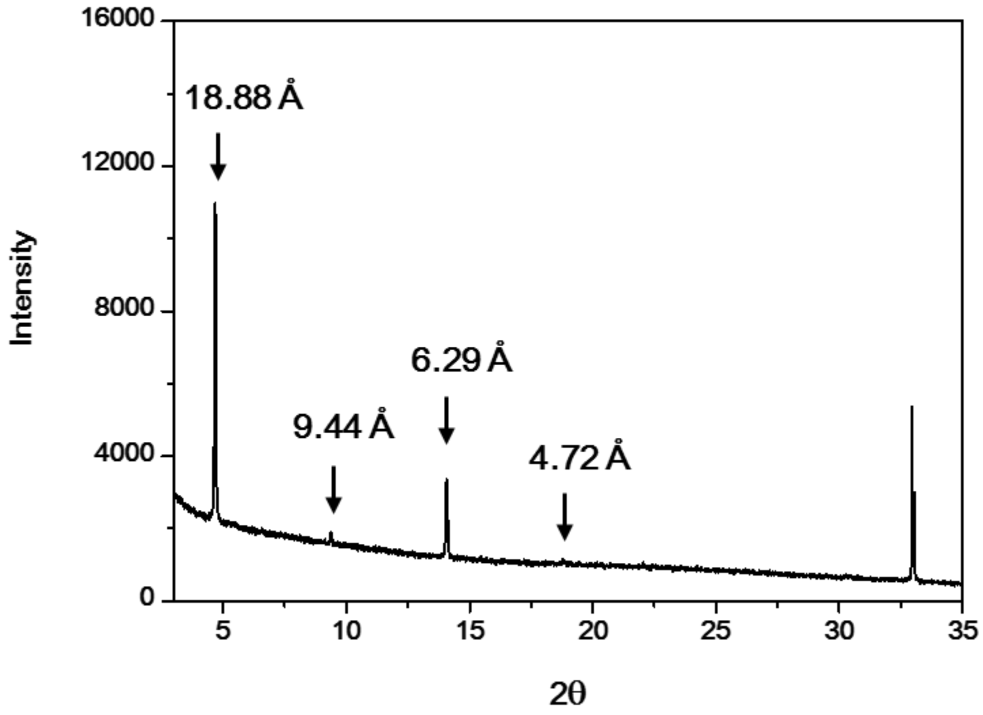

Fig. S4 X-ray diffractions from a dip-coated film of **5b** on SiO_2.

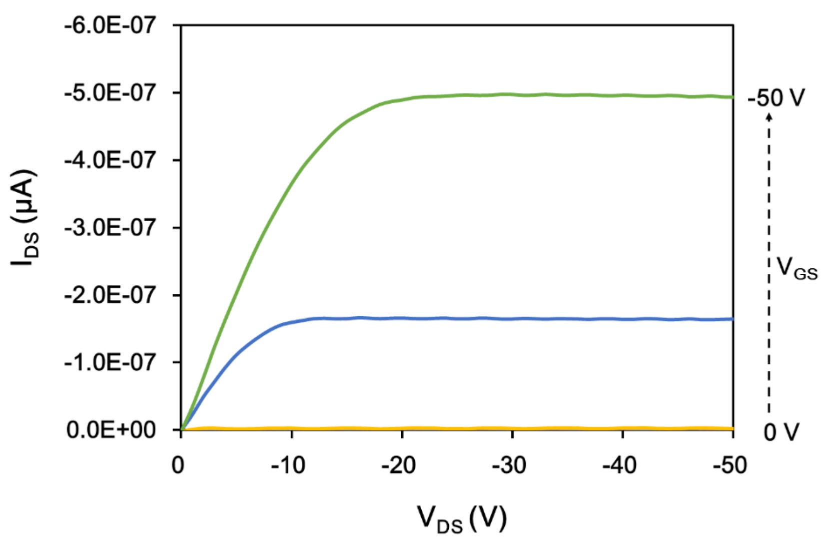

Fig. S5 (a) Drain current (I_{DS}) versus drain voltage (V_{DS}) with varying gated voltage (V_{GS}, from 0 to −50 V with a step of −10 V) for a dip coated OTFT of **5b** with channel dimension of W = 1 mm and L = 100 μm.

4. Crystallographic data

Table S5. Crystallographic data and structure refinement for **5c**.

Empirical formula	C42 H37 N	
Formula weight	555.72	
Temperature	90 K	
Wavelength	0.71073 Å	
Crystal system	Monoclinic	
Space group	$P2_1/n$	
Unit cell dimensions	a = 20.925(2) Å	α= 90 °.
	b = 8.4456(10) Å	β= 91.177(3) °.
	c = 34.677(4) Å	γ = 90 °.
Volume	6126.9(12) $Å^3$	
Z	8	
Density (calculated)	1.205 Mg/m^3	
Absorption coefficient	0.069 mm^{-1}	
F(000)	2368	
Crystal size	0.200 x 0.050 x 0.030 mm^3	
Theta range for data collection	1.126 to 24.984 °.	
Index ranges	-24<=h<=24, -10<=k<=9, -41<=l<=34	
Reflections collected	31171	
Independent reflections	10677 [R(int) = 0.0809]	
Completeness to theta = 24.984 °	99.4 %	
Absorption correction	Semi-empirical from equivalents	
Max. and min. transmission	0.998 and 0.887	
Refinement method	Full-matrix least-squares on F^2	
Data / restraints / parameters	10677 / 0 / 808	
Goodness-of-fit on F^2	1.030	
Final R indices [I>2sigma(I)]	R1 = 0.0652, wR2 = 0.1536	
R indices (all data)	R1 = 0.1248, wR2 = 0.1851	
Extinction coefficient	n/a	
Largest diff. peak and hole	0.392 and -0.592 e.$Å^{-3}$	

Table S6. Crystallographic data and structure refinement for **11** (CCDC number: 1468926).

Empirical formula	C22 H12 O2
Formula weight	308.32
Temperature	100(2) K
Wavelength	0.71073 Å
Crystal system	Monoclinic

Space group	Pc	
Unit cell dimensions	a = 13.9587(12) Å	α= 90°.
	b = 3.8076(3) Å	β= 111.997(3)°.
	c = 14.2557(12) Å	γ = 90°.
Volume	702.52(10) $Å^3$	
Z	2	
Density (calculated)	1.458 Mg/m^3	
Absorption coefficient	0.093 mm^{-1}	
F(000)	320	
Crystal size	0.486 x 0.192 x 0.076 mm^3	
Theta range for data collection	2.888 to 28.286°.	
Index ranges	-17<=h<=18, -4<=k<=5, -18<=l<=18	
Reflections collected	8274	
Independent reflections	3175 [R(int) = 0.0317]	
Completeness to theta = 25.242°	99.8 %	
Absorption correction	Semi-empirical from equivalents	
Max. and min. transmission	0.7457 and 0.6816	
Refinement method	Full-matrix least-squares on F^2	
Data / restraints / parameters	3175 / 2 / 217	
Goodness-of-fit on F^2	1.036	
Final R indices [I>2sigma(I)]	R1 = 0.0369, wR2 = 0.0853	
R indices (all data)	R1 = 0.0453, wR2 = 0.0895	
Absolute structure parameter	0.1(6)	
Extinction coefficient	n/a	
Largest diff. peak and hole	0.327 and -0.225 e.$Å^{-3}$	

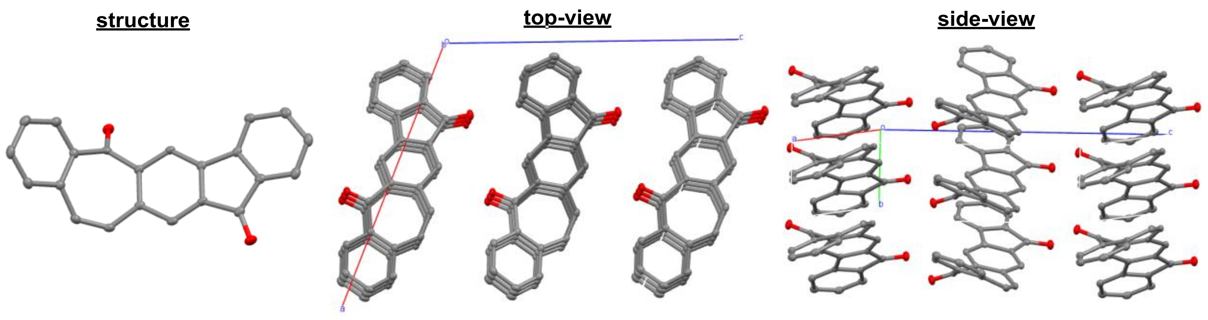

Fig. S6 Crystallographic structure and packing of **11**.

5. Appendix: NMR spectra of new compounds

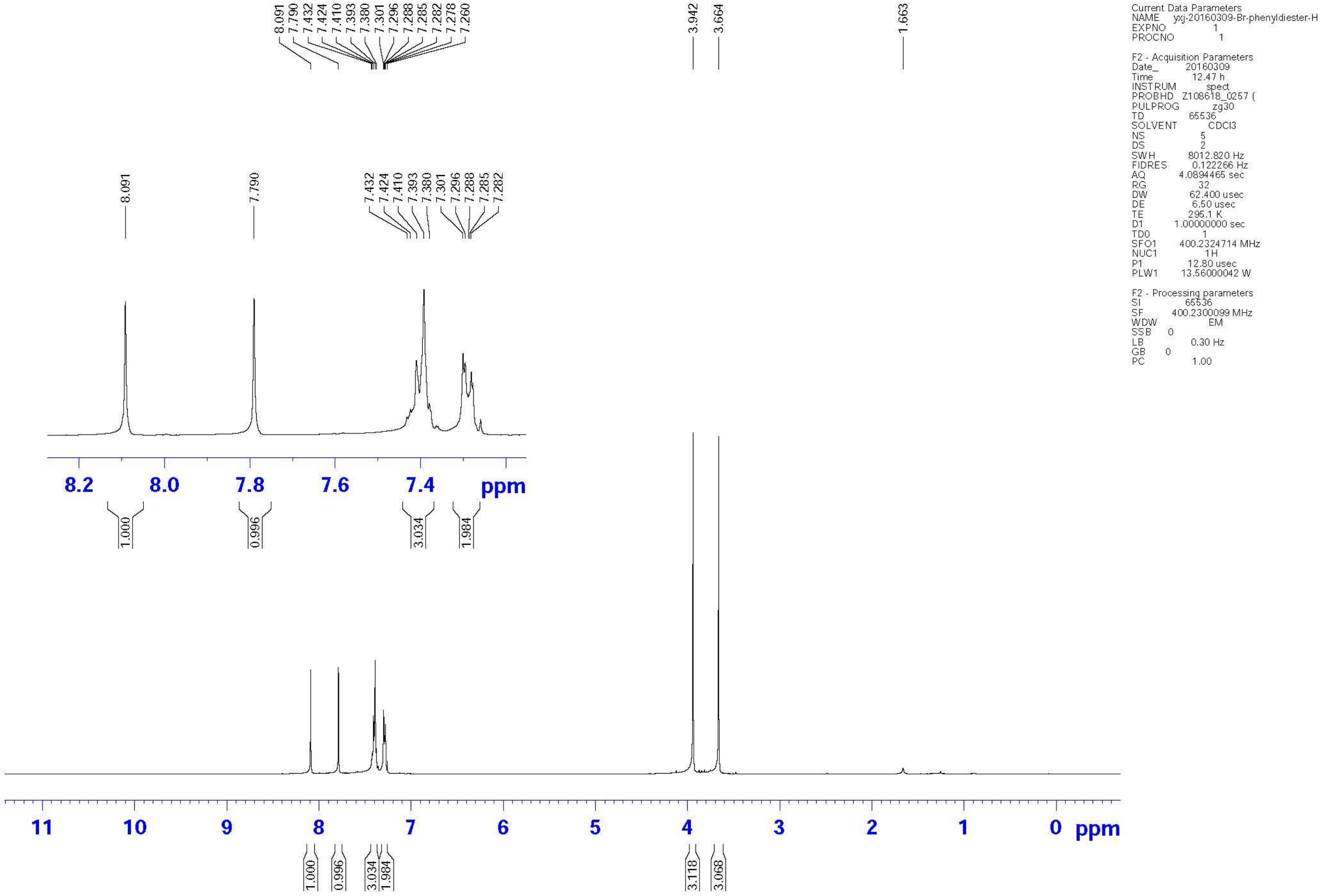

^{1}H NMR spectrum (400MHz, $CDCl_3$) of dimethyl 4-bromo-[1,1'-biphenyl]-2,5-dicarboxylate **(7)**

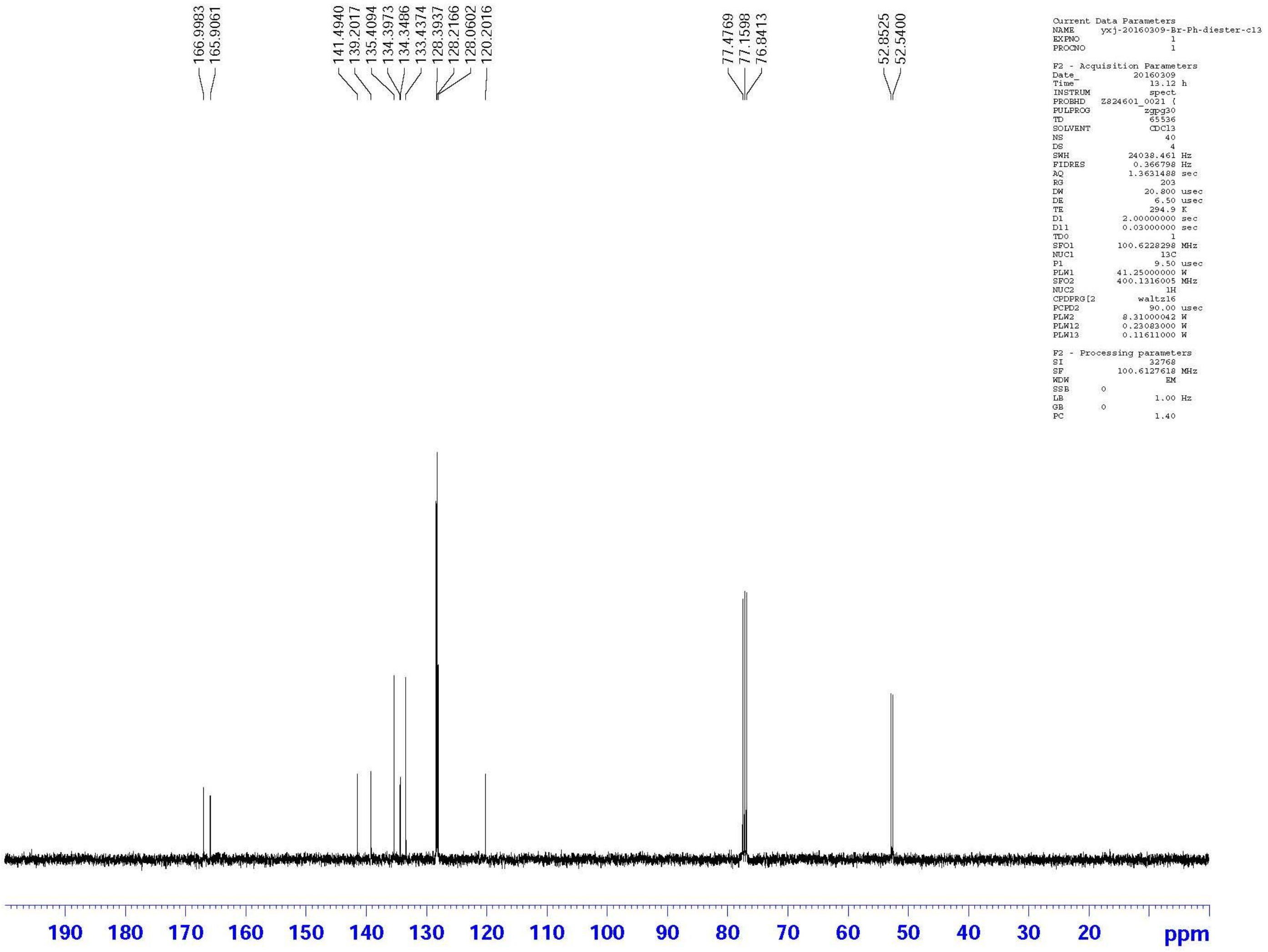

^{13}C NMR spectrum (100MHz, $CDCl_3$) of dimethyl 4-bromo-[1,1'-biphenyl]-2,5-dicarboxylate **(7)**

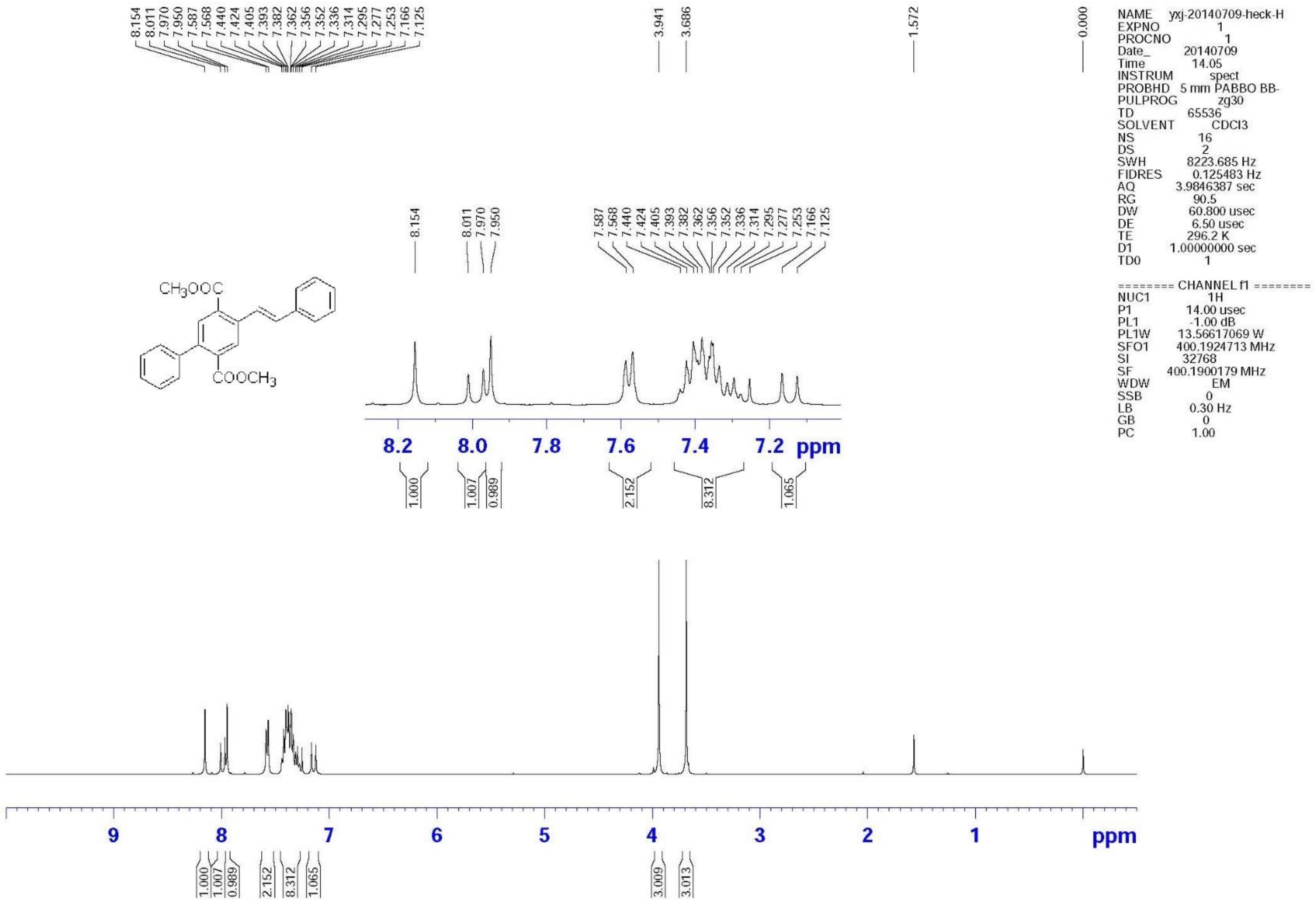

^{1}H NMR spectrum (400MHz, $CDCl_3$) of (E)-dimethyl 4-styryl-[1,1'-biphenyl]-2,5-dicarboxylate **(8)**

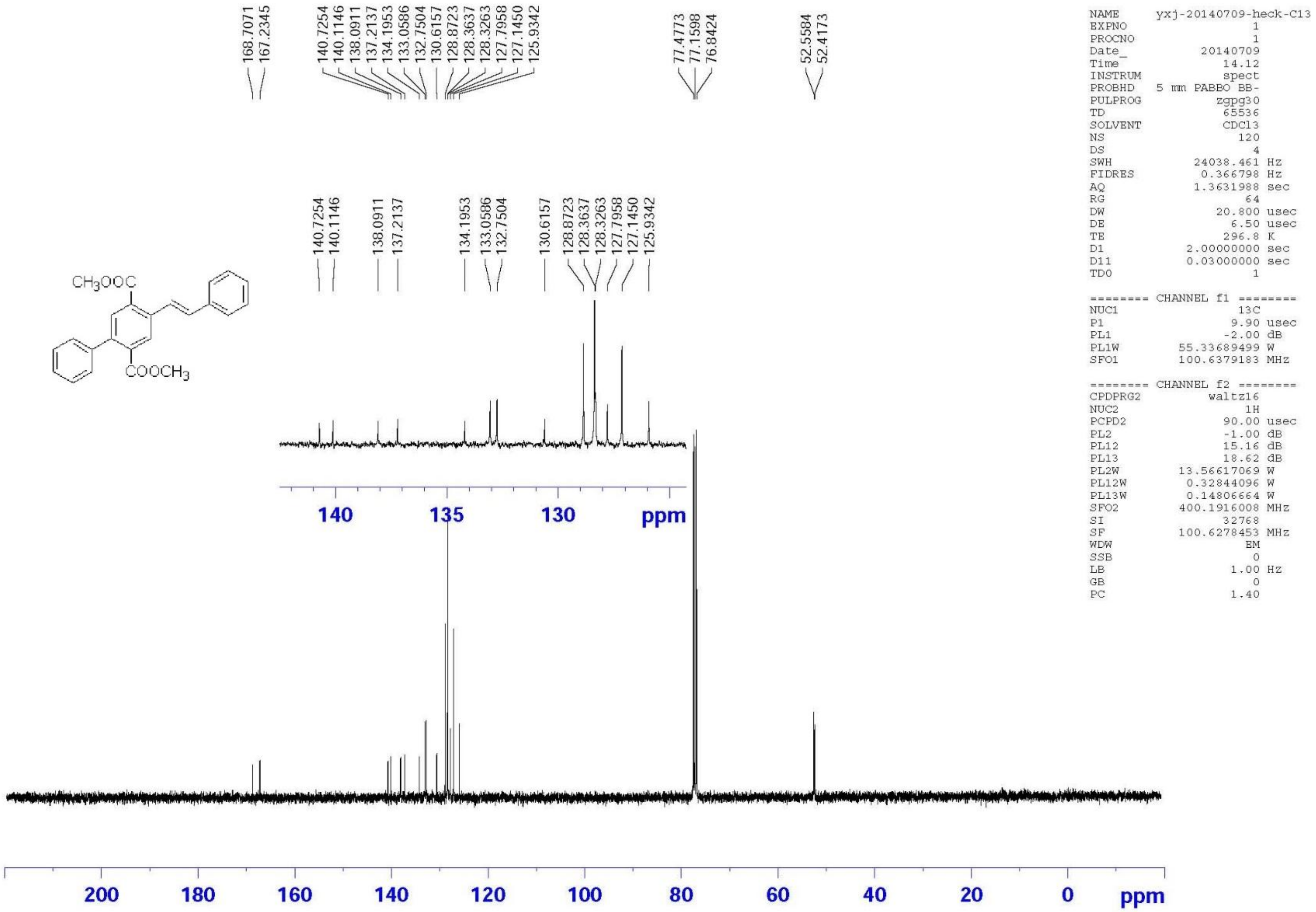

^{13}C NMR spectrum (100MHz, $CDCl_3$) of (E)-dimethyl 4-styryl-[1,1'-biphenyl]-2,5-dicarboxylate **(8)**

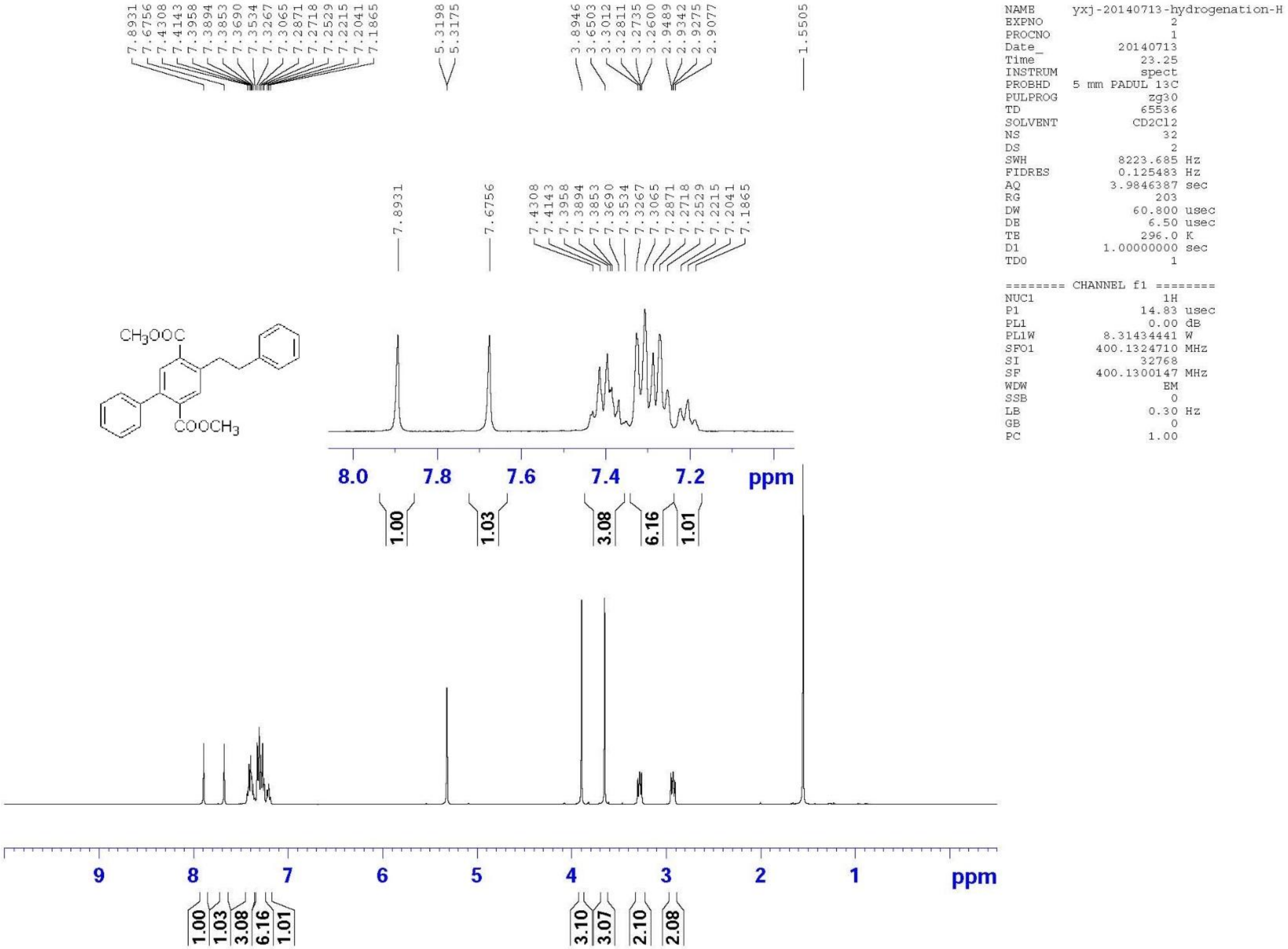

^{1}H NMR spectrum (400MHz, CD_2Cl_2) of dimethyl 4-phenethyl-[1,1'-biphenyl]-2,5-dicarboxylate **(9)**

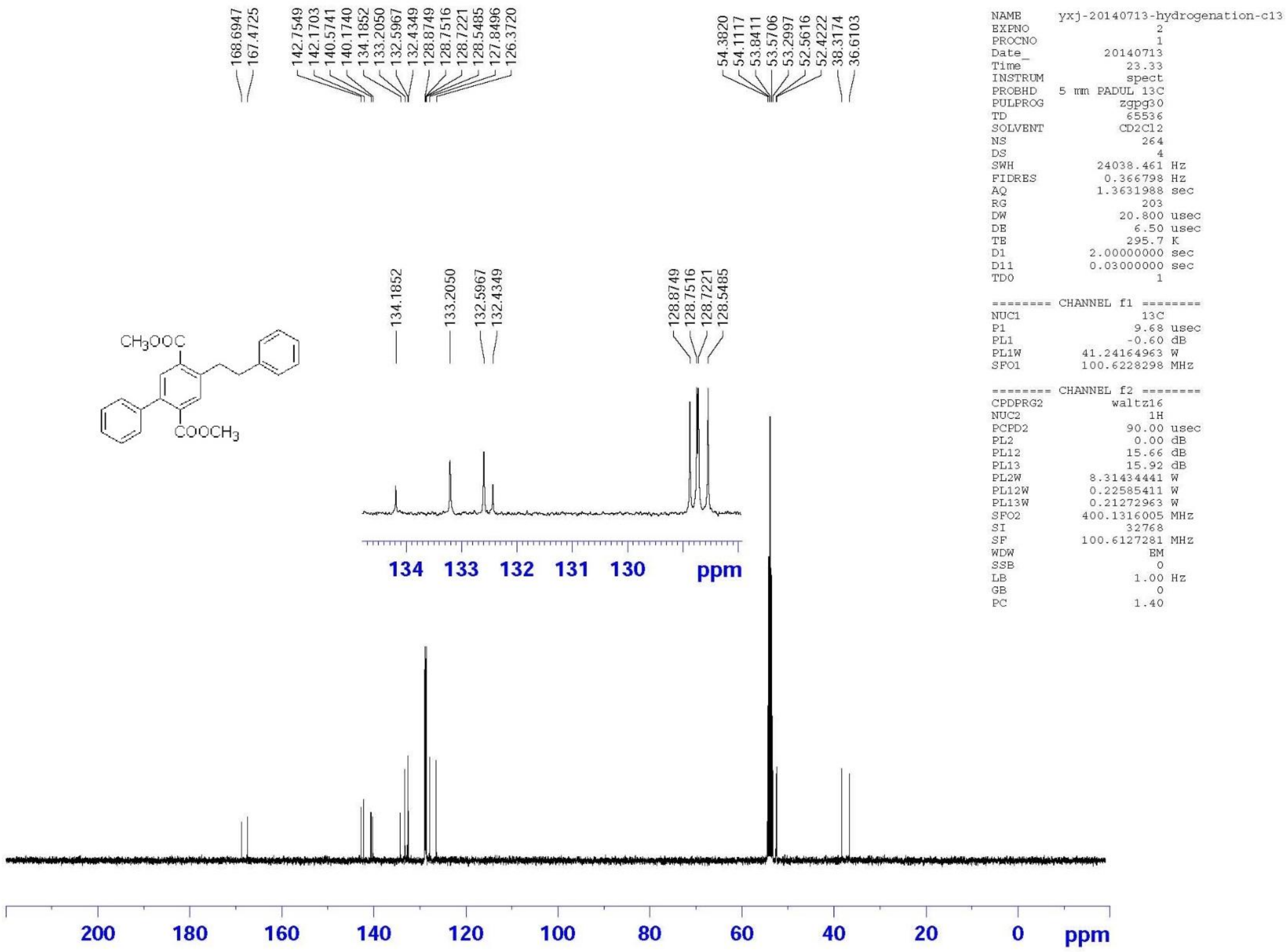

^{13}C NMR spectrum (100MHz, CD_2Cl_2) of dimethyl 4-phenethyl-[1,1'-biphenyl]-2,5-dicarboxylate **(9)**

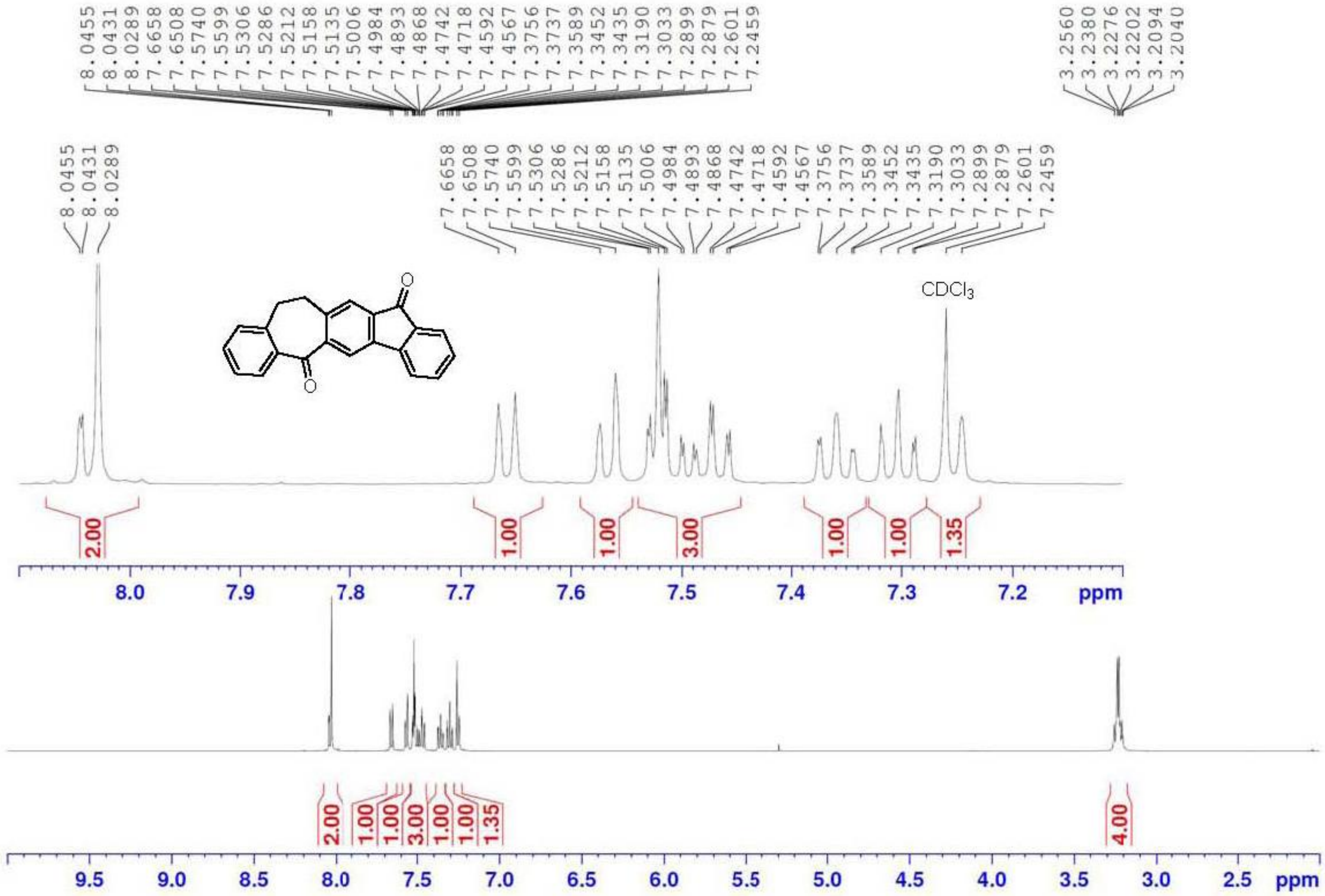

^{1}H NMR spectrum (500 MHz, $CDCl_3$) of 13,14-dihydrobenzo[4,5]cyclohepta[1,2-*b*]fluorene-5,11-dione **(10)**

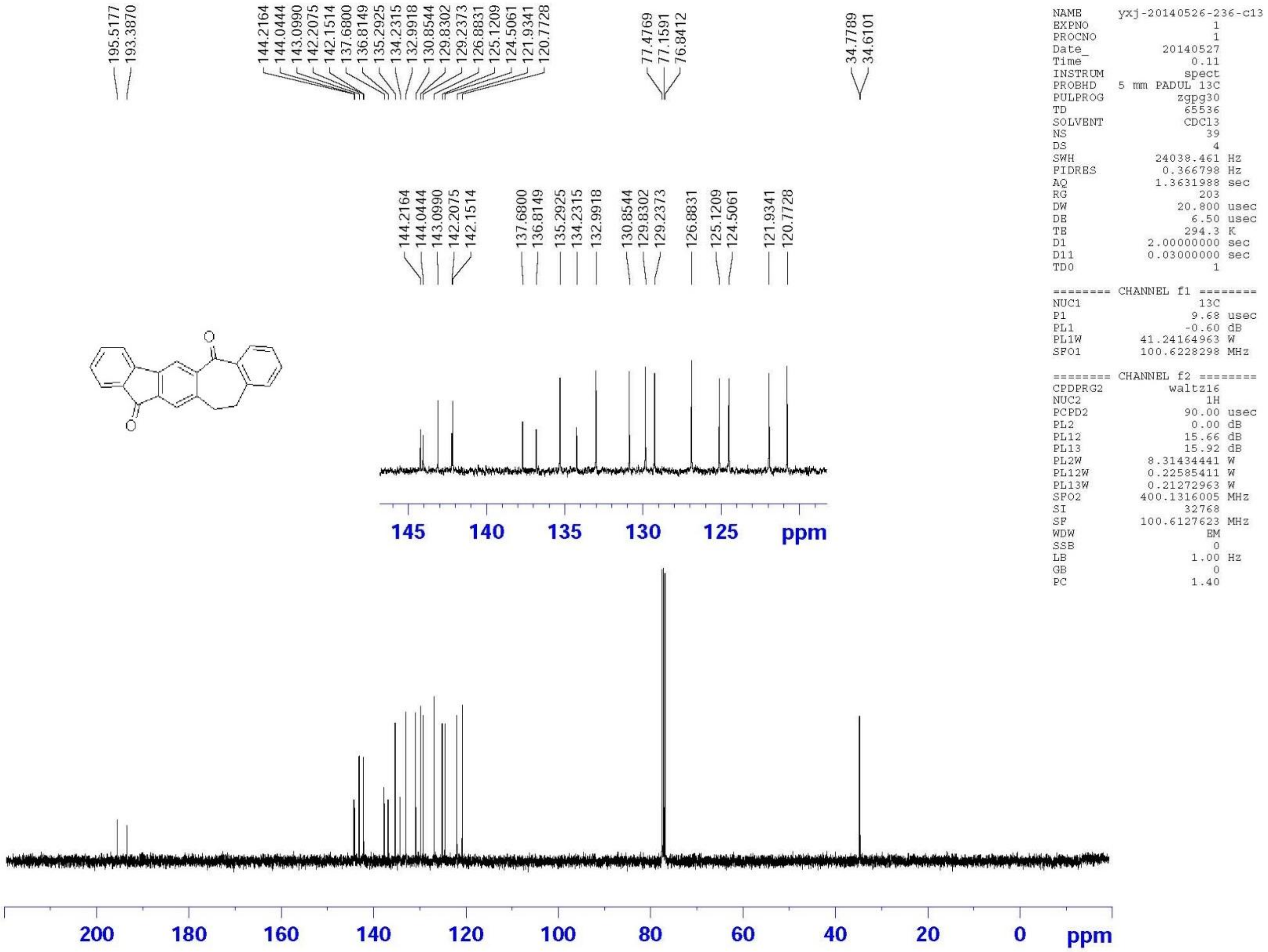

^{13}C NMR spectrum (100MHz, $CDCl_3$) of 13,14-dihydrobenzo[4,5]cyclohepta[1,2-*b*]fluorene-5,11-dione **(10)**

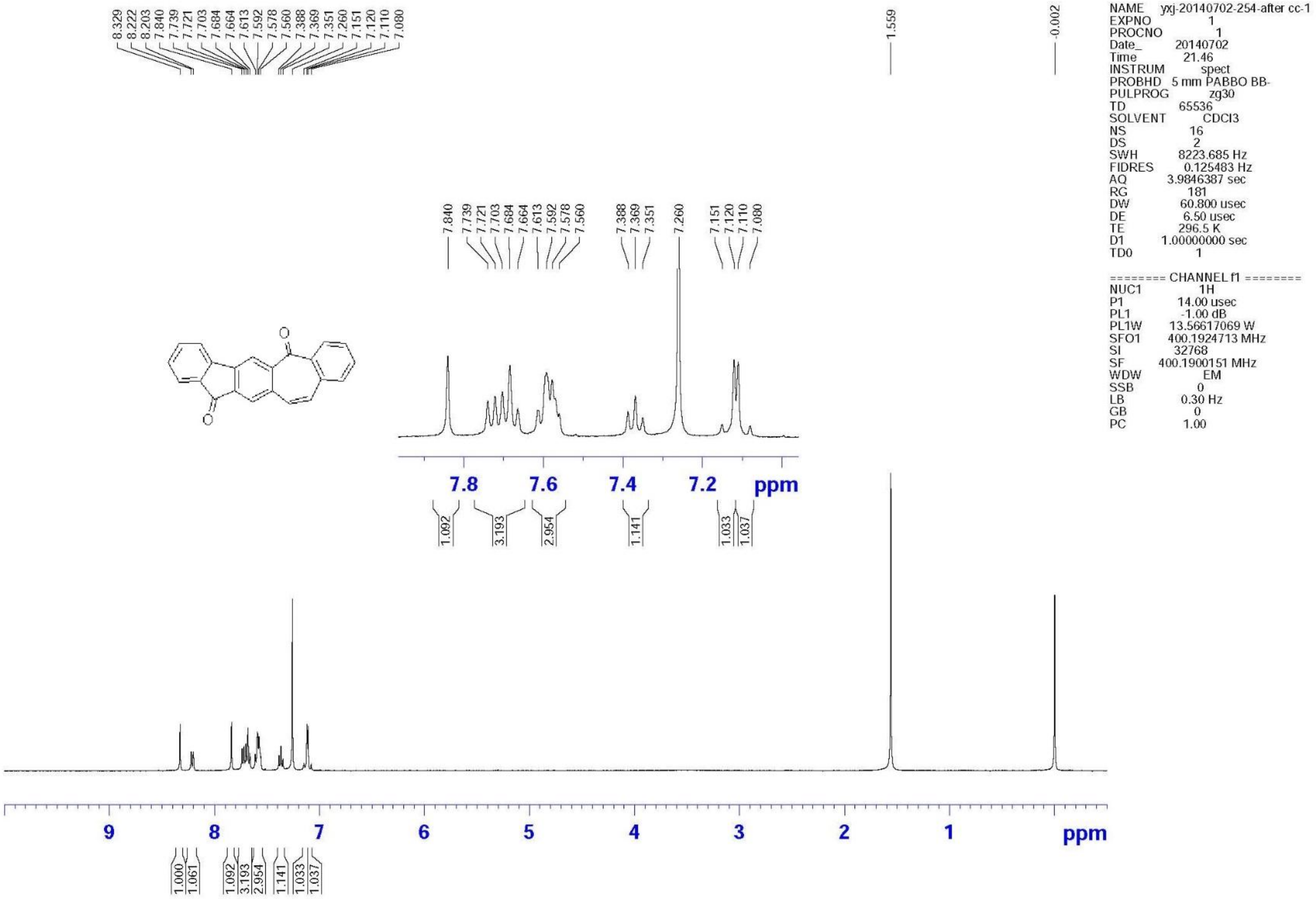

^{1}H NMR spectrum (400MHz, $CDCl_3$) of benzo[4,5]cyclohepta[1,2-*b*]fluorene-5,11-dione **(11)**

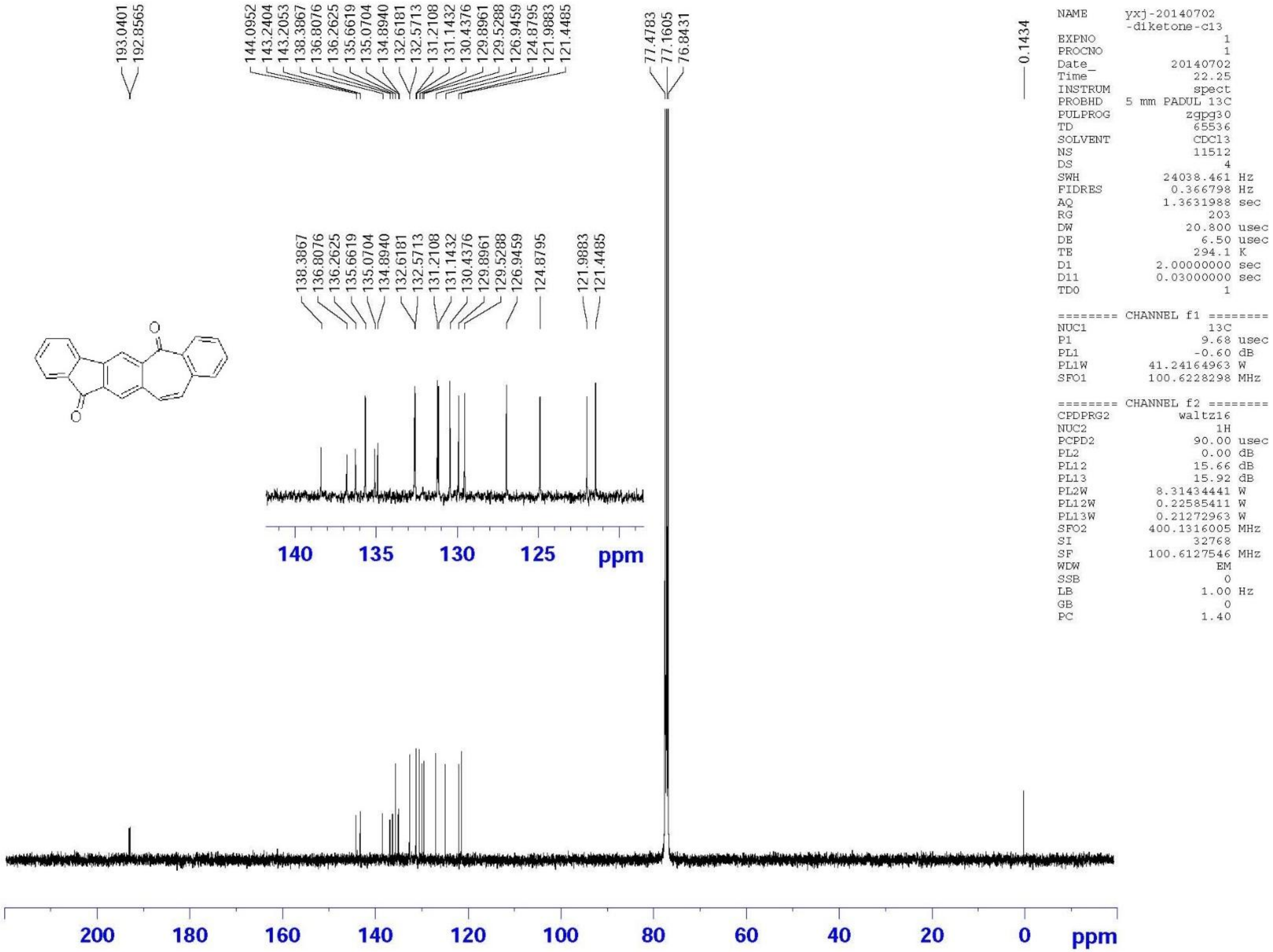

^{13}C NMR spectrum (100MHz, $CDCl_3$) of benzo[4,5]cyclohepta[1,2-*b*]fluorene-5,11-dione **(11)**

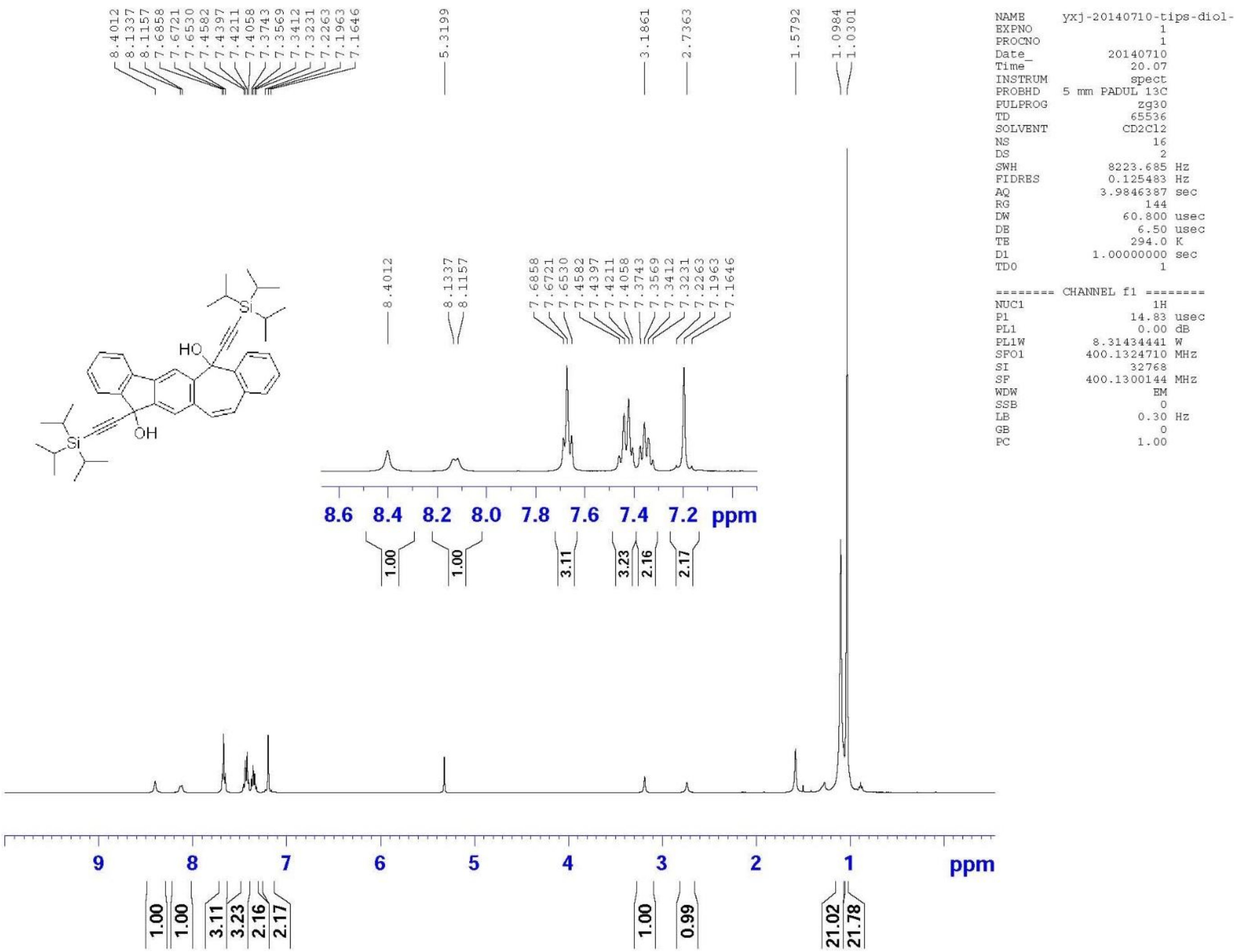

^{1}H NMR spectrum (400MHz, CD_2Cl_2) of diastereomer I of **12b**

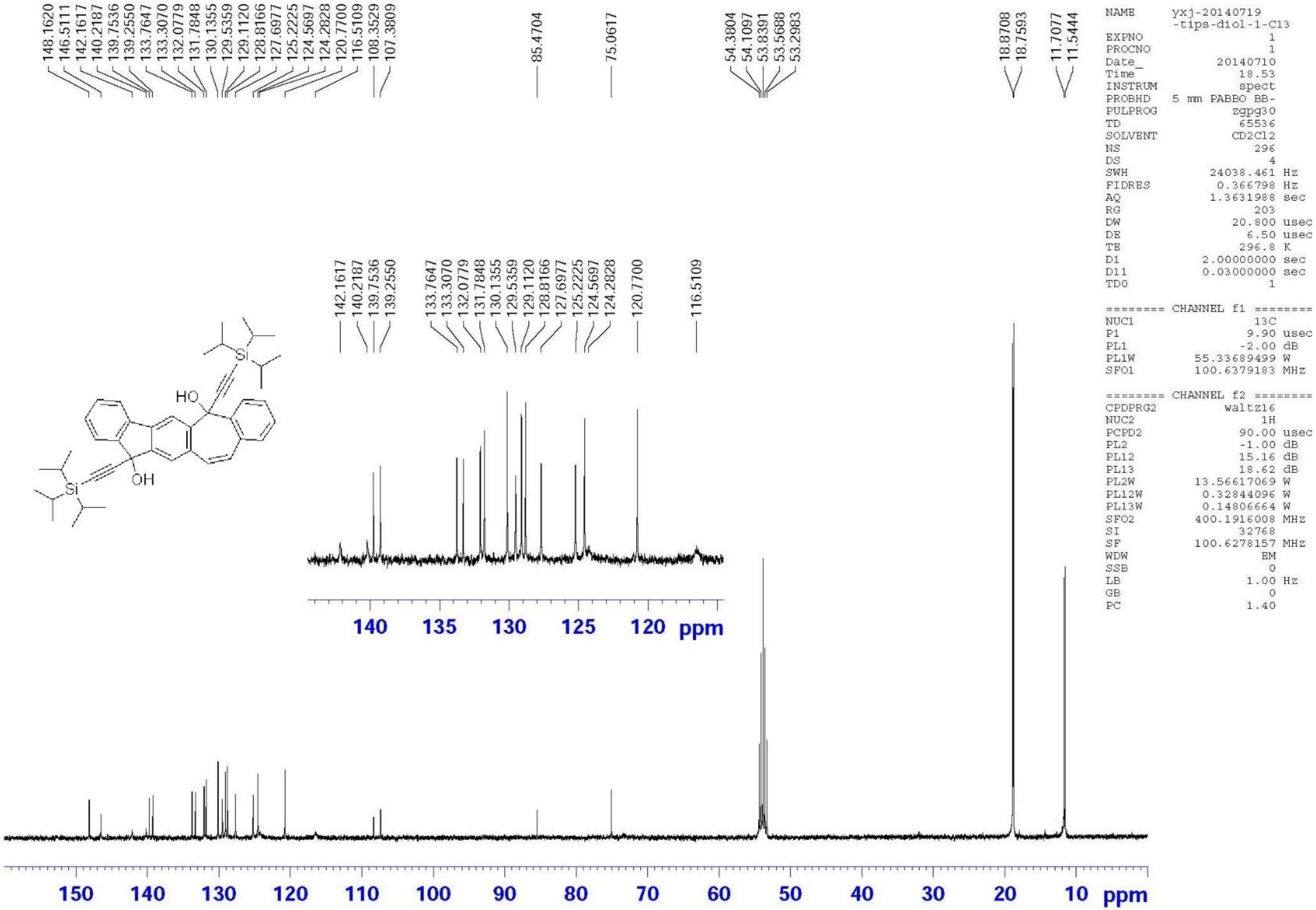

^{13}C NMR spectrum (100MHz, CD_2Cl_2) of diastereomer I of **12b**

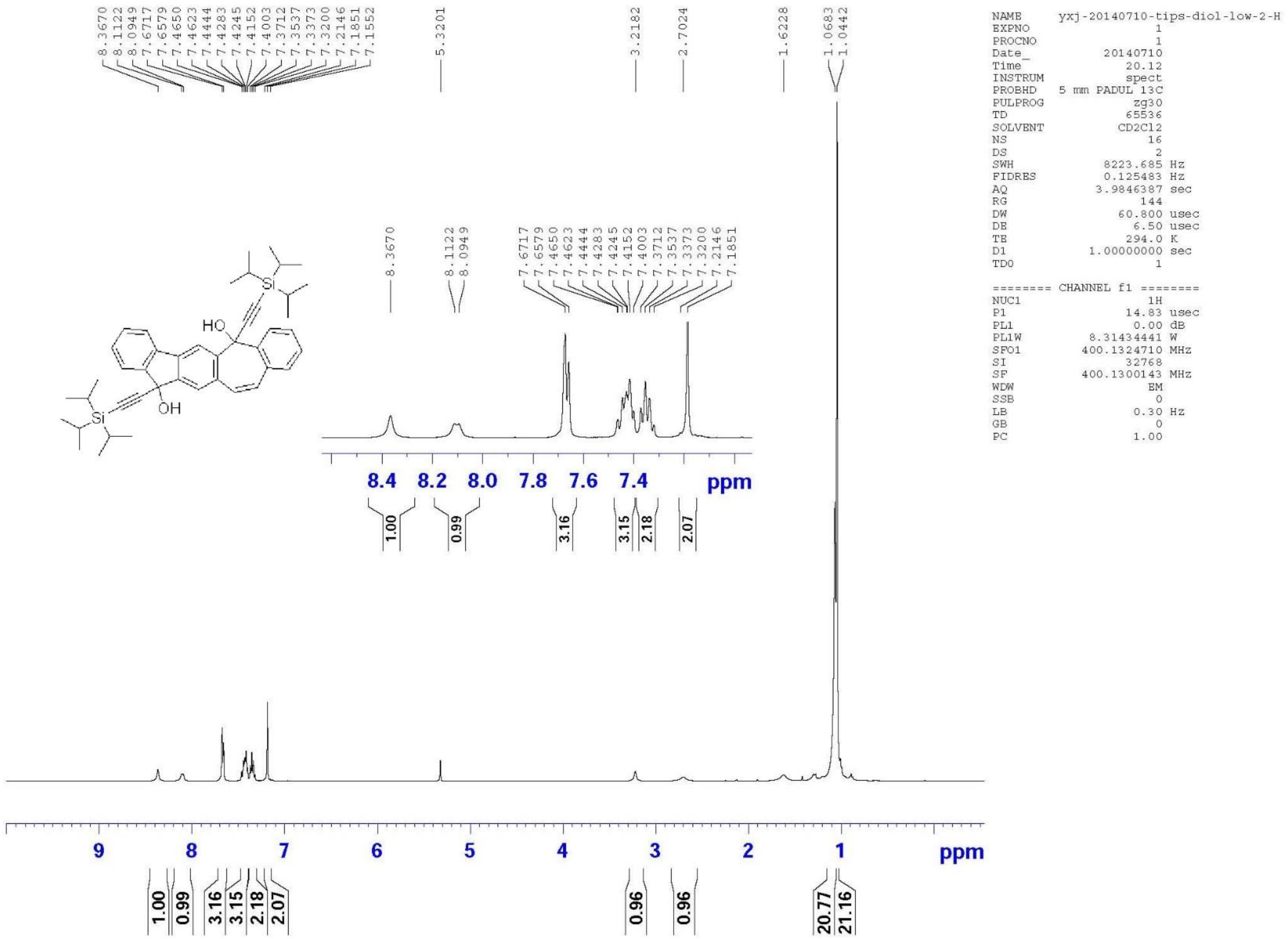

^{1}H NMR spectrum (400MHz, CD_2Cl_2) of diastereomer II of **12b**

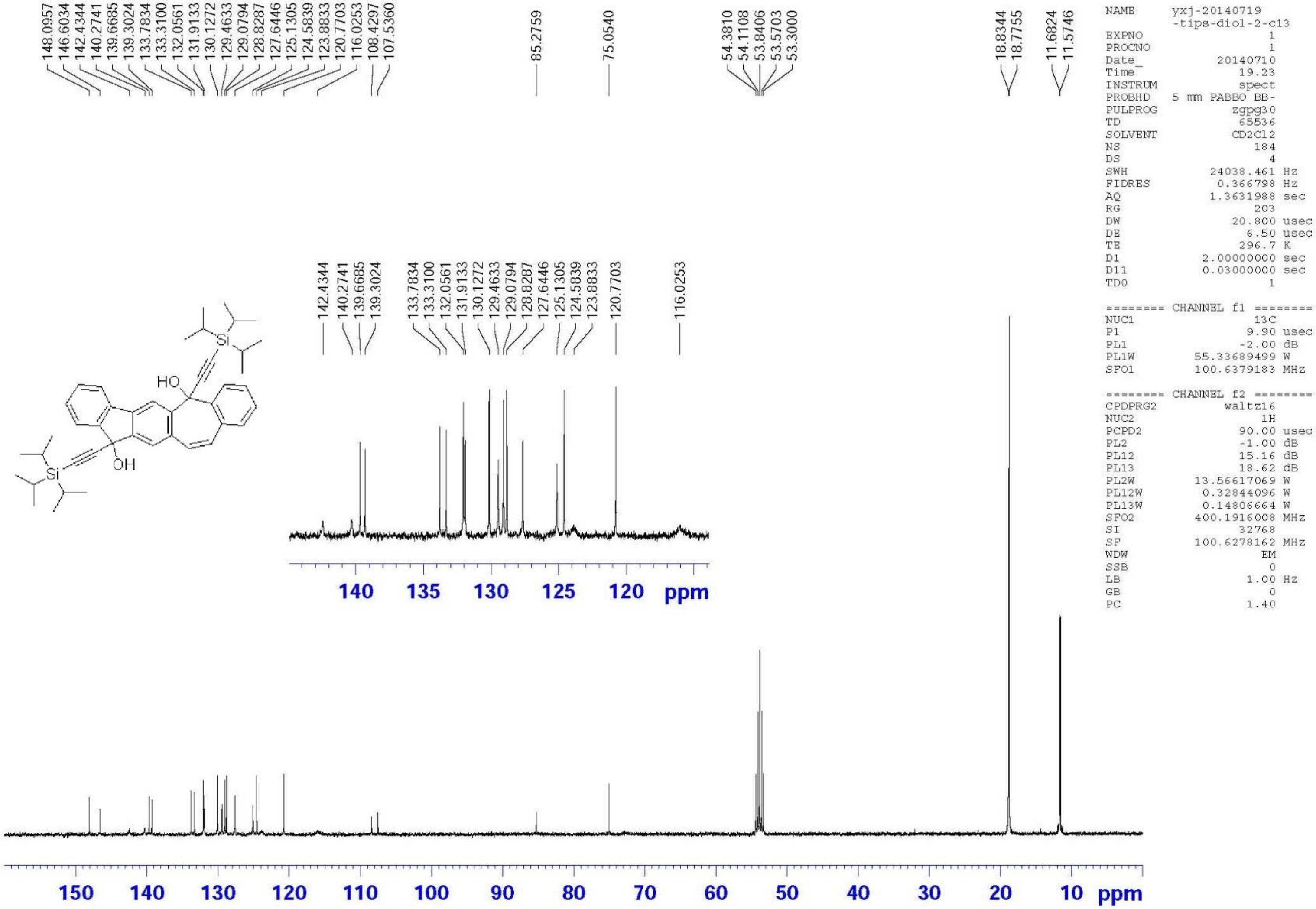

^{13}C NMR spectrum (100MHz, CD_2Cl_2) of diastereomer II of **12b**

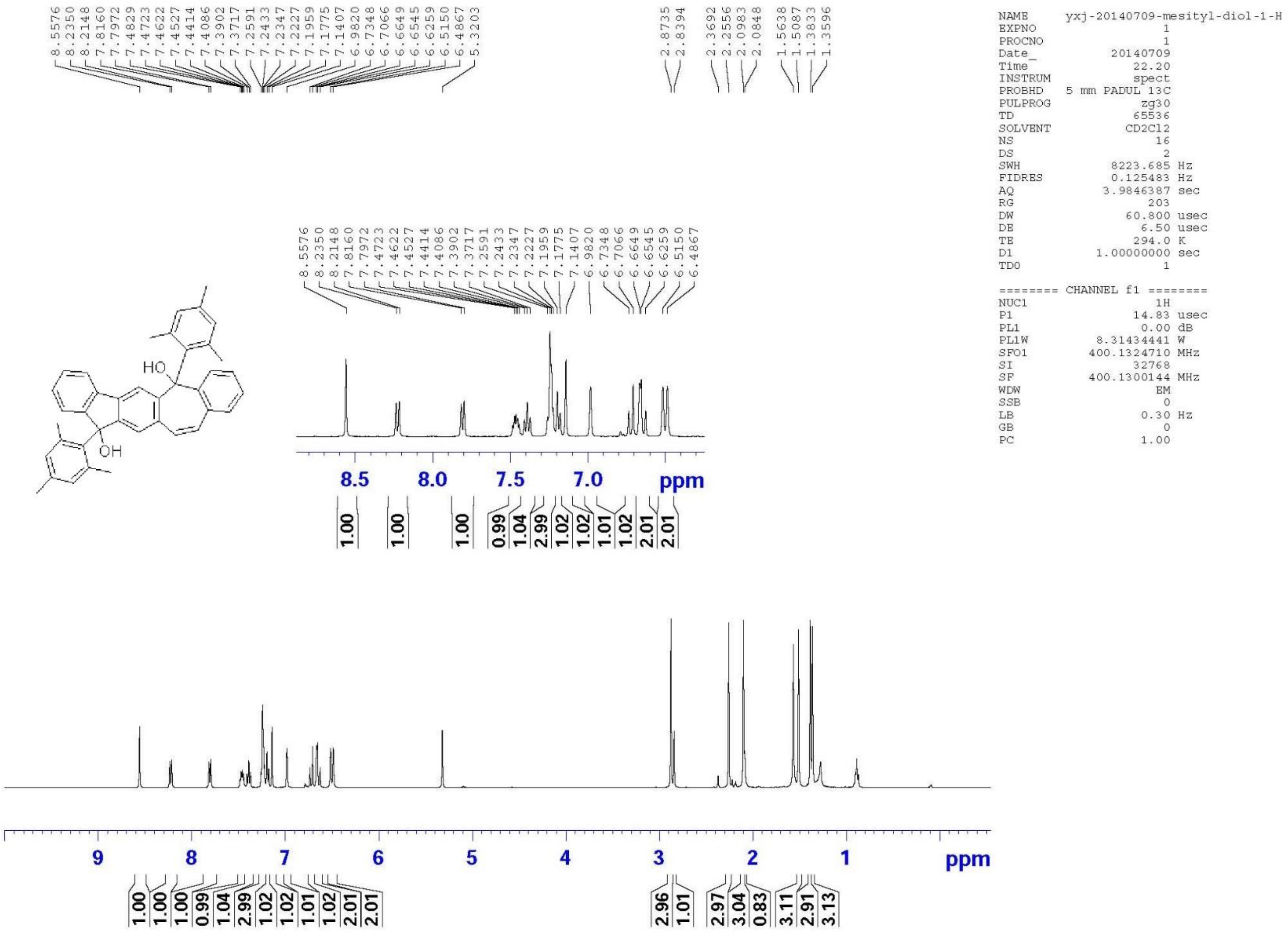

^{1}H NMR spectrum (400MHz, CD_2Cl_2) of diastereomer I of **12c**

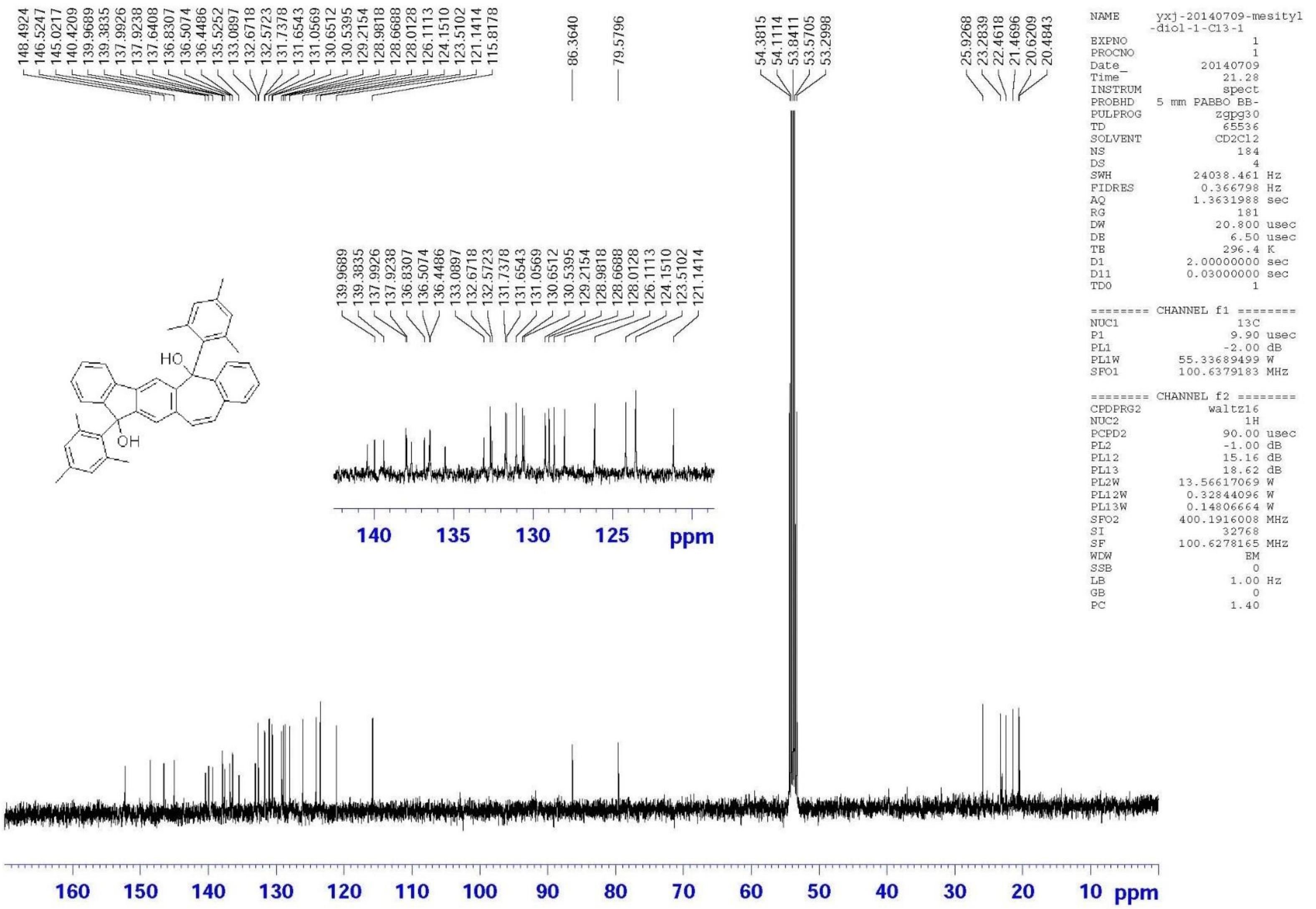

^{13}C NMR spectrum (100MHz, CD_2Cl_2) of diastereomer I of **12c**

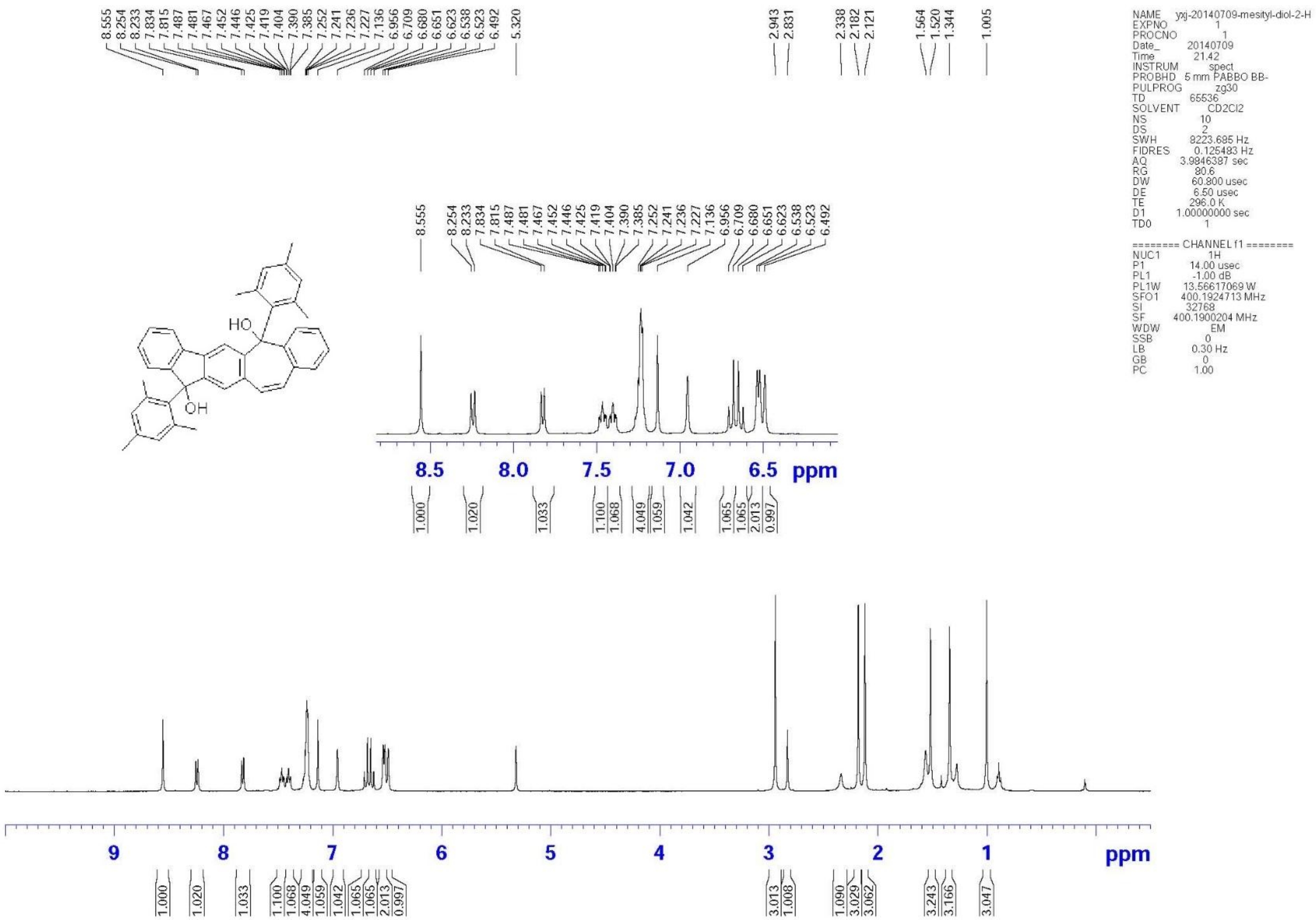

^{1}H NMR spectrum (400MHz, CD_2Cl_2) of diastereomer II of **12c**

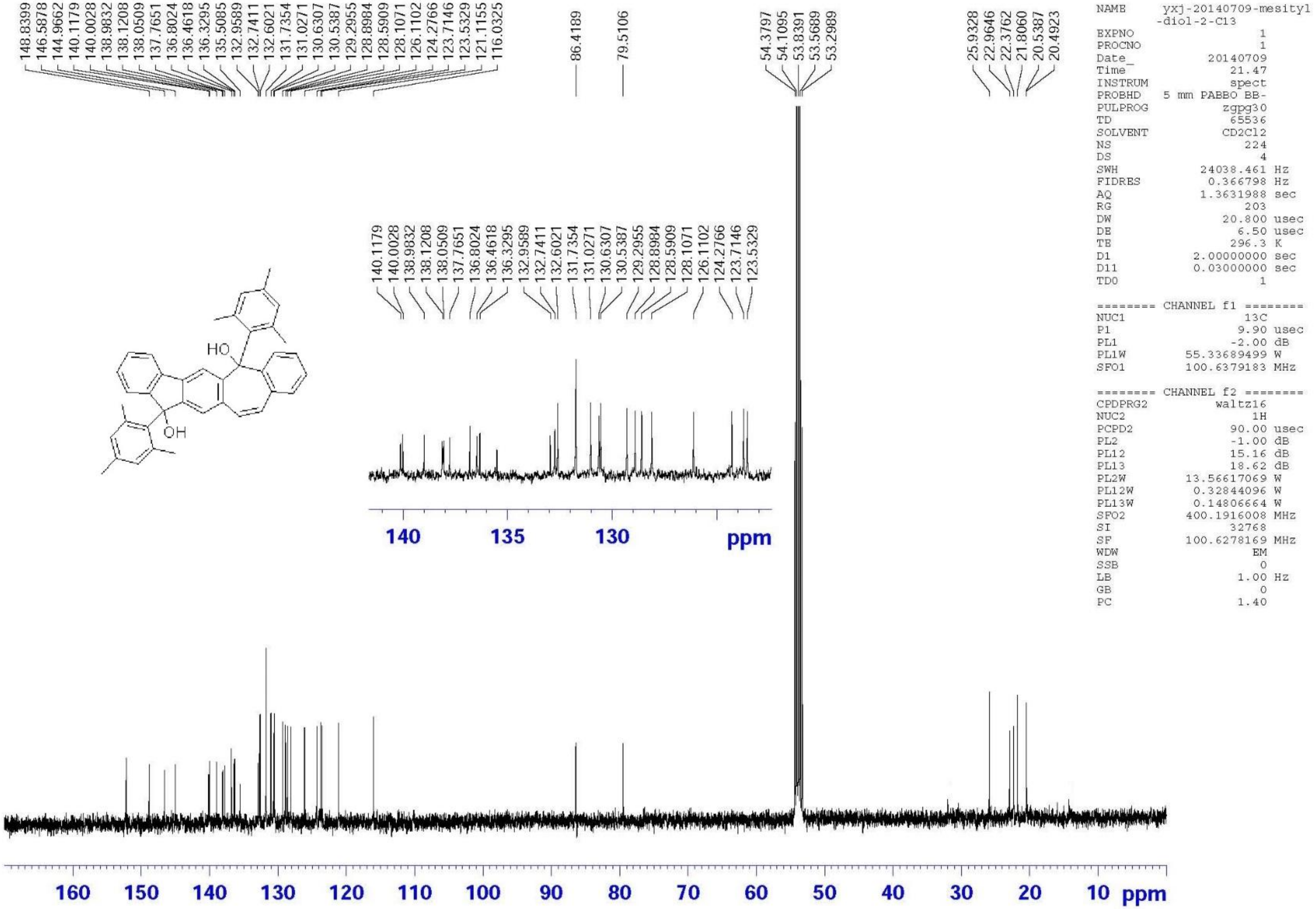

^{13}C NMR spectrum (100MHz, CD_2Cl_2) of diastereomer II of **12c**

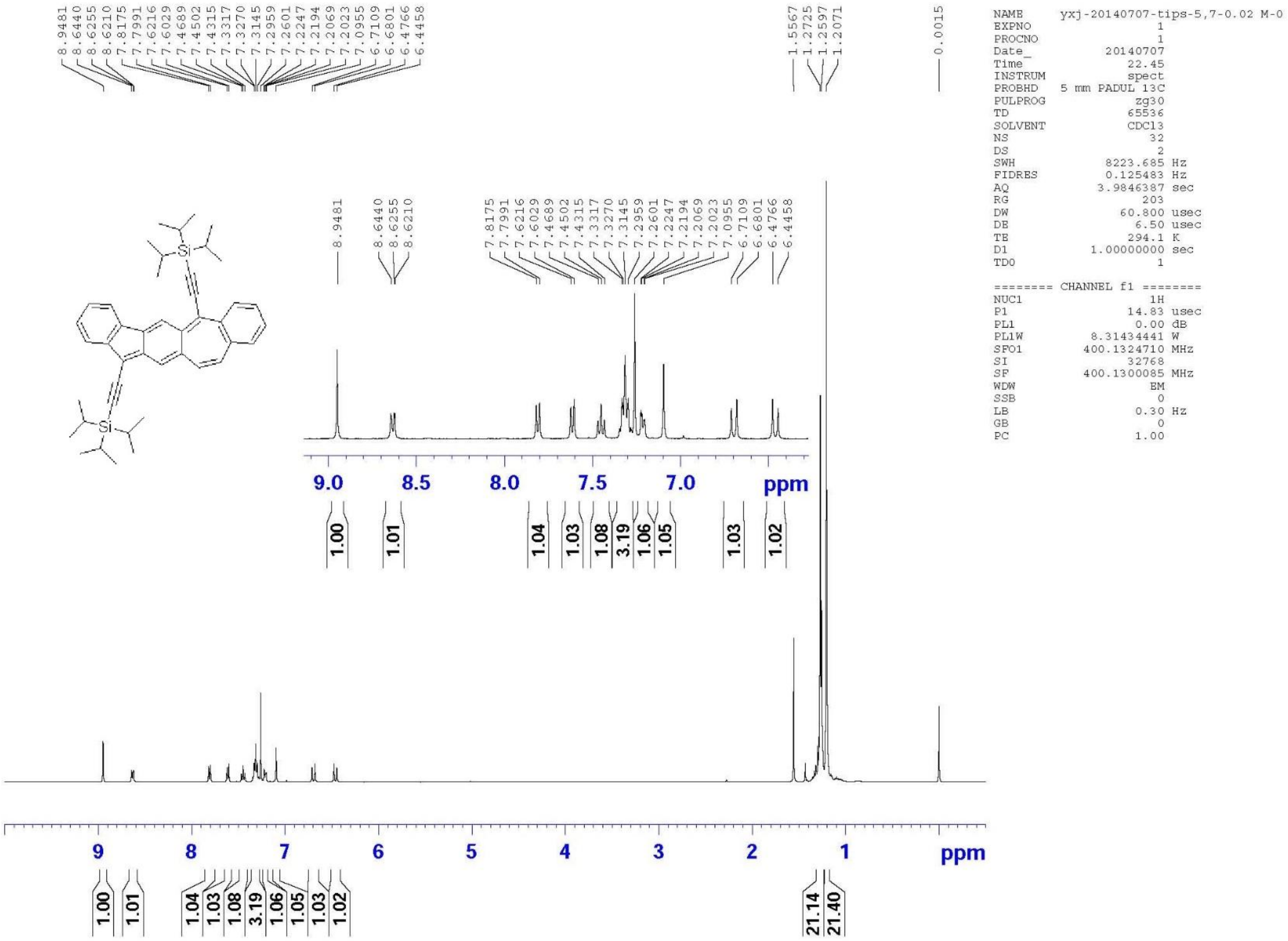

^{1}H NMR spectrum (400MHz, $CDCl_3$) of **5b**

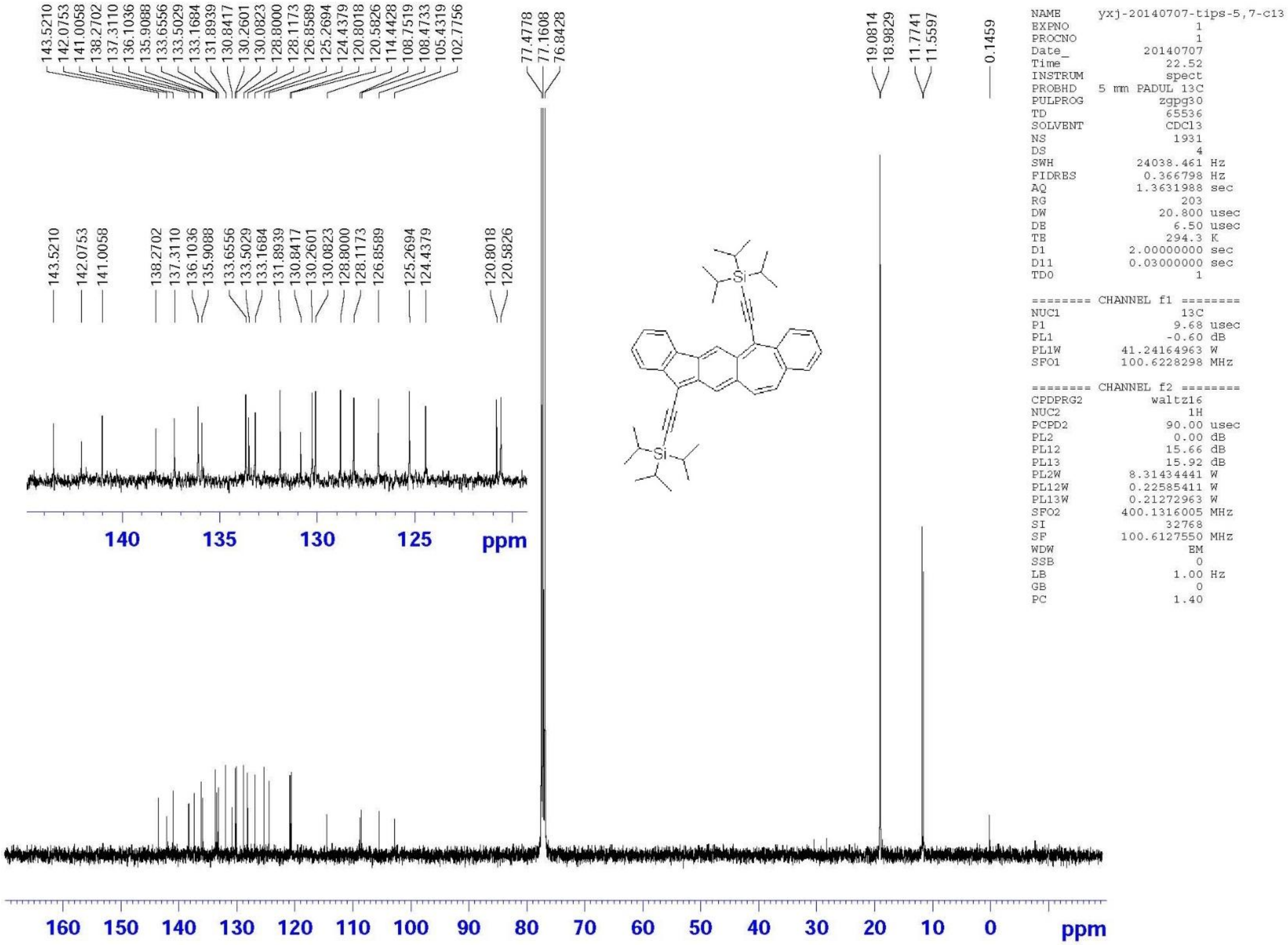

^{13}C NMR spectrum (100MHz, $CDCl_3$) of **5b**

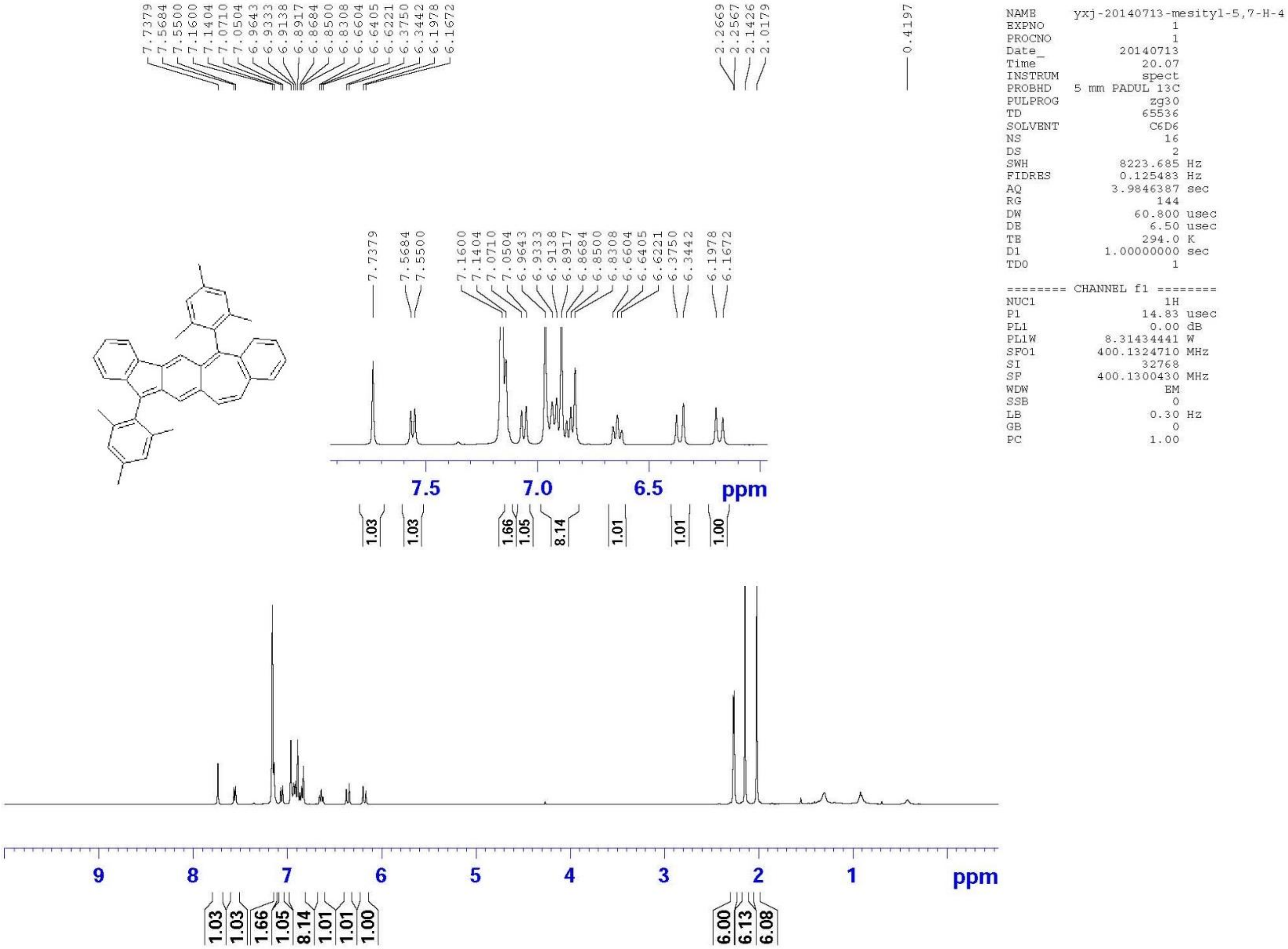

^{1}H NMR spectrum (400MHz, C_6D_6) of **5c**

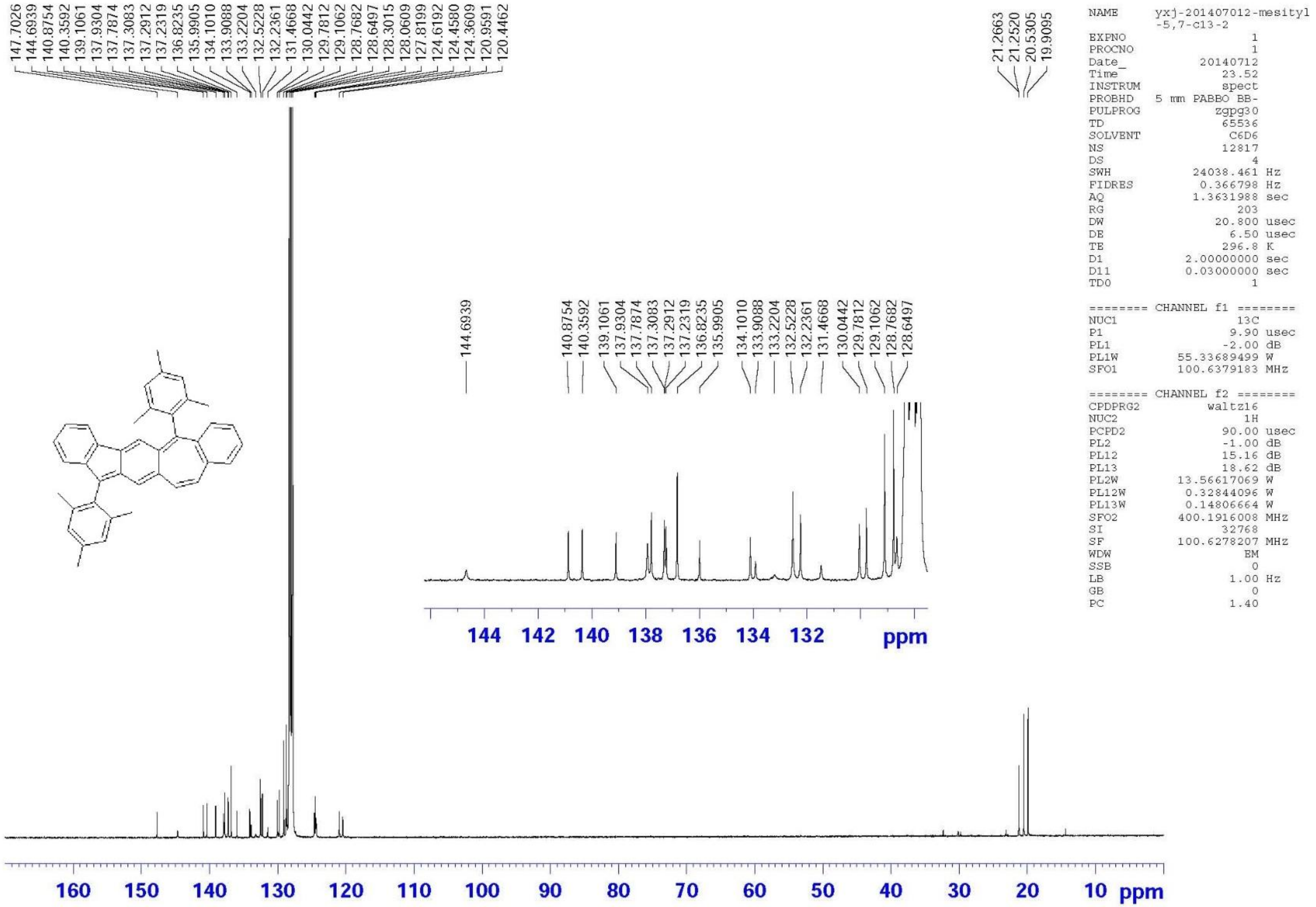

^{13}C NMR spectrum (100MHz, C_6D_6) of **5c**

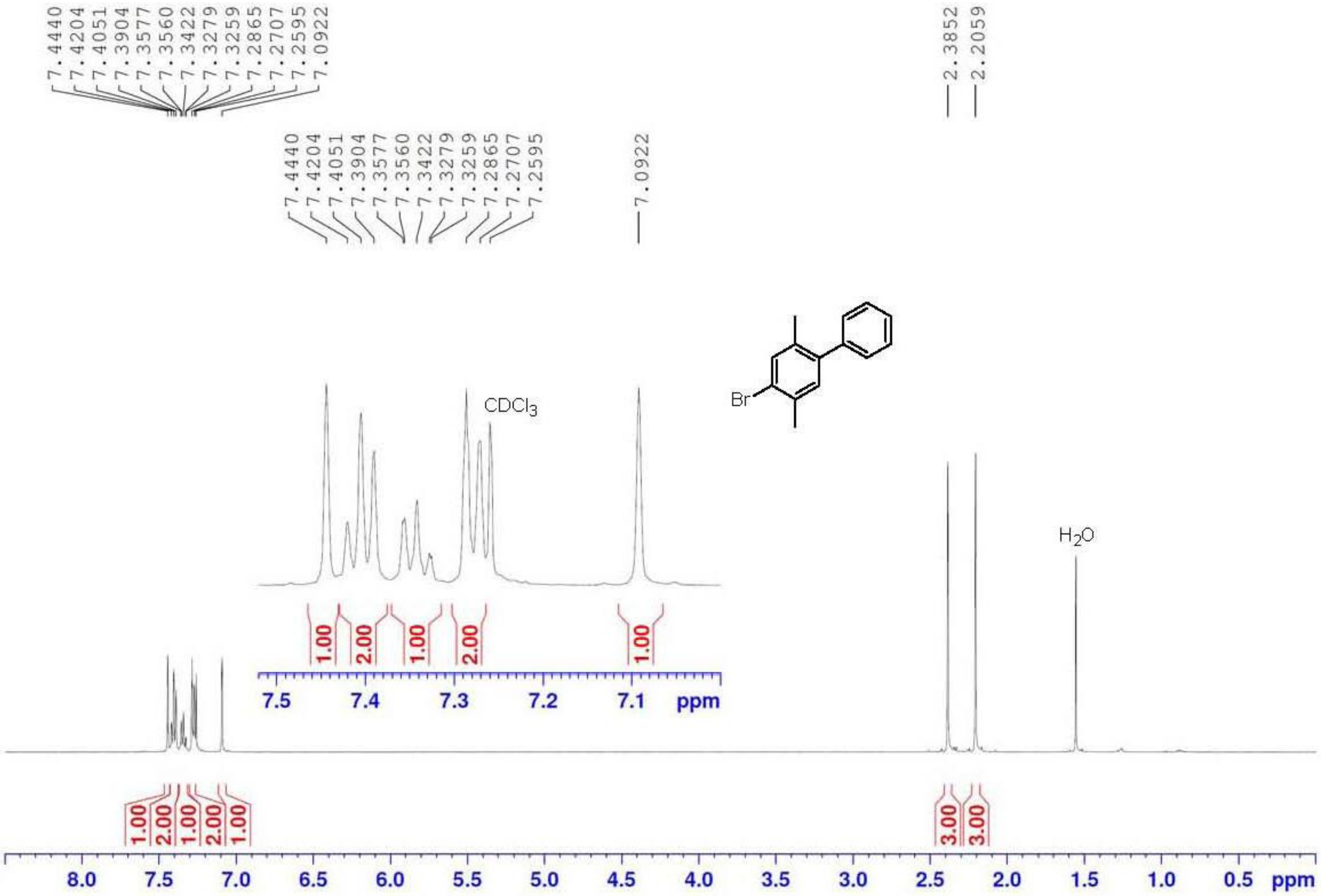

^{1}H NMR spectrum (500MHz, $CDCl_3$) of 4-bromo-2,5-dimethyl-1,1'-biphenyl **(14)**

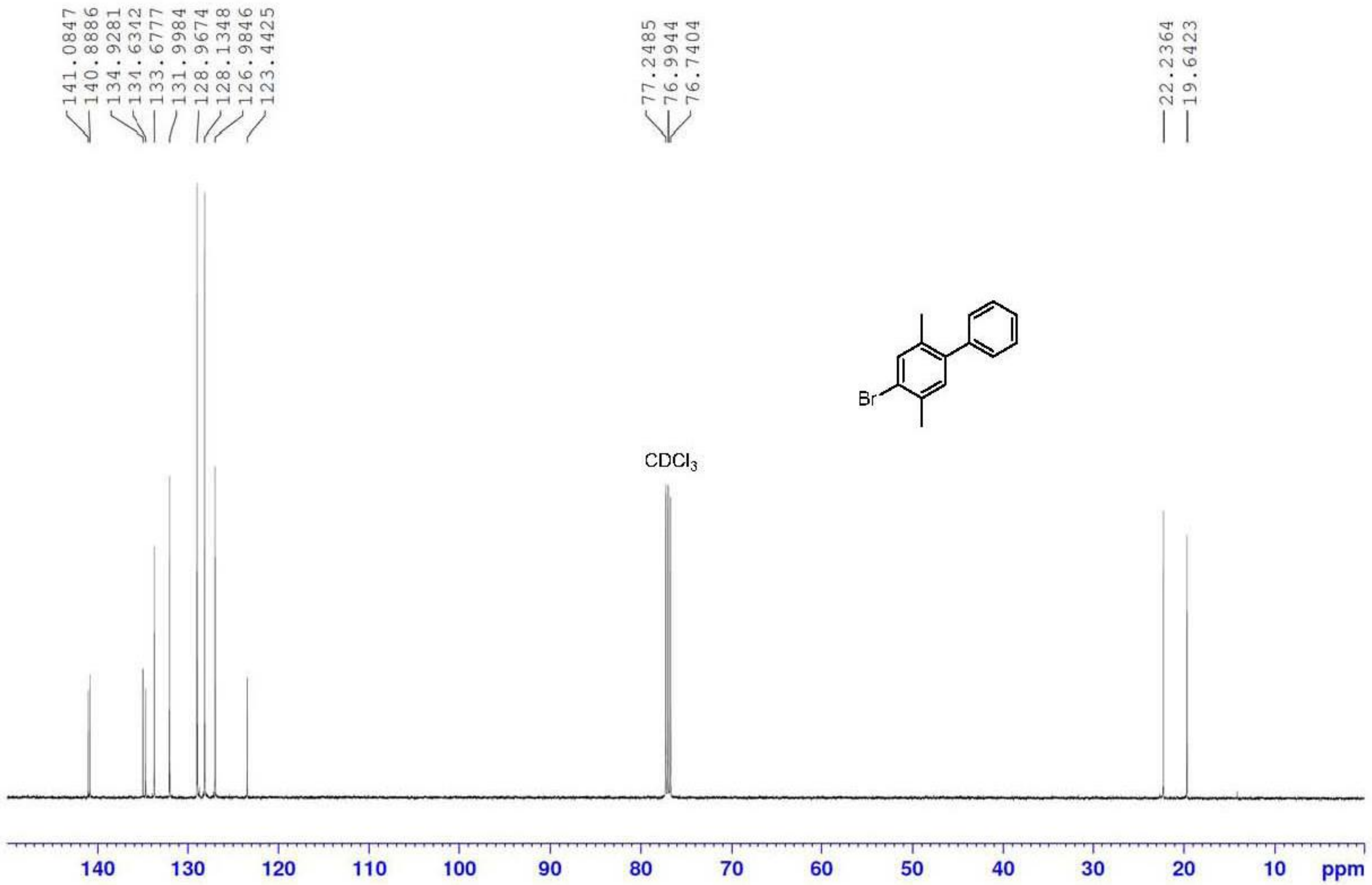

^{13}C NMR spectrum (125MHz, $CDCl_3$) of 4-bromo-2,5-dimethyl-1,1'-biphenyl **(14)**

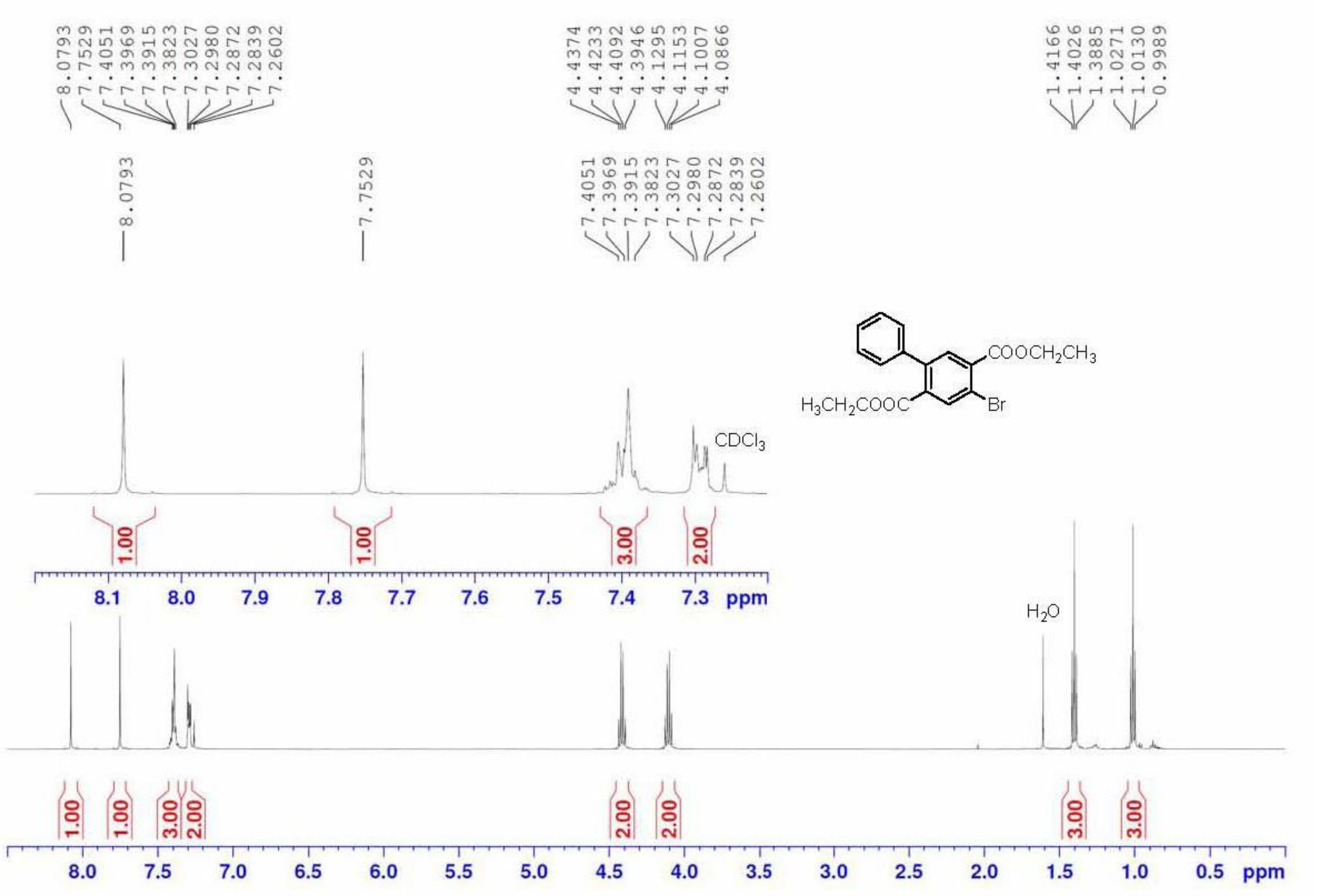

^{1}H NMR spectrum (500MHz, $CDCl_3$) of diethyl 4-bromo-[1,1'-biphenyl]-2,5-dicarboxylate **(15)**

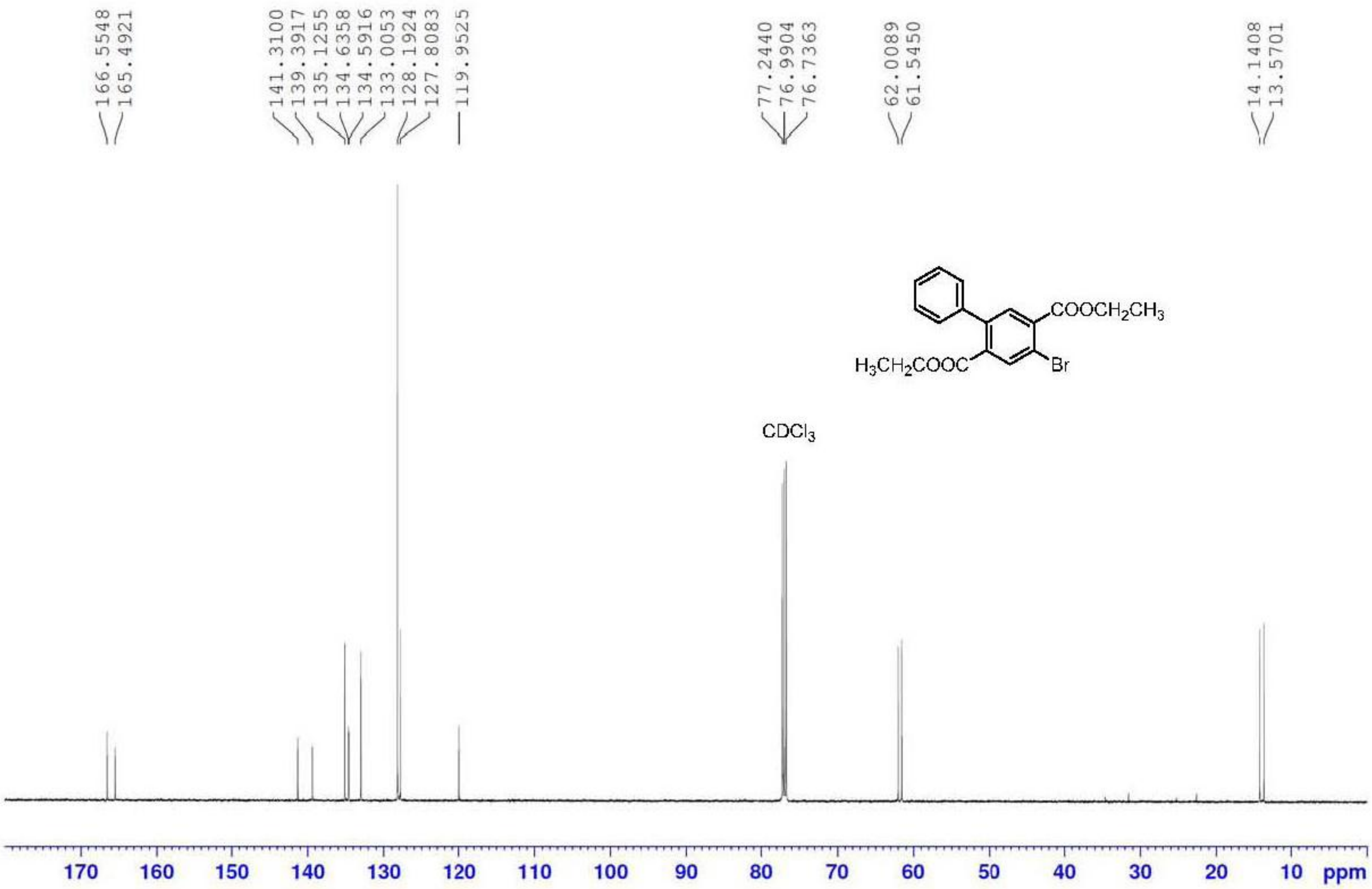

^{13}C NMR spectrum (125 MHz, $CDCl_3$) of diethyl 4-bromo-[1,1'-biphenyl]-2,5-dicarboxylate **(15)**

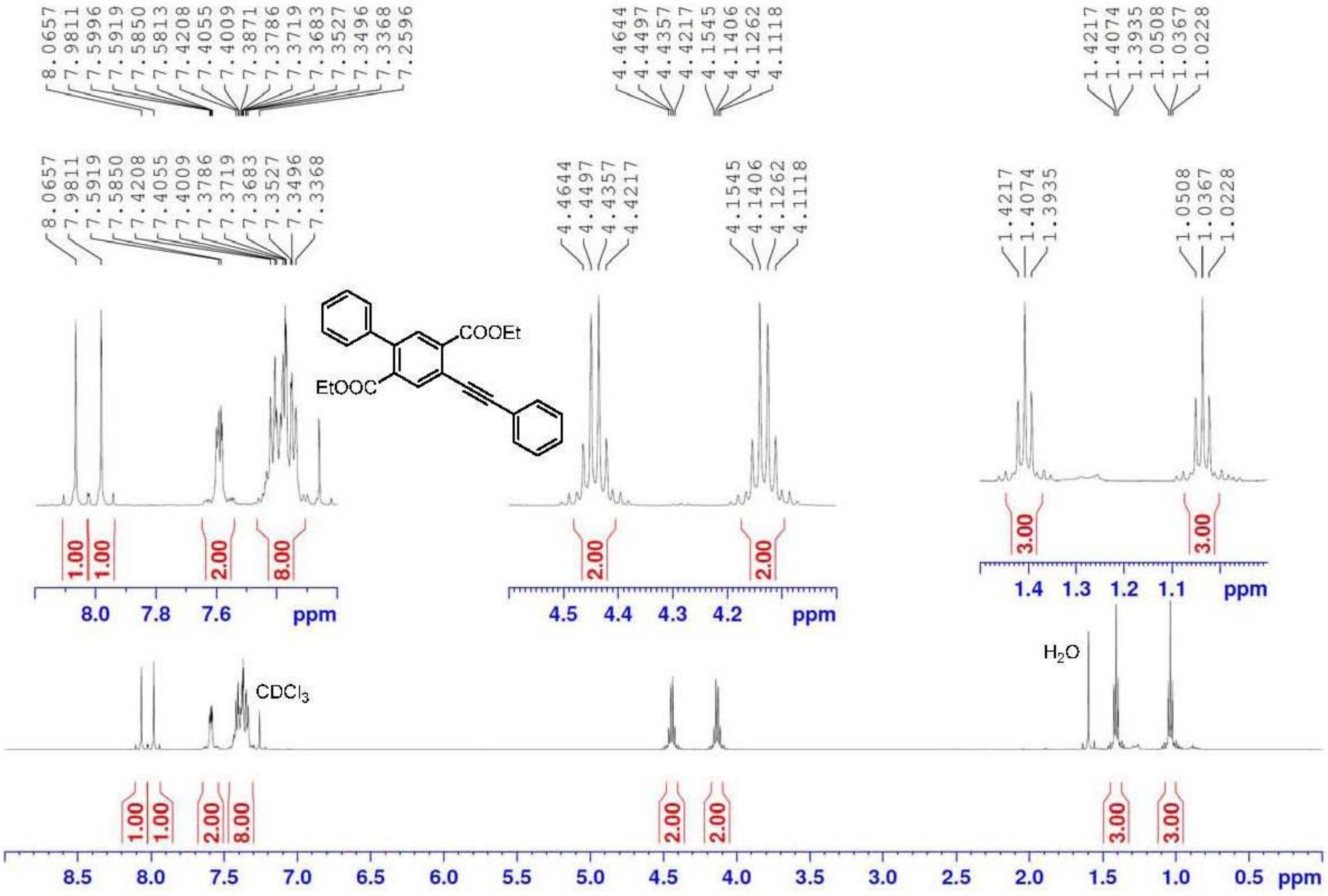

^{1}H NMR spectrum (500MHz, $CDCl_3$) of diethyl 4-(phenylethynyl)-[1,1'-biphenyl]-2,5-dicarboxylate **(16)**

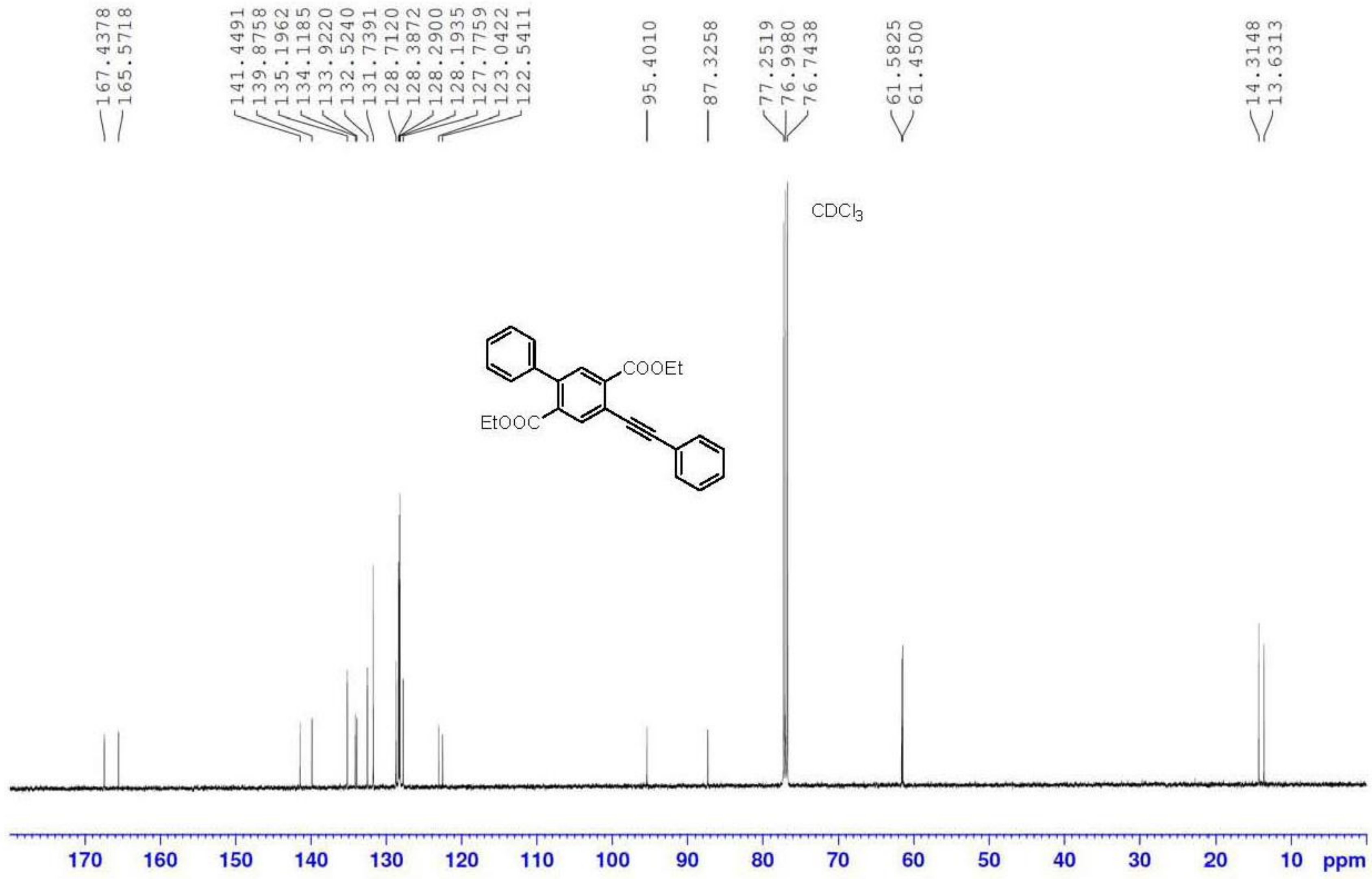

^{13}C NMR spectrum (125MHz, $CDCl_3$) of diethyl 4-(phenylethynyl)-[1,1'-biphenyl]-2,5-dicarboxylate **(16)**

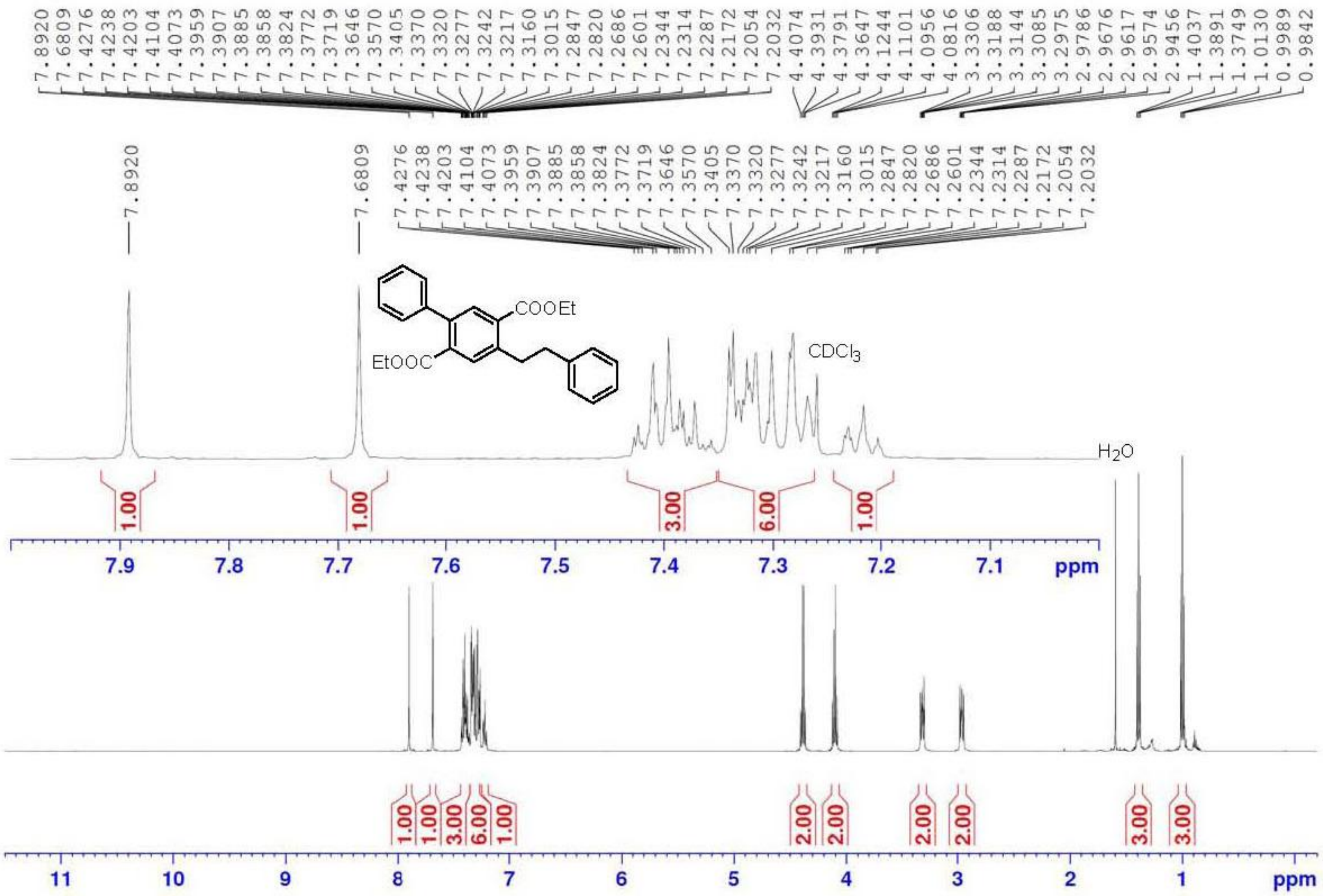

^{1}H NMR spectrum (500MHz, $CDCl_3$) of diethyl 4-phenethyl-[1,1'-biphenyl]-2,5-dicarboxylate **(17)**

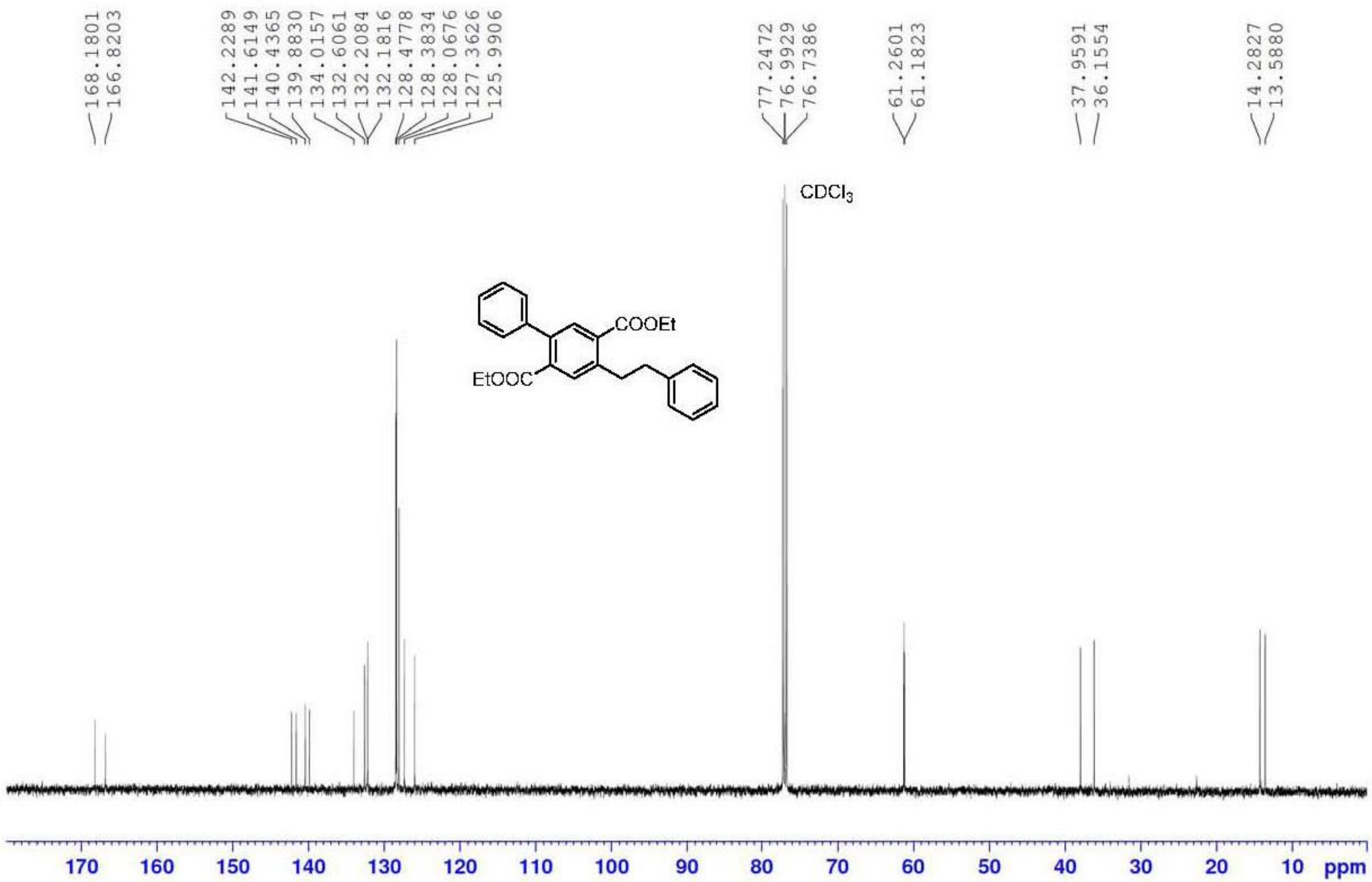

^{13}C NMR spectrum (125MHz, $CDCl_3$) of diethyl 4-phenethyl-[1,1'-biphenyl]-2,5-dicarboxylate **(17)**

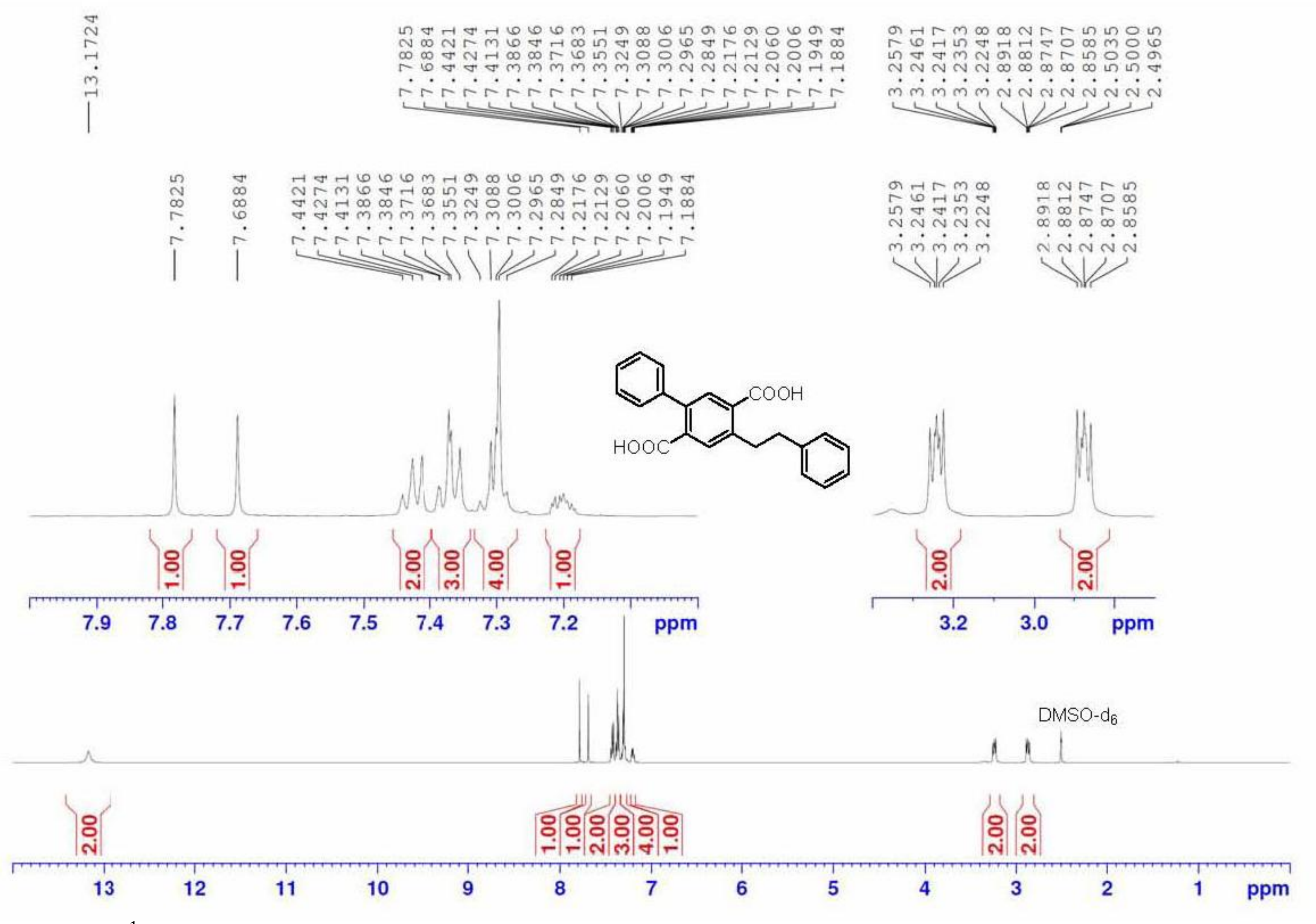

^{1}H NMR spectrum (500MHz, DMSO-d_6) of 4-phenethyl-[1,1'-biphenyl]-2,5-dicarboxylic acid **(18)**

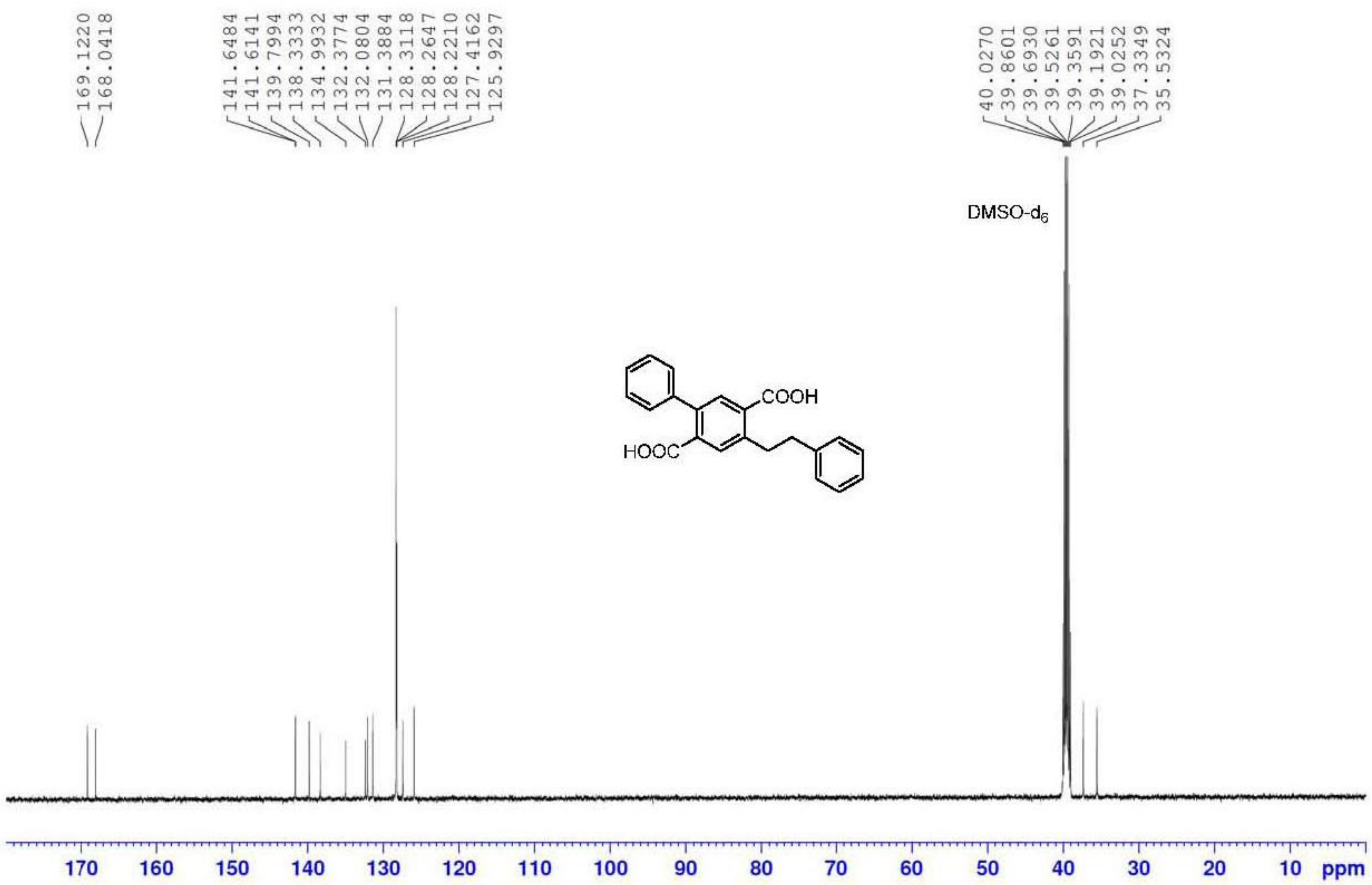

^{13}C NMR spectrum (125MHz, DMSO-d_6) of 4-phenethyl-[1,1'-biphenyl]-2,5-dicarboxylic acid **(18)**
